# Sockeye salmon repatriation leads to population re‐establishment and rapid introgression with native kokanee

**DOI:** 10.1111/eva.12430

**Published:** 2016-10-21

**Authors:** Andrew J. Veale, Michael A. Russello

**Affiliations:** ^1^ Department of BiologyThe University of British ColumbiaKelownaBCCanada; ^2^ Present address: Department of ZoologyUniversity of Otago340 Great King StreetDunedinNew Zealand

**Keywords:** fisheries management, hybridization, *Oncorhynchus nerka*, reintroduction, single nucleotide polymorphism

## Abstract

Re‐establishing salmonid populations to areas historically occupied has the substantial potential for conservation gains; however, such interventions also risk negatively impacting native resident stocks. Here, we assessed the success of the hatchery‐assisted reintroduction of anadromous sockeye salmon (*Oncorhynchus nerka*) into Skaha Lake, British Columbia, Canada, and evaluated the genetic consequences for native kokanee, a freshwater‐obligate ecotype, using single nucleotide polymorphism genotypic data collected from the reference samples of spawning Okanagan River sockeye and Skaha Lake kokanee presockeye reintroduction, along with annual trawl survey and angler‐caught samples obtained over an eight‐year period. Significant differentiation was detected between sockeye and kokanee reference samples, with >99% stock assignment. Low proportions of sockeye and hybrids were detected within 2008 and 2010 age‐0 trawl samples; however, by 2012, 28% were sockeye, rising to 41% in 2014. The number of hybrids detected rose proportionally with the increase in sockeye and exhibited an intermediate phenotype. Our results indicate that the reintroduction of anadromous sockeye to Skaha Lake is succeeding, with large numbers returning to spawn. However, hybridization with native kokanee is of concern due to the potential for demographic or genetic swamping, with ongoing genetic monitoring necessary to assess the long‐term effects of introgression and to support interactive fisheries management.

## 1 Introduction

Reintroducing species to parts of their former range from which they have been extirpated is becoming an increasingly common practice, both for conservation goals and due to the social and economic values of these species (Seddon, Armstrong, & Maloney, 2007). There are several genetic factors that must be considered when planning a reintroduction, particularly from a captive breeding program (Anderson et al., 2014; Fraser, 2008; Utter, 2004). These factors include (i) choosing the appropriate genetic stock to reintroduce; (ii) maintaining genetic diversity and adaptive potential in the reintroduced population; (iii) conserving the genetic diversity and fitness of the original stock; (iv) minimizing genetic adaptation to domestication; and (v) ensuring that the introduced stock does not have negative genetic consequences for other sympatric populations through introgression (Fraser, 2008; Theodorou & Couvet, 2004; Utter, 2000; Waples, 1991). This last issue of identifying risks posed by hybridization can be particularly difficult to assess in cases where populations are genetically similar, but ecologically divergent.

If hybridization does occur between a reintroduced population and an existing population, there are several possible evolutionary outcomes (Harrison & Larson, 2014; Rhymer & Simberloff, 1996; Stebbins, 1959). Hybridization may have positive effects through an increased genetic diversity, which may include the transfer of adaptive genes, or provide a genetic rescue for the population if it was significantly inbred. Alternatively, it may have negative consequences whereby the introgressed population declines, potentially to extinction through demographic or genetic swamping. Following the terminology of Wolf, Takebayashi, and Rieseberg (2001), demographic swamping occurs when the fitness of hybrids is significantly reduced (outbreeding depression), and the effect of this loss of recruitment on the introgressed population is a decline to extinction. Alternatively, genetic swamping occurs when the magnitude of admixture is so high that eventually one or both populations are fully replaced by hybrids, leading to the extinction of a pure and distinct population. The level of divergence between the populations, the relative fitness of hybrid individuals, and the magnitude of hybridization will largely determine the outcomes of hybridization on a population (Todesco et al., 2016).

In response to declining returns of Pacific Salmon (*Oncorhynchus* spp) combined with significant contractions of their spawning range, hatchery conservation programs are increasingly being employed to reintroduce or maintain local populations (Anderson, Faulds, Atlas, & Quinn, 2013; Flagg, Mahnken, & Johnson, 1995; Fraser, 2008; Kozfkay et al., 2008; Nickelson, Solazzi, & Johnson, 1986; Sard et al., 2015). These reintroduction or restocking programs are generally undertaken while the underlying mechanisms responsible for population decline, such as the damming of migratory pathways and habitat degradation, are addressed (Pess, Quinn, Gephard, & Saunders, 2014; Roni, Beechie, Leonetti, Pollock, & Pess, 2002).

**Figure 1:**
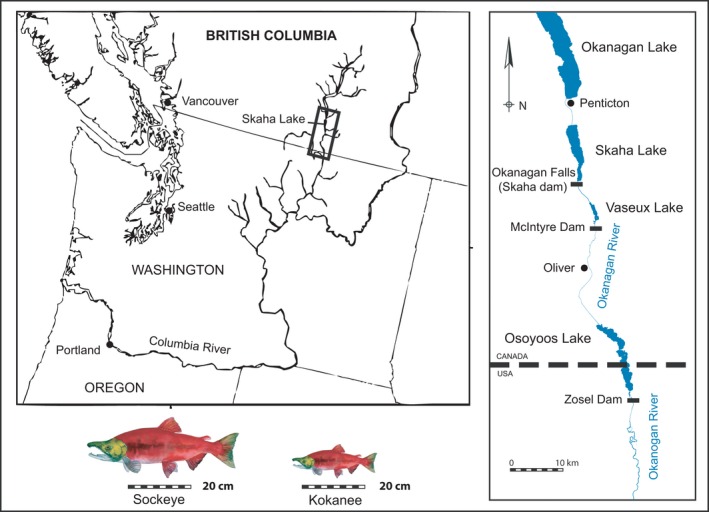
Map showing the northern reaches of the Columbia River with inset map (right) showing Skaha Lake and nearby rivers and dams mentioned in the text. Also shown is a size comparison between an average sockeye jack and an average kokanee jack

Within the Columbia River Basin, around 30% of salmonid populations have been extirpated and many of the remaining populations are listed as endangered or threatened (Gustafson et al., 2007). Prior to the early 20th century, a portion of the anadromous sockeye salmon population (*Oncorhynchus nerka*) spawning in the Okanagan Basin (part of the northern Columbia Basin) continued north into British Columbia (BC) and, depending on river flow conditions, topped the rapids at Okanagan Falls making their way into Skaha and Okanagan lakes (Ernst, 2000; Hewes, 1998; Kennedy & Bouchard, 1998; Long, 2005; Figure 1). Construction of the McIntyre Dam in 1916 effectively excluded the passage of these anadromous sockeye into Skaha Lake and the upper Okanagan Basin (Figure 1). Moreover, continued European settlement in the early and mid‐19th century brought other pressures on the salmon stock including agriculture, water engineering, and overfishing.

In 1997, the Okanagan Nation Alliance and Colville Confederated Tribes began to explore the possibility of bringing sockeye salmon back to the Okanagan Valley. After three years (2000‐03) of risk assessment and project design, the Canadian Okanagan Basin Technical Working Group determined that sockeye salmon should be reintroduced into Skaha Lake (Parnell, Peters, & Marmorek, 2003; Peters, Bernard, & Marmorek, 1998). To do this, a number of management interventions were required. Flow management was initiated to better balance the needs of sockeye salmon smolt and adults with the recreation, irrigation, flood control, and native kokanee interests (Hyatt & Stockwell, 2013). Sockeye passage was provided at McIntyre Dam and hatchery production was initiated to reintroduce sockeye salmon fry into Skaha Lake itself beginning in 2004, with the ultimate goal of restoring passage of migrants to and from this lake at all flows (McQueen et al., 2013). In years of high flow, adults can now successfully migrate upstream through the Skaha Dam spillway and juveniles can pass down the spillway at all flow levels. Finally, restoration of meanders has begun in parts of the river channelized in the 1950s by setting back dikes and opening up access to previously cutoff areas.

The management strategy for this reintroduction program specifically focused on minimizing the potential negative genetic consequences caused by reintroducing the species through hatchery restocking (Peters et al., 1998; Wright & Smith, 2004). This was achieved by using broodstock from immediately below the dam in the Okanagan River near Oliver, BC, which constitutes the population that continued north into Skaha Lake prior to dam construction. Since the time sockeye salmon hatchery restocking in Skaha Lake began in 2004, efforts have been made to maintain genetic diversity of the reintroduced stock by using large numbers of founders each year (500,000–1,600,000 fry released into the lake per year), and by not keeping captive broodstock—every year new broodstock has been obtained from returning salmon in the Okanagan River (McQueen et al., 2013).

There are several morphologically and ecologically divergent ecotypes of sockeye salmon, with anadromous sockeye ecotypes spawning in freshwater then migrating out to sea and freshwater‐obligate ecotypes (kokanee) existing entirely in lakes (Dodson, Aubin‐Horth, Theriault, & Paez, 2013; Taylor, Foote, & Wood, 1996; Wood, Bickham, Nelson, Foote, & Patton, 2008). There are further subdivisions of sockeye ecotypes, with various spawning habitat preferences, migration timings, and spawning periods for both anadromous sockeye and kokanee. Kokanee are considerably smaller than sockeye (~26 cm vs. >45 cm average adult fork length; Figure 1), and in many lakes, they occur sympatrically with anadromous sockeye. In Skaha Lake, both the extirpated anadromous sockeye and kokanee spawn around October, primarily in the Okanagan River (Penticton Channel), which flows into Skaha Lake from Okanagan Lake. There is a considerable potential for gene flow between ecotypes, as kokanee males sneak on spawning sockeye pairs (Foote & Larkin, 1988) and can fertilize up to 20% of a female sockeye's eggs (Foote, Brown, & Wood, 1997). Hybrid progeny are fully viable and fertile when raised in hatchery settings (Craig, Foote, & Wood, 2005); however, adaptive divergence associated with distinct life histories is thought to maintain genetic differentiation between sockeye and kokanee where they naturally occur in sympatry (Taylor et al., 1996; Wood et al., 2008).

The reintroduced sockeye populations in Skaha Lake have the potential for both demographic and genetic swamping of the resident kokanee. Previous studies have demonstrated the reduced fitness of sockeye/kokanee hybrids for a variety of traits associated with anadromous and freshwater resident life histories, including salinity tolerance, swimming performance, growth, and development (Foote, Wood, Clarke, & Blackburn, 1992; Wood & Foote, 1990, 1996). Due to the levels of interbreeding possible with the large numbers of sockeye being reintroduced, genetic swamping may also be a risk, as has happened in other salmonid hatchery‐based translocations (Spies, Anderson, Naish, & Bentzen, 2007).

Here, we used single nucleotide polymorphism (SNP) genotypic data collected from reference samples of spawning Okanagan River sockeye and Skaha Lake kokanee presockeye reintroduction, along with annual trawl survey and angler‐caught samples obtained over an eight‐year period to (i) quantify the success of the repatriation program for establishing a wild‐spawning anadromous sockeye salmon population in Skaha Lake and (ii) assess introgression trends between reintroduced anadromous sockeye and the indigenous kokanee population. We further demonstrate a rigorous framework for evaluating the power of molecular markers for detecting and quantifying introgression, and discuss the relevance of our empirical findings for fisheries management.

## 2 Methods

### 2.1 Sample collection and selection

The BC Ministry of Forests, Lands and Natural Resource Operations (BC MFLNRO) provided reference tissue (fin clip) from spawning Okanagan River sockeye (*n* = 148) in 2012 and from spawning Skaha Lake kokanee (*n* = 130) in 2003, prior to the hatchery restocking program (Table 1).

**Table 1:** Sampling scheme for nonthermal marked *Oncorhynchus nerka* in Skaha Lake

Sample year	Sampling period	Type	Age	Sample size
2003	September–October	Kokanee reference	3+	130
2012	September–October	Sockeye reference	3+	148
2008	September–October	Annual trawl survey	0	96
2010	September–October	Annual trawl survey	0	96
2012	September–October	Annual trawl survey	0	96
2014	September–October	Annual trawl survey	0	96
2013	September–October	Annual trawl survey	1–2	136
2015	September–October	Angler survey	2–5	45

To assess the changes in the genetic structure of the *O. nerka* populations in Skaha Lake over time and the level of introgression between stocks, we obtained annual trawl survey (ATS) tissue samples during September to October in 2008, 2010, 2012, and 2014 from the Okanagan Nation Alliance. These annual trawls were performed using a 3 m × 7 m mid‐water trawl designed by Enzenhofer and Hume (1989). The net was towed at night, at up to six depth strata, and surveys were based on four to eight trawls per sampling session. After each trawl set, fish were immediately removed from the net and held on ice. At the laboratory, all fish were assigned a unique fish identification number and were processed for lengths (millimeters) and weights (grams). Otoliths were removed and placed in dry vials, and the whole fish was placed in vials of ethanol.

September/October samples were chosen for three reasons: (i) to ensure the temporal consistency between years; (ii) to ensure that the smaller age‐0 kokanee were likely to be fully detectable in the netting; and (iii) to give a narrow age bracket, enabling morphological comparisons to be made.

A size‐stratified sample of 96 age‐0 wild‐spawned fish from each year were selected for genotyping. We assessed both age and origin (wild or hatchery) using otolith sectioning, with age determined through standard otolith annuli aging and origin determined by the presence or absence of a thermal mark; thermal marking is performed in the hatchery to enable the identification of hatchery‐raised fish. We size‐stratified by ranking fish without thermal marks in each year from largest to smallest, then evenly sampling across this range.

Along with the biyearly sample of age‐0 fish, we also included samples from (i) all available age‐1 and age‐2 fish from the ATS trawls caught over the fall of 2014 (*n* = 136) and (ii) all available samples from angler surveys (age 2–5 fish caught and retained; *n* = 44) conducted at the boat launch on Skaha Lake in 2015 by BC MFLNRO personnel. This angler survey is conducted from April to August before the sockeye return, and thus, only resident fish are sampled. These older individuals were genotyped in order to assess whether the hybrid or pure anadromous sockeye remained in the lake beyond their usual migration out to sea.

### 2.2 DNA extraction, genotyping, and panel development

DNA was extracted using a standard Chelex‐based protocol (Walsh, Metzger, & Higuchi, 1991). Extractions were conducted in 200 μl volumes consisting of 195 μl 10% Chelex solution and 5 μl proteinase K (10 mg/ml), and incubated for 2 hrs at 55°C and then 95°C for 15 min in an Applied Biosystems Veriti™ thermal cycler (Applied Biosystems, Foster City, CA, USA).

Single nucleotide polymorphism genotyping was performed using TaqMan™ assays in 6 μl reaction volumes: 2.5 μl TaqMan™ Universal PCR Master Mix (Life Technologies, Carlsbad, CA, USA), 0.25 μl TaqMan™ Genotyping Assay (20×), 1.25 μl H_2_O and 2 μl of 1/10 diluted extracted DNA. Genotyping reactions were performed in 384‐well plates using an Applied Biosystems ViiA7™ Real‐Time PCR system (Life Technologies).

The sockeye and kokanee reference samples were genotyped at an initial set of 53 SNPs that included 40 previously published TaqMan™ assays by Elfstrom, Smith, and Seeb (2006), Campbell and Narum (2011), and Dann, Jasper, Hoyt, Hildebrand, and Habicht (2012) (Table S1). In addition, we tested 13 newly designed TaqMan™ assays targeting outlier loci identified from Lemay and Russello (2015) and Veale and Russello (in preparation) (Table S1). For these two reference datasets, we calculated pairwise *G*_ST_ values between kokanee and sockeye for each locus in GENALEX 6.501 (Peakall & Smouse, 2012). We then created an optimized marker panel including a subset of these markers (*n* = 35) using backwards elimination to determine the best combination of loci to differentiate between groups as performed in BELS (Bromaghin, 2008) using a mixture sample size of 200 per population and 250 replicates (see 3 for further details). All remaining samples (trawl and angler‐caught samples) were then genotyped at this optimized marker panel.

### 2.3 Population structure and hybridization analysis

To assess the population genetic structure within our reference samples of Okanagan River sockeye and Skaha Lake kokanee, we used STRUCTURE 2.3.4 (Pritchard, Stephens, & Donnelly, 2000) using the correlated allele frequency model allowing admixture, without location prior, and with a burn‐in period of 1,000,000 followed by 1,000,000 iterations. Runs were conducted varying the number of clusters (*K*) from 1 to 4 with 10 iterations at each value of *K* and implementing the Δ*K* approach (Evanno, Regnaut, & Goudet, 2005) in STRUCTURE HARVESTER (Earl & von Holdt, 2012).

We used three methods to assign stock and quantify the level of introgression for each sampled individual. First, we used the approach implemented in NEWHYBRIDS (Anderson & Thompson, 2002) that computes the posterior probability of various introgression classes for each individual. NEWHYBRIDS was run using 10,000 burn‐in and 50,000 iterations postburn‐in with a potential ancestry matrix that included kokanee, sockeye, F1 hybrids, F2 hybrids, kokanee backcrosses, and sockeye backcrosses, and also with a reduced set that only included the two pure stocks (kokanee and sockeye) and F1 hybrids. We conducted the latter analysis to assess whether a simplified ancestry matrix would increase the assignment accuracy. In both cases, the individuals were assigned to the class that maximized the probability of assignment. Second, we used the prior of Rannala and Mountain (1997) to optimally assign individuals to one of three simulated populations (pure kokanee, pure sockeye, and F1 hybrids, *n* = 600 for each) as implemented in GENECLASS2 (Piry et al., 2004). These simulated populations were based on the empirical genotypic data obtained from the two reference populations and created using the program HYBRIDLAB (Nielsen, Bach, & Kotlicki, 2006). As above, individuals were assigned to the class that maximized the probability of assignment. Third, we used the membership coefficients resulting from our STRUCTURE analysis (*K* = 2) and empirically derived assignment criteria (sockeye < 0.21, hybrid 0.21–0.81, kokanee > 0.81) using simulated datasets as described below.

To conduct a quantitative assessment of the STRUCTURE analysis, we used simulated hybrid classes created in HYBRIDLAB. We created five replicated simulated data sets, each including 600 kokanee, 600 sockeye, 100 F1 hybrids, 100 F2 hybrids, 100 kokanee backcrosses, and 100 sockeye backcrosses. The larger size of the two pure stocks was chosen because clustering and assignment is more accurate when pure stocks are well represented in the data and because this more likely reflects the composition of our data. The latter was revealed by preliminary NEWHYBRID and STRUCTURE analyses while also balancing the need to have a reasonable sample size of hybrids to inform the confusion matrixes. We then analyzed these simulated populations in STRUCTURE using the same parameters as described earlier. Based on the results from these analyses, we determined the cluster membership (kokanee or sockeye) kernel density distributions using Gaussian smoothing for each ancestry class (500 individuals per class) and plotted these distributions using ggplot2 (Wickham, 2009) in R (R Core Team 2016). From these distributions, we then determined the optimal cluster membership ranges for defining each ancestry class by strict maximum likelihood, so that for a given cluster membership proportion, the most likely ancestry class is given.

To assess the power of each of these three analyses, we then analyzed the five simulated datasets using the three approaches described above as implemented in NEWHYBRIDS, STRUCTURE, and GENECLASS2 with the same parameters as for our observed data. From these analyses, we then created confusion matrices to show the level of correct self‐assignment to ancestry class, and the rates of misassignment into each of the other classes. Each confusion matrix was created with a strict maximum‐likelihood criterion—each individual was assigned to the most likely ancestry class from the options, even if there were multiple classes with similar probabilities.

### 2.4 Population morphometric comparison

After classifying each individual as either pure stock (kokanee or sockeye) or hybrid, we compared the length and weight distributions for each of these classes using an ANOVA, performed in R (R Core Team 2016). We also displayed these distributions graphically using ggplot2 (Wickham, 2009).

## 3 Results

The initial set of 53 markers had an assignment accuracy of 99.99% as calculated in BELS (Bromaghin, 2008), and assignment accuracy did not decrease below 99.99% using the reduced 35 loci marker set. This reduced marker set excluded loci that added no increase in power, and had a pairwise *G*_ST_ of <0.01 between kokanee and sockeye. The final dataset was comprised of 844 individuals genotyped at 35 SNPs, including the reference Okanagan River sockeye and Skaha Lake kokanee spawners (prehatchery restocking), annual trawl survey samples in 2008, 2010, 2012, 2014, and angler‐caught samples in 2015 (Table 1). The amount of missing data in the final dataset was 0.7% across all loci, with a maximum of 1.5% missing data per locus.

**Figure 2:**
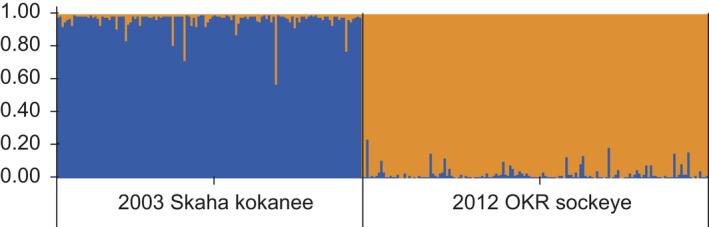
STRUCTURE analysis showing the proportional cluster membership of each *Oncorhynchus nerka* individual from the two reference populations. Columns represent individuals, and the proportion of each of the two colors represents the proportion of cluster membership. Blue represents the “kokanee cluster”; orange, the “sockeye cluster”

Differentiation between the reference kokanee and sockeye populations was moderate (*G*_ST_ = 0.14), with pairwise *G*_ST_ ranging from 0.01 to 0.60 (Figure S1). The STRUCTURE analysis revealed *K *= 2 as the most likely number of clusters (Δ*K*_2_ = 1410.99), corresponding well with the two reference populations (kokanee and sockeye; Figure 2). Given the differentiation between stocks and the lack of any secondary structure, we then proceeded to analyze the patterns of hybridization and introgression within our data.

**Figure 3:**
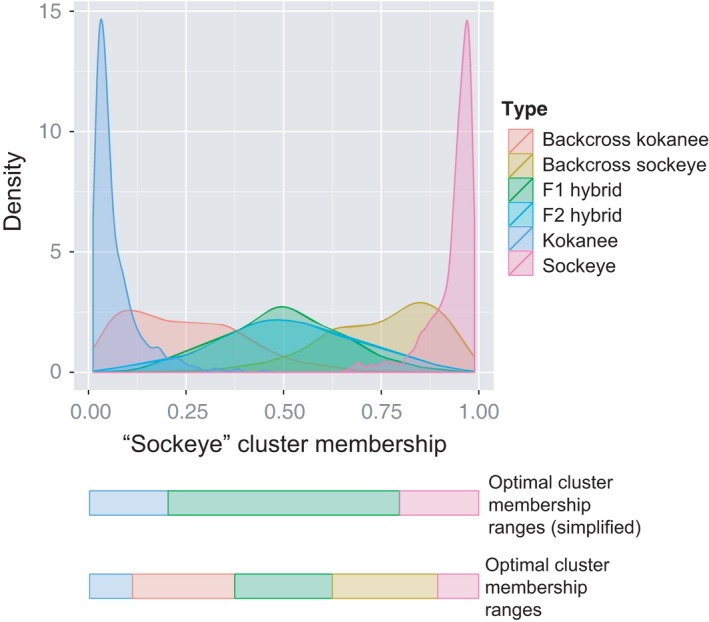
STRUCTURE cluster membership distributions for each introgression class based on the complete 35 SNP dataset, showing the optimal ranges for assigning each class

For the STRUCTURE analysis of the simulated datasets, we found that there was a considerable overlap between introgression classes for cluster membership when all possible introgression classes were included (Figure 3). When we limited introgression classes to just the two pure stocks and F1 hybrids, overlap became minimal (Figure 3). Using these simplified introgression classes, assignment accuracy was 95% or greater across classes (Table 2d).

**Table 2:** Confusion matrices showing the proportional assignment to introgression class for the simulated data for NEWHYBRIDS, STRUCTURE, and GENECLASS2

(A) NEWHYBRIDS						
	Kokanee	Backcross kokanee	F1 hybrid	Backcross sockeye	Sockeye	F2 hybrid
Kokanee	0.96	0.03	0.00	0.00	0.00	0.01
Backcross kokanee	0.37	0.43	0.12	0.03	0.00	0.05
F1 hybrid	0.00	0.02	0.66	0.16	0.00	0.16
Backcross sockeye	0.00	0.00	0.15	0.41	0.34	0.10
Sockeye	0.00	0.00	0.00	0.06	0.94	0.00
F2 hybrid	0.08	0.07	0.26	0.16	0.07	0.36

(B) STRUCTURE						
	Kokanee	Backcross kokanee	F1 hybrid	Backcross sockeye	Sockeye	
Kokanee	0.84	0.15	0.00	0.00	0.00	
Backcross kokanee	0.24	0.58	0.17	0.01	0.00	
F1 hybrid	0.00	0.22	0.56	0.21	0.01	
Backcross sockeye	0.00	0.02	0.17	0.61	0.20	
Sockeye	0.00	0.00	0.00	0.16	0.84	

(C) NEWHYBRIDS						
	Kokanee	F1 hybrid	Sockeye			
Kokanee	0.99	0.01	0.00			
F1 hybrid	0.03	0.94	0.03			
Sockeye	0.00	0.01	0.99			

(D) STRUCTURE						
	Kokanee	F1 hybrid	Sockeye			
Kokanee	0.96	0.04	0.00			
F1 hybrid	0.03	0.95	0.02			
Sockeye	0.00	0.05	0.95			

(E) GENECLASS2						
	Kokanee	F1 hybrid	Sockeye			
Kokanee	0.95	0.05	0.00			
Sockeye	0.00	0.04	0.96			
F1 hybrid	0.02	0.95	0.03			

Rows show true introgression class, and columns are the assigned class. A & B show confusion matrices for the complete ancestry classes; C, D & E show the simplified ancestry classes.

The NEWHYBRID analysis on the simulated data showed similar levels of assignment accuracy to the STRUCTURE analysis, with limited assignment accuracy when using all introgression classes. Self‐assignment accuracy substantially improved using the simplified introgression class matrix, with very high accuracy for both pure stocks (>99%) and good assignment accuracy for F1 hybrids (94%; Table 2c).

The GENECLASS2 assignment of the simulated data showed similar accuracy to the other two methods, with at least 95% accuracy for self‐assignment for any given introgression class (Table 2e).

There was a high level of agreement between the three methods to assess introgression within the empirical datasets (Table S2). Across all three analyses, a small number of hybrids were indicated in the reference data, with NEWHYBRIDS and STRUCTURE having low detected hybrid numbers (1.8% and 1.4%, respectively), while GENECLASS2 had a higher rate (6.8%). Given the error rates indicated by the simulated data analysis, these supposed hybrid individuals observed in the reference populations are conceivably false positives.

**Figure 4:**
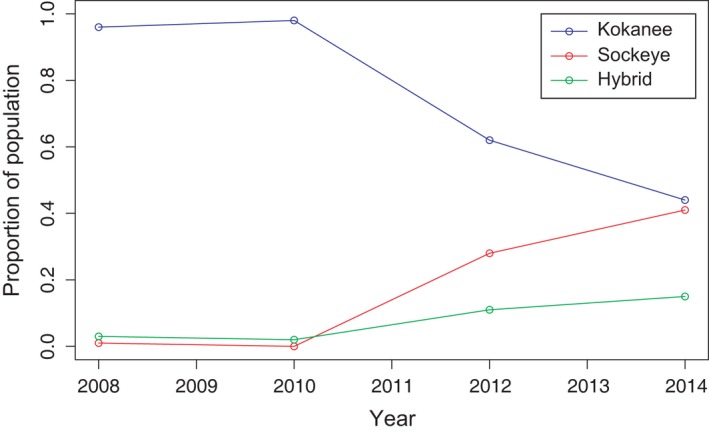
Proportion of pure and hybrid juvenile (age‐0) *Oncorhynchus nerka* in the Skaha Lake population over time

During 2008 and 2010, there were low numbers of both sockeye and hybrids detected within the age‐0 trawl samples—one sockeye detected in 2008 and five hybrids detected across the two years (Figure 4; Table S2). Then in 2012, 28% of the age‐0 trawl samples were sockeye juveniles, rising to 41% in 2014 (Figure 4; Table S2). The number of hybrids detected in these two years rose proportionally with the increase in sockeye juveniles, with 11% and 15%, respectively (Figure 4).

Interestingly, there was one pure sockeye juvenile detected among the age‐1 fish sampled in 2013. Moreover, hybrid individuals were still detected in the age‐1 and age‐2 cohorts sampled and particularly in the older angler‐caught samples from 2015 (Table 3). The 2015 angler‐caught samples had the highest proportion of hybrids detected among all populations sampled. These samples largely correspond to the age‐0 cohort sampled in 2012, and while there was a proportional increase in hybrids compared with kokanee from 2012 to 2015, this increase was not significant (χ^2^ = 1.67, *p* = .19).

**Table 3:** Proportion of each introgression class for each sampled population

Year	Type	Kokanee	Sockeye	Hybrid
2008	Age 0	0.96	0.01	0.03
2010	Age 0	0.98	0.00	0.02
2012	Age 0	0.62	0.28	0.11
2014	Age 0	0.44	0.41	0.15
2013	Ages 1–2	0.91	0.01	0.08
2015	Ages 2–5	0.76	0.00	0.24

Results defined using a majority rule: a minimum of two‐thirds of the analyses assigned each individual to each introgression class (see Table S2 for detailed results).

**Figure 5:**
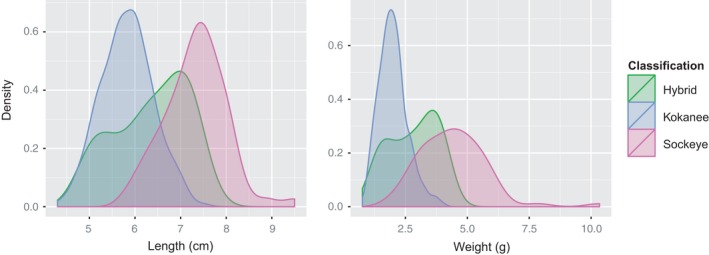
Length and weight distributions for age‐0 *Oncorhynchus nerka* caught in Skaha Lake during October 2008–2014

Hybrid age‐0 individuals had an intermediate fork length and weight compared to the smaller kokanee and larger sockeye (Figure 5). This difference in size between the three types was highly significant for both length (*F* = 328.7, *df* = 368, *p* = 2.2e‐16) and weight (*F* = 478.2, *df* = 368, *p* = 2.0e‐16), with highly significant differentiation between even the closest two types (kokanee and hybrid; length: *F* = 20.3, *df* = 301, *p* = 9.51e‐0; weight: *F* = 44.61 *df* = 301 *p* = 1.16e‐10). We detected no significant differences between kokanee, hybrids, and sockeye for the higher age classes, which may be due to the extremely small sample sizes observed for sockeye and hybrids present in these age classes.

## 4 Discussion

### 4.1 Kokanee–sockeye hybridization in Skaha Lake

Our results demonstrate that (i) the reintroduction of wild‐spawning anadromous sockeye salmon into Skaha Lake has succeeded, with large numbers of natural recruits now originating from this population, and (ii) hybridization is occurring between the reintroduced sockeye salmon and the resident kokanee in Skaha Lake, with some of these hybrids retained within the lake to maturity. Moreover, there are occasional pure sockeye retained in the lake beyond their normal migration cycle.

The unintended introgression of hatchery stock within other resident populations has occurred as the result of other Pacific salmon translocations (Bartley, Gall, & Bentley, 1990; Hess et al., 2011; Spies et al., 2007; Utter, 2000). In some instances, when this introgression was between populations with divergent life histories, outbreeding depression has been demonstrated (Gharrett, Smoker, Reisenbichler, & Taylor, 1999; Gilk et al., 2004). The potential for negative fitness impacts (outbreeding depression) in situations of intraspecific introgression is, however, difficult to predict (McClelland & Naish, 2007).

In our study, while we found significant levels of hybridization between kokanee and reintroduced sockeye, the relative fitness of these hybrids remains uncertain. Previous studies have indicated that fitness of kokanee–sockeye hybrids may be reduced compared with pure forms, with hybrids showing differing development rates (Wood & Foote, 1990), ontogeny to seawater (Danner, 1994; Foote et al., 1992), and growth and onset of maturity (Wood & Foote, 1996). In addition to the potential for reduced fitness due to different environmental selection pressures, hybrids may also have reduced fitness due to sexual selection. Both kokanee and sockeye have a strong sexual preference for the color red during spawning (Foote, Brown, & Hawryshyn, 2004), but kokanee (which inhabit carotenoid‐poor lakes) are more efficient at acquiring and storing these pigments than sockeye (Craig & Foote, 2001). The offspring of sockeye that remain in freshwater develop far less attractive green coloration, as do kokanee–sockeye crosses (Craig & Foote, 2001; Craig et al., 2005). Although we detected strong evidence of hybridization and introgression between sockeye and kokanee, future studies quantifying hybrid fitness are required to explicitly evaluate the evolutionary consequences, if any, of this ongoing repatriation program.

If the current rates of sockeye return remain constant, then demographic swamping of the Skaha Lake kokanee, nevertheless, seems unlikely. Given that the observed hybridization in other lake systems has generally been kokanee males sneaking on spawning sockeye pairs (Foote & Larkin, 1988; Foote et al., 1997), and reproductive output of the population is more limited by females than by males, the effects of this hybridization will be biased toward decreasing sockeye rather than kokanee productivity.

Genetic swamping still remains a possibility, although we cannot yet assess what the ongoing genetic effects of the sockeye reintroduction will be on resident kokanee. Because anadromous sockeye salmon historically spawned in Skaha Lake and were only excluded within the last century, it is likely that some hybridization occurred prior to their isolation. Introgression between kokanee and sockeye is generally limited by (i) assortative mating based on size (Foote & Larkin, 1988); (ii) relative numbers of kokanee to sockeye; (iii) spatial segregation of spawning, as kokanee females dig nests in areas of lower water velocity with finer grain size, and access smaller sections of streams than female sockeye (Wood & Foote, 1996); and (iv) temporal segregation of spawning period (Dodson et al., 2013), although in Skaha Lake, spawning periods significantly overlap.

Despite these isolating mechanisms, we observed high rates of hybridization now taking place post‐reintroduction, and this leads to the question: “Have anthropogenic changes in Skaha Lake led to an increased rate of hybridization?” Without historical samples, we are unable to answer this question; however, this remains a possibility. While sockeye did historically migrate into Skaha Lake, the numbers and consistency of this migration are unknown. It is difficult to assess the historical levels of hybridization in this system; however, Skaha Lake kokanee are significantly more closely related to neighboring kokanee populations (Okanagan Lake kokanee *F*_ST_ = 0.03, and Wood Lake stream spawning kokanee *F*_ST_ = 0.06) than they are to Okanagan River sockeye (*F*_ST_ = 0.10) (Veale & Russello in prep); therefore, either introgression was low, or traces of this introgression have largely been lost (or selected against) since isolation. It is possible that the numbers returning were historically lower or inconsistent due to the difficulties passing Okanagan Falls; therefore, the large numbers now returning could be artificially high. Second, it is also possible that spawning habitat has become more restricted or less varied due to anthropogenic changes in the channelization and degradation of streams, limiting the spatial segregation of spawning, with kokanee and sockeye now potentially forced into the same areas.

Of particular note was the large proportion of hybrids within the angler‐caught samples. This may indicate that anglers preferentially keep hybrids due to their larger size. This increase in large kokanee in the lake is probably seen as a positive benefit for recreational fishermen. If this fishing preference is large enough, it could help prevent higher levels of introgression by removing hybrids from the population; however, the level of angler pressure needed to achieve this end is unknown.

### 4.2 Detecting hybrids

Detecting the level of hybridization for individuals, particularly from populations that are genetically similar, remains challenging (Hess et al., 2011). Here, we used a range of methods in order to improve confidence in addressing study questions. In particular, we stress the importance of simulating hybridization classes to allow for the estimation of error rates for implemented assignment approaches. In our case, all methods employed yielded largely congruent assignments as either pure stock or hybrid with high confidence for each individual using our SNP panel. While there were a small number of individuals with marginal assignments or some discrepancies between methods, we retained these individuals as removing them would disproportionately remove hybrids (as this is the smallest group and the one most likely to have marginal assignment). We acknowledge that there will be some uncertainty in individual assignment, but feel that the overall population trend is as accurate as possible and that using multiple methods maximizes robustness of the assignments. There were insufficiently high error rates for assigning individuals to more complex hybrid classes (e.g., F2, backcrosses, or higher). If it is not possible to discriminate between such hybrid classes, additional markers will be required, or the use of simplified hybrid classes will be necessary. The resolution required to identify more complex hybrid classes increases exponentially; therefore, researchers will need to account for this depending upon the questions being addressed. For our purposes, simplified hybridization classes were adequate, and we deemed the chances of higher‐level hybridization to be low, given the timeframes since reintroduction. We also feel that applying a more quantitative method with STRUCTURE outputs (as carried out here) is fundamental for evaluating performance of a marker set for stock assignment while providing greater interpretability and resolution than conventional qualitative descriptions.

The observation that the length and weight distributions detected for hybrids were intermediate between those for sockeye and kokanee complements previous research, indicating that hybrids are generally an intermediate phenotype (Wood & Foote, 1996), and also highlights the accuracy of our hybrid detection methods. While we observed some morphological differentiation between kokanee, sockeye, and hybrids even at this juvenile stage, the differences are not enough to assign individuals into each of these classes; therefore, genetic measures remain necessary for monitoring these populations.

### 4.3 Management implications

Our study revealed that the reintroduction program for anadromous sockeye salmon to Skaha Lake is succeeding, with large numbers of fish now returning to the lake to spawn. If this trend continues, hatchery production of fry could potentially be reduced over time as the Skaha Lake sockeye population becomes self‐sustaining. However, the level of hybridization with native kokanee is of potential concern and may be indicative of reduced spawning habitat area and diversity. Decreasing human intervention and allowing these populations to re‐establish natural reproductive isolation may be a viable solution, as has been pursued for other species (McLean, Bentzen, & Quinn, 2004). Alternatively, creating spawning channels of various sizes and characters may be useful in preventing high levels of introgression; already, the Okanagan Nation Alliance has installed several spawning platforms with gravel sizes more suitable for kokanee. There is also the option for preventing sockeye passage into Skaha Lake during years where it is believed that the kokanee spawning run will be low in order to minimize hybridization or competitive interactions. Ongoing genetic monitoring of these populations is advised, enabling the assessment of the long‐term effects of introgression and the integration of scientific information to support interactive fisheries management. Moreover, the framework demonstrated here should have broader utility for other sockeye repatriation programs, especially when integrated into the planning stages of such initiatives to inform risk assessment and the subsequent monitoring of potential impacts to native stocks.

## Data Archiving Statement

Data for this study have been deposited in the Dryad Digital Repository under the following DOI: http://dx.doi.org/10.5061/dryad.n720d.
