# Supplementary material for: Sockeye salmon repatriation leads to population re‐establishment and rapid introgression with native kokanee
Source: Evol Appl. 2016 Oct 21;9(10):1301–11. doi: 10.1111/eva.12430 (PMC5108220; doi:10.1111/eva.12430)
Supplement: Supplementary file 1 [file EVA-9-1301-s001.docx]

# Sockeye salmon repatriation leads to population re-establishment and rapid introgression with native kokanee

# Andrew J Veale^#^ and Michael A Russello^*^

Department of Biology, The University of British Columbia, Okanagan Campus, 3247 University Way, Kelowna, British Columbia, Canada V1V 1V7

**Supplementary Figures and Tables**

Figure S1. Pairwise G_ST_ values between kokanee and sockeye for each of the loci retained in the study. Additional outlier loci indicated in red, original loci in blue.

Table S1. Taqman assays used for SNP genotyping.

| Assay Name | VIC | FAM | VIC Probe Sequence | FAM Probe Sequence | Forward Primer Sequence | Reverse Primer Sequence | Source | Retained |
| --- | --- | --- | --- | --- | --- | --- | --- | --- |
| One_aldB-152 | A | G | CTCAGGCATTACCTTC | CAGGCATCACCTTC | CGATCAGGTGACGCTAAAATTAACTC | GTGGCTTCCTCTTCACTCTGA | Campbell & Narum (2011) | Yes |
| One_cin-177 | C | T | TCACGCACGGGACAG | CACGCACGGAACAG | CCTCAGACTAGTGACCGTACCTA | CGCTCACCGTGGTTACGT | Campbell & Narum (2011) | Yes |
| One_gdh-212 | C | A | ATCTGTTACCAGAATGTTT | ATCTGTTACCATAATGTTT | CCTGTGTTGAAGTGGAGTAGGTTAA | GCTTTATACTGTAAGTGGACTGACCTT | Campbell & Narum (2011) | No |
| One_metA-253 | C | G | AGGCAATTGAGGTTAAT | AGGCAATTGACGTTAAT | TTCTTATCGCTGGTGGCACTTT | GACCAAAGACTATTTAGTTGCCACCTA | Campbell & Narum (2011) | Yes |
| One_Ots208-234 | - | A | CACACGTTACATCAGATAACT | CACACAATGTTACATCAGATAAC | CAGCCGACATGCATCAGTTA | TGACCCCATGTTTCATGCT | Campbell & Narum (2011) | Yes |
| One_redd1-414 | T | C | CCTAAGTCAGTCACTGTAG | CCCAAGTCAGTCACTGTA | GTTGGCTACATCCTAAAACACAATGG | CAGCCCTGGAGTACTGAATCAG | Campbell & Narum (2011) | No |
| One_sast-211 | G | T | CATCATTTGCATTATTG | CATCATTTGAATTATTG | TGTACTTAGTCCAATAAGCATTTCAACAGT | TGGCTAGATTCACATGGTCAACAAA | Campbell & Narum (2011) | Yes |
| One_SUMO1-6 | C | A | CAAGATTGAAATTGGTTTGC | CAAGATTGAAATTTGTTTGC | GCACAAGCCAAAAAGTTTTCTCCAT | GGACATAGTTGGAGGCAGACAAAA | Campbell & Narum (2011) | No |
| One_tshB-92 | A | C | ACCACCCTGTAGCTCA | CACCCTGGAGCTCA | GCATTGTCGTACTCGTGTGTTTG | CACAACAGCAACAATACATGTCACA | Campbell & Narum (2011) | Yes |
| One_txnip-401 | C | T | TGACTGCACTAGTTTAGAC | TGACTGCACTAATTTAGAC | GCCAGATCCCTTCAGTTGGA | GGCCATTTCAAAAGGCTGCAT | Campbell & Narum (2011) | Yes |
| One_agt-132 | A | C | ACAGGAAAATCACGAGCCT | CAGGAAAATCCCGAGCCT | GACCCAGATCAACAACTTCATCCA | TGGTTGAGCTAAGGTCCTTGAAC | Dann et al. (2012) | No |
| One_cetn1-167 | A | C | TTGACGAAGCAGACCGA | TTGACGAAGCCGACCGA | CAGAAATCCTGACTGTTAAAACAATGCA | CTGCTCGTTGATCTCTCCATCTC | Dann et al. (2012) | No |
| One_GPDH-201 | T | C | CTTCACCCCTGGAGCC | CACCCCCGGAGCC | GAAGCTGATCCTAGACCTGTACCTA | TGGTATGATGGTGCTACTGGAAGT | Dann et al. (2012) | No |
| One_HGFA-49 | A | T | CTAAAGCACCATGTTGC | ACTAAAGCACCTTGTTGC | ACTTGCTACTTCAGGGTTTTTGTGA | TGGCAGAACAATTCCTCAATGCATA | Dann et al. (2012) | Yes |
| One_Hsp47 | A | G | TTATTGACTATGGCACATTG | TTGACTATGGCGCATTG | CGTTCAAATAAATGCTGTTTGGCCTTT | GTGGTGTTCGGATTTTTCCTGAAA | Dann et al. (2012) | Yes |
| One_PIP | C | T | AACACACATTTCTCAACACA | ACACACATTTTTCAACACA | ACAGAGTCAGGACTTGATATGTACAGA | CCTGACGAGGGTCTACTACACT | Dann et al. (2012) | No |
| One_rab1a-76 | G | T | TGTGGAGCAAGGTAACT | TGTGGAGCAATGTAACT | TCGCCATATTCTCTCTCCCTATCC | ATCCACTCAGACCCATATCTACCAA | Dann et al. (2012) | Yes |
| One_RFC2-102 | A | G | ATCACGTTGTATTTCTTT | CACGTTGTGTTTCTTT | TCCAGGAGCTGCATTTTGAGTTAAA | AAGGTGGATGACAATGTGTTAGTGT | Dann et al. (2012) | Yes |
| One_U1009-91 | A | G | CATGTTCTGTATGGACCC | TGTTCTGTGTGGACCC | CTCTGTCCTTGAACTGTTGTCTGTT | GCCGCTGCTACTCTTCCT | Dann et al. (2012) | No |
| One_U1012-68 | C | T | TGACGGGTGTTCCTGATAA | TGACGGGTGTTCTTGATAA | TCTATTACCATACAGGCCCAGTACA | CCTTTTGTGTCTTCCAGTCATGTGA | Dann et al. (2012) | Yes |
| One_U1024-197 | G | T | ACCTGACCCAACAAA | ACCTGACACAACAAA | CTGAACTGATCTACCGCTCTGT | GGAACAGATACTCCAGGAGAGATGA | Dann et al. (2012) | Yes |
| One_U1101 | C | A | TGGACGTATGTCATATTT | TGGACGTATGTAATATTT | CTATGACATGTTTATTTTAATTAGCCACCAACT | AGTATAGCTAGGGAACCTTTCGATCTT | Dann et al. (2012) | No |
| One_U1105 | T | A | CCTGTTTTTTTTAAAAGAC | TCCTGTTTTTTTTTAAAGAC | GCCTTAATAGTGTCTTCTGATCCCTTT | CCCTCTGTTGTCCAGACTCTTAG | Dann et al. (2012) | Yes |
| One_U1201-492 | A | G | AAGACTTCCTCCAGGCTC | ACTTCCCCCAGGCTC | GCTTATGACGGAGAAGAGATGCA | AGGATACTGAAGCCCAGAGACA | Dann et al. (2012) | No |
| One_U1206-108 | G | T | AACATTGAGCTTCCC | ATAACATTGATCTTCCC | CTGAGATGGTGCTTTCTGAGGATA | TGGATGAAAGGGAAATTCTGTCAACA | Dann et al. (2012) | Yes |
| One_U1208-67 | A | C | CCCAATGTGATTGTCAC | CCAATGTGCTTGTCAC | ACTTGAATGTCTGTTTCGTAGGTGAT | ACACAGTTGACAGTGGAGCAA | Dann et al. (2012) | Yes |
| One_U1212-106 | A | G | TTTTGACATACAAAAAATA | TTTGACATACAGAAAATA | CGTAATGACCTACCACCATATCAGT | TGGCATGACTTTAACAATTCCCAAAAAA | Dann et al. (2012) | Yes |
| One_U1216-230 | A | T | CCTGGCTACTAAGTAAC | CTGGCTACAAAGTAAC | TGGGATCGGACGTCAATAGATTTC | GTAATACAGAGTGAGCGTGATACATTGT | Dann et al. (2012) | No |
| One_GHII-2165 | T | A | CACAAATGGAAATTGA | CACAAATGGTAATTGA | GGCATCAACCTGCTCATCGA | TGCACAAAGTGCGGCAC | Elfstrom et al. (2006) | Yes |
| One_HpaI-71 | A | T | TCAGTTAAGAACTAATTCT | AGTTAAGAACAAATTCT | TGTTGTTCCTAGGCTGTCATTGAAA | CCCTGCGTATTACTAAGGCCATATTTATT | Elfstrom et al. (2006) | Yes |
| One_HpaI-99 | C | T | AACGGAAGAAACCCCTCAA | AACGGAAGAAACTCCTCAA | CCTGAGTTGTGTTCAATGGGCATAA | TGGGTCATGTTCATTAGAGCACAAA | Elfstrom et al. (2006) | Yes |
| One_KPNA-422 | A | G | CTGGTATGAGAAGGCACA | TGGTATGAGGAGGCACA | TGGGCCCTGGGAAACATC | CCATAGCCACTTTCGATACAGGTAA | Elfstrom et al. (2006) | Yes |
| One_LEI-87 | A | G | ACTCGCCACCTCTGT | TCGCCGCCTCTGT | ACAGCGCATCCCCATAATGG | GCCTTTGTGGAGGTCAACGA | Elfstrom et al. (2006) | No |
| One_MHC2_190 | G | T | CTGCTATCGACTACAGC | CGCTGCTATCTACTACAG | GTATGGTGTGAAGAATGCA | GCTCACCTGTCTTGTCCAGTA | Dann et al. (2012) | Yes |
| One_MHC2_251 | C | T | CACTTACAGGCCCCTG | CACTTACAGGCCTCTG | CTGGACAAGACAGGTGAGCA | AAAGTAATGGTCTTGACTTGATCA | Dann et al. (2012) | No |
| One_Ots213-181 | T | A | CTTTGAATTAAAAACATTTTT | CTTTGAATTAAAAACTTTTTT | CCATAGTGTATCACACAATACTCATGTCT | TCTATCATCTGCAAATCTGTGTACTAGACT | Elfstrom et al. (2006) | No |
| One_p53-534 | C | A | ATGTCCAAAGATCTGG | AATGTCCAAATATCTGG | GACAATCTTAAAGCGGTGGTCTTG | AACCTTTATCAGCCATCATCCAACT | Elfstrom et al. (2006) | Yes |
| One_Prl2 | G | T | ACCAATGGGACGAGTG | CCACCAATTGGACGAG | ACCTCTCTCTCTCTCAGGACTCTCA | GAGGAGGTGTGACACATAGATGGA | Elfstrom et al. (2006) | No |
| One_STC-410 | T | C | CCGATGGGTATATTATTATA | CCGATGGGTATATTGTTATA | CAACACAACATCAACATCATTAATAAACATTCTG | AACATCCCCGTTTTGACCACTTAT | Elfstrom et al. (2006) | Yes |
| One_STR07 | G | C | ACGCACACTGTCCTT | ACGCACACTCTCCTT | CACACCTGAGGCACAAGCT | GTATGTCTACCAGAGAGGTCAAGGA | Elfstrom et al. (2006) | No |
| One_108521 | A | G | CAGCTATGAAATCAGC | AGCTATGAAATCGGC | GAATTTCATTACATTCCAGGGTTAGATTGATT | TGCAGGTCCTGATCTTTTGGAT | Lemay & Russello (2014) | No |
| One_121265 | C | G | TCCATCACGCTGCTGC | CTCCATCACCCTGCTGC | GTATCTGAAGCTGTTCACCTTCCTA | TGCCTGGTTCCGGTCTCT | Lemay & Russello (2014) | Yes |
| One_13802 | G | A | TCCAGGAGAGGCTG | CTCCAGAAGAGGCTG | GCCCTCCTGCACAAGCA | GTGGTCATCTCCTTCTCCTTGTAG | Lemay & Russello (2014) | Yes |
| One_29236 | A | C | CTCCCTCTTTTCCCCC | CCCTCTGTTCCCCC | CTGCAGGGAGAAGCTGTGTAG | GCGACTAGCCCTTGATGCT | Lemay & Russello (2014) | No |
| One_37568 | C | T | CATCTGACGAGGAAGAA | CATCTGACAAGGAAGAA | TCAAGCTCCTCCAGTGGTTC | CCTTGCACAGAGTTACATACATTTAAACAAT | Lemay & Russello (2014) | Yes |
| One_88470 | T | C | CCTTGGCATAACCCTG | CCTTGGCATGACCCTG | CCCTCCCAGAGTGCAAAGAA | CAGAGTACGTCAGGGTGTTGTC | Lemay & Russello (2014) | Yes |
| One_92099 | T | G | CACAATTTAACAAGCGTCG | CACAATTTAACCAGCGTCG | GTGACCAGGAGTCCCAAATGG | CTAGAGCACGATAAGCCAAGGAA | Lemay & Russello (2014) | Yes |
| One_74711 | T | G | TTTTCCCTCTTGTTATTGTT | CCCTCTTGGTATTGTT | CCAGAATGTTGTCGTTAGCGTTTTA | AGGGCCTAACACTTGAGCAAAA | Veale & Russello (in prep) | Yes |
| One_RACGAP1_7641 | A | G | AAGACTGTTGCTATAATGAG | ACTGTTGCTGTAATGAG | ACCATGGTATTTTTACTGCAAGTTAATTAAACC | AAACGCCAGCCCAAGGTAA | Veale & Russello (in prep) | Yes |
| One_DCSTAMP_47083 | A | G | CATCACAGAGCTCAGC | ATCACAGGGCTCAGC | GTGGTTCTCAAGAACGTCCAGAA | CCTCTTGGCCTGCAGGTT | Veale & Russello (in prep) | Yes |
| One_ANGPTL4_122749 | A | G | CTCTGCAAACATCAAAG | CTCTGCAAACGTCAAAG | CCTGCAGGAGCAAGTCACT | ACACCCTTCTCTGCAAACGTT | Veale & Russello (in prep) | Yes |
| One_HSP90_40949 | G | A | TGCAGGCTACGTCAATA | TGCAGGCTACATCAATA | ACCTCAGCACAAGATTAACAAAAGTCTTA | TGCACCACTATTAATATTTGAAGTATCTTTAGATTGT | Veale & Russello (in prep) | Yes |
| One_HGF_18232 | A | C | AGCAGGTGAAGATACAGG | AGCAGGTGAAGCTACAGG | GAGTAGCCAAAACATCATACGGAGAT | GCAGGTACGTGTGGTGCAT | Veale & Russello (in prep) | Yes |

Table S2. Consensus introgression classifications of all *O. nerka* individuals as derived from the three analysis programs. The most probable assignment category is highlighted for each analysis program according to the criteria described in the main text: Blue = Kokanee, Green = Hybrid, Pink = Sockeye.

| Sample Name | Year | Method | Type | NEWHYBRIDS | | |  | STRUCTURE | |  | GENECLASS2 | | |
| --- | --- | --- | --- | --- | --- | --- | --- | --- | --- | --- | --- | --- | --- |
|  |  |  |  | Kokanee | Sockeye | Hybrid |  | Kokanee | Sockeye |  | Kokanee | Sockeye | Hybrid |
| KO-OKC-03-002 | 2003 | Spawner | Kokanee | 1.00 | 0.00 | 0.00 |  | 0.98 | 0.02 |  | 0.99 | 0.00 | 0.01 |
| KO-OKC-03-003 | 2003 | Spawner | Kokanee | 1.00 | 0.00 | 0.00 |  | 0.99 | 0.01 |  | 0.99 | 0.00 | 0.01 |
| KO-OKC-03-004 | 2003 | Spawner | Kokanee | 1.00 | 0.00 | 0.00 |  | 0.92 | 0.08 |  | 0.79 | 0.00 | 0.21 |
| KO-OKC-03-005 | 2003 | Spawner | Kokanee | 1.00 | 0.00 | 0.00 |  | 0.95 | 0.05 |  | 0.92 | 0.00 | 0.08 |
| KO-OKC-03-006 | 2003 | Spawner | Kokanee | 1.00 | 0.00 | 0.00 |  | 0.97 | 0.03 |  | 0.96 | 0.00 | 0.04 |
| KO-OKC-03-007 | 2003 | Spawner | Kokanee | 1.00 | 0.00 | 0.00 |  | 0.98 | 0.03 |  | 0.99 | 0.00 | 0.01 |
| KO-OKC-03-008 | 2003 | Spawner | Kokanee | 0.97 | 0.00 | 0.03 |  | 0.94 | 0.07 |  | 0.72 | 0.00 | 0.28 |
| KO-OKC-03-009 | 2003 | Spawner | Kokanee | 1.00 | 0.00 | 0.00 |  | 0.99 | 0.01 |  | 1.00 | 0.00 | 0.00 |
| KO-OKC-03-010 | 2003 | Spawner | Kokanee | 1.00 | 0.00 | 0.00 |  | 0.99 | 0.01 |  | 0.99 | 0.00 | 0.01 |
| KO-OKC-03-011 | 2003 | Spawner | Kokanee | 1.00 | 0.00 | 0.00 |  | 0.99 | 0.01 |  | 1.00 | 0.00 | 0.00 |
| KO-OKC-03-012 | 2003 | Spawner | Kokanee | 1.00 | 0.00 | 0.00 |  | 0.99 | 0.01 |  | 1.00 | 0.00 | 0.00 |
| KO-OKC-03-013 | 2003 | Spawner | Kokanee | 1.00 | 0.00 | 0.00 |  | 0.99 | 0.01 |  | 1.00 | 0.00 | 0.00 |
| KO-OKC-03-014 | 2003 | Spawner | Kokanee | 1.00 | 0.00 | 0.00 |  | 0.99 | 0.01 |  | 1.00 | 0.00 | 0.00 |
| KO-OKC-03-015 | 2003 | Spawner | Kokanee | 1.00 | 0.00 | 0.00 |  | 0.99 | 0.02 |  | 0.98 | 0.00 | 0.02 |
| KO-OKC-03-016 | 2003 | Spawner | Kokanee | 1.00 | 0.00 | 0.00 |  | 0.99 | 0.01 |  | 1.00 | 0.00 | 0.00 |
| KO-OKC-03-017 | 2003 | Spawner | Kokanee | 1.00 | 0.00 | 0.00 |  | 0.98 | 0.02 |  | 1.00 | 0.00 | 0.00 |
| KO-OKC-03-018 | 2003 | Spawner | Kokanee | 1.00 | 0.00 | 0.00 |  | 0.99 | 0.01 |  | 1.00 | 0.00 | 0.00 |
| KO-OKC-03-019 | 2003 | Spawner | Kokanee | 1.00 | 0.00 | 0.00 |  | 0.98 | 0.02 |  | 0.99 | 0.00 | 0.01 |
| KO-OKC-03-020 | 2003 | Spawner | Kokanee | 0.98 | 0.00 | 0.02 |  | 0.93 | 0.07 |  | 0.51 | 0.00 | 0.49 |
| KO-OKC-03-021 | 2003 | Spawner | Kokanee | 1.00 | 0.00 | 0.00 |  | 0.99 | 0.01 |  | 1.00 | 0.00 | 0.00 |
| KO-OKC-03-022 | 2003 | Spawner | Kokanee | 1.00 | 0.00 | 0.00 |  | 0.98 | 0.02 |  | 0.99 | 0.00 | 0.01 |
| KO-OKC-03-023 | 2003 | Spawner | Kokanee | 1.00 | 0.00 | 0.00 |  | 0.98 | 0.02 |  | 0.99 | 0.00 | 0.01 |
| KO-OKC-03-024 | 2003 | Spawner | Kokanee | 0.99 | 0.00 | 0.01 |  | 0.97 | 0.03 |  | 0.95 | 0.00 | 0.05 |
| KO-OKC-03-025 | 2003 | Spawner | Kokanee | 1.00 | 0.00 | 0.00 |  | 0.99 | 0.01 |  | 1.00 | 0.00 | 0.00 |
| KO-OKC-03-026 | 2003 | Spawner | Kokanee | 1.00 | 0.00 | 0.00 |  | 0.99 | 0.01 |  | 1.00 | 0.00 | 0.00 |
| KO-OKC-03-027 | 2003 | Spawner | Kokanee | 0.97 | 0.00 | 0.03 |  | 0.91 | 0.09 |  | 0.43 | 0.00 | 0.57 |
| KO-OKC-03-028 | 2003 | Spawner | Kokanee | 1.00 | 0.00 | 0.00 |  | 0.99 | 0.01 |  | 1.00 | 0.00 | 0.00 |
| KO-OKC-03-029 | 2003 | Spawner | Kokanee | 1.00 | 0.00 | 0.00 |  | 0.99 | 0.01 |  | 1.00 | 0.00 | 0.00 |
| KO-OKC-03-030 | 2003 | Spawner | Kokanee | 1.00 | 0.00 | 0.00 |  | 0.99 | 0.01 |  | 1.00 | 0.00 | 0.00 |
| KO-OKC-03-031 | 2003 | Spawner | Kokanee | 0.97 | 0.00 | 0.03 |  | 0.84 | 0.16 |  | 0.29 | 0.00 | 0.71 |
| KO-OKC-03-032 | 2003 | Spawner | Kokanee | 0.99 | 0.00 | 0.01 |  | 0.94 | 0.06 |  | 0.87 | 0.00 | 0.13 |
| KO-OKC-03-033 | 2003 | Spawner | Kokanee | 1.00 | 0.00 | 0.00 |  | 0.95 | 0.05 |  | 0.97 | 0.00 | 0.03 |
| KO-OKC-03-034 | 2003 | Spawner | Kokanee | 1.00 | 0.00 | 0.00 |  | 0.99 | 0.01 |  | 1.00 | 0.00 | 0.00 |
| KO-OKC-03-035 | 2003 | Spawner | Kokanee | 1.00 | 0.00 | 0.00 |  | 0.98 | 0.02 |  | 0.95 | 0.00 | 0.05 |
| KO-OKC-03-036 | 2003 | Spawner | Kokanee | 1.00 | 0.00 | 0.00 |  | 0.99 | 0.01 |  | 1.00 | 0.00 | 0.00 |
| KO-OKC-03-037 | 2003 | Spawner | Kokanee | 0.98 | 0.00 | 0.02 |  | 0.93 | 0.07 |  | 0.59 | 0.00 | 0.41 |
| KO-OKC-03-038 | 2003 | Spawner | Kokanee | 1.00 | 0.00 | 0.00 |  | 0.99 | 0.01 |  | 1.00 | 0.00 | 0.00 |
| KO-OKC-03-039 | 2003 | Spawner | Kokanee | 1.00 | 0.00 | 0.00 |  | 0.99 | 0.01 |  | 1.00 | 0.00 | 0.00 |
| KO-OKC-03-040 | 2003 | Spawner | Kokanee | 1.00 | 0.00 | 0.00 |  | 0.99 | 0.01 |  | 0.99 | 0.00 | 0.01 |
| KO-OKC-03-041 | 2003 | Spawner | Kokanee | 1.00 | 0.00 | 0.00 |  | 0.98 | 0.02 |  | 0.99 | 0.00 | 0.01 |
| KO-OKC-03-042 | 2003 | Spawner | Kokanee | 1.00 | 0.00 | 0.00 |  | 0.99 | 0.02 |  | 0.97 | 0.00 | 0.03 |
| KO-OKC-03-043 | 2003 | Spawner | Kokanee | 1.00 | 0.00 | 0.00 |  | 0.99 | 0.01 |  | 1.00 | 0.00 | 0.00 |
| KO-OKC-03-044 | 2003 | Spawner | Kokanee | 1.00 | 0.00 | 0.00 |  | 0.97 | 0.03 |  | 0.88 | 0.00 | 0.12 |
| KO-OKC-03-045 | 2003 | Spawner | Kokanee | 1.00 | 0.00 | 0.00 |  | 0.99 | 0.01 |  | 1.00 | 0.00 | 0.00 |
| KO-OKC-03-046 | 2003 | Spawner | Kokanee | 1.00 | 0.00 | 0.00 |  | 0.98 | 0.02 |  | 0.99 | 0.00 | 0.01 |
| KO-OKC-03-047 | 2003 | Spawner | Kokanee | 1.00 | 0.00 | 0.00 |  | 0.99 | 0.01 |  | 1.00 | 0.00 | 0.00 |
| KO-OKC-03-048 | 2003 | Spawner | Kokanee | 1.00 | 0.00 | 0.00 |  | 0.99 | 0.01 |  | 1.00 | 0.00 | 0.00 |
| KO-OKC-03-049 | 2003 | Spawner | Kokanee | 1.00 | 0.00 | 0.00 |  | 0.99 | 0.01 |  | 1.00 | 0.00 | 0.00 |
| KO-OKC-03-050 | 2003 | Spawner | Kokanee | 1.00 | 0.00 | 0.00 |  | 0.99 | 0.01 |  | 1.00 | 0.00 | 0.00 |
| KO-OKC-03-051 | 2003 | Spawner | Kokanee | 0.96 | 0.00 | 0.03 |  | 0.82 | 0.19 |  | 0.31 | 0.00 | 0.69 |
| KO-OKC-03-052 | 2003 | Spawner | Kokanee | 1.00 | 0.00 | 0.00 |  | 0.99 | 0.01 |  | 1.00 | 0.00 | 0.00 |
| KO-OKC-03-053 | 2003 | Spawner | Kokanee | 1.00 | 0.00 | 0.00 |  | 0.99 | 0.01 |  | 1.00 | 0.00 | 0.00 |
| KO-OKC-03-054 | 2003 | Spawner | Kokanee | 1.00 | 0.00 | 0.00 |  | 0.99 | 0.01 |  | 1.00 | 0.00 | 0.00 |
| KO-OKC-03-055 | 2003 | Spawner | Kokanee | 1.00 | 0.00 | 0.00 |  | 0.99 | 0.01 |  | 1.00 | 0.00 | 0.00 |
| KO-OKC-03-056 | 2003 | Spawner | Kokanee | 0.98 | 0.00 | 0.02 |  | 0.72 | 0.28 |  | 0.87 | 0.00 | 0.13 |
| KO-OKC-03-057 | 2003 | Spawner | Kokanee | 1.00 | 0.00 | 0.00 |  | 1.00 | 0.01 |  | 1.00 | 0.00 | 0.00 |
| KO-OKC-03-058 | 2003 | Spawner | Kokanee | 1.00 | 0.00 | 0.00 |  | 0.99 | 0.01 |  | 1.00 | 0.00 | 0.00 |
| KO-OKC-03-059 | 2003 | Spawner | Kokanee | 0.99 | 0.00 | 0.01 |  | 0.93 | 0.07 |  | 0.21 | 0.00 | 0.79 |
| KO-OKC-03-060 | 2003 | Spawner | Kokanee | 1.00 | 0.00 | 0.00 |  | 0.99 | 0.02 |  | 0.99 | 0.00 | 0.01 |
| KO-OKC-03-061 | 2003 | Spawner | Kokanee | 0.98 | 0.00 | 0.02 |  | 0.92 | 0.08 |  | 0.86 | 0.00 | 0.14 |
| KO-OKC-03-062 | 2003 | Spawner | Kokanee | 1.00 | 0.00 | 0.00 |  | 0.99 | 0.01 |  | 1.00 | 0.00 | 0.00 |
| KO-OKC-03-063 | 2003 | Spawner | Kokanee | 1.00 | 0.00 | 0.00 |  | 0.99 | 0.01 |  | 1.00 | 0.00 | 0.00 |
| KO-OKC-03-064 | 2003 | Spawner | Kokanee | 1.00 | 0.00 | 0.00 |  | 0.99 | 0.01 |  | 1.00 | 0.00 | 0.00 |
| KO-OKC-03-065 | 2003 | Spawner | Kokanee | 0.99 | 0.00 | 0.01 |  | 0.92 | 0.08 |  | 0.63 | 0.00 | 0.37 |
| KO-OKC-03-066 | 2003 | Spawner | Kokanee | 1.00 | 0.00 | 0.00 |  | 0.96 | 0.05 |  | 0.88 | 0.00 | 0.12 |
| KO-OKC-03-067 | 2003 | Spawner | Kokanee | 1.00 | 0.00 | 0.00 |  | 0.97 | 0.03 |  | 0.94 | 0.00 | 0.06 |
| KO-OKC-03-068 | 2003 | Spawner | Kokanee | 1.00 | 0.00 | 0.00 |  | 0.99 | 0.01 |  | 0.99 | 0.00 | 0.01 |
| KO-OKC-03-069 | 2003 | Spawner | Kokanee | 1.00 | 0.00 | 0.00 |  | 0.99 | 0.01 |  | 1.00 | 0.00 | 0.00 |
| KO-OKC-03-070 | 2003 | Spawner | Kokanee | 1.00 | 0.00 | 0.00 |  | 0.99 | 0.01 |  | 1.00 | 0.00 | 0.00 |
| KO-OKC-03-071 | 2003 | Spawner | Kokanee | 1.00 | 0.00 | 0.00 |  | 0.99 | 0.01 |  | 1.00 | 0.00 | 0.00 |
| KO-OKC-03-072 | 2003 | Spawner | Kokanee | 1.00 | 0.00 | 0.00 |  | 0.99 | 0.02 |  | 0.98 | 0.00 | 0.02 |
| KO-OKC-03-073 | 2003 | Spawner | Kokanee | 1.00 | 0.00 | 0.00 |  | 0.98 | 0.02 |  | 0.99 | 0.00 | 0.01 |
| KO-OKC-03-074 | 2003 | Spawner | Kokanee | 1.00 | 0.00 | 0.00 |  | 0.99 | 0.01 |  | 1.00 | 0.00 | 0.00 |
| KO-OKC-03-075 | 2003 | Spawner | Kokanee | 1.00 | 0.00 | 0.00 |  | 0.99 | 0.01 |  | 1.00 | 0.00 | 0.00 |
| KO-OKC-03-076 | 2003 | Spawner | Kokanee | 1.00 | 0.00 | 0.00 |  | 0.97 | 0.03 |  | 0.86 | 0.00 | 0.14 |
| KO-OKC-03-077 | 2003 | Spawner | Kokanee | 1.00 | 0.00 | 0.00 |  | 0.99 | 0.01 |  | 1.00 | 0.00 | 0.00 |
| KO-OKC-03-078 | 2003 | Spawner | Kokanee | 0.98 | 0.00 | 0.02 |  | 0.88 | 0.12 |  | 0.79 | 0.00 | 0.21 |
| KO-OKC-03-079 | 2003 | Spawner | Kokanee | 0.98 | 0.00 | 0.02 |  | 0.95 | 0.05 |  | 0.90 | 0.00 | 0.10 |
| KO-OKC-03-080 | 2003 | Spawner | Kokanee | 1.00 | 0.00 | 0.00 |  | 0.99 | 0.02 |  | 0.99 | 0.00 | 0.01 |
| KO-OKC-03-081 | 2003 | Spawner | Kokanee | 1.00 | 0.00 | 0.00 |  | 0.98 | 0.02 |  | 0.99 | 0.00 | 0.01 |
| KO-OKC-03-082 | 2003 | Spawner | Kokanee | 1.00 | 0.00 | 0.00 |  | 0.99 | 0.01 |  | 1.00 | 0.00 | 0.00 |
| KO-OKC-03-083 | 2003 | Spawner | Kokanee | 1.00 | 0.00 | 0.00 |  | 0.97 | 0.03 |  | 0.96 | 0.00 | 0.04 |
| KO-OKC-03-084 | 2003 | Spawner | Kokanee | 1.00 | 0.00 | 0.00 |  | 0.99 | 0.01 |  | 1.00 | 0.00 | 0.00 |
| KO-OKC-03-085 | 2003 | Spawner | Kokanee | 1.00 | 0.00 | 0.00 |  | 0.99 | 0.01 |  | 1.00 | 0.00 | 0.00 |
| KO-OKC-03-086 | 2003 | Spawner | Kokanee | 1.00 | 0.00 | 0.00 |  | 0.99 | 0.02 |  | 1.00 | 0.00 | 0.00 |
| KO-OKC-03-087 | 2003 | Spawner | Kokanee | 0.96 | 0.00 | 0.04 |  | 0.96 | 0.04 |  | 0.79 | 0.00 | 0.21 |
| KO-OKC-03-088 | 2003 | Spawner | Kokanee | 0.99 | 0.00 | 0.01 |  | 0.95 | 0.05 |  | 0.78 | 0.00 | 0.22 |
| KO-OKC-03-089 | 2003 | Spawner | Kokanee | 1.00 | 0.00 | 0.00 |  | 0.99 | 0.01 |  | 1.00 | 0.00 | 0.00 |
| KO-OKC-03-090 | 2003 | Spawner | Kokanee | 1.00 | 0.00 | 0.00 |  | 0.99 | 0.01 |  | 1.00 | 0.00 | 0.00 |
| KO-OKC-03-091 | 2003 | Spawner | Kokanee | 1.00 | 0.00 | 0.00 |  | 0.97 | 0.03 |  | 0.98 | 0.00 | 0.02 |
| KO-OKC-03-092 | 2003 | Spawner | Kokanee | 1.00 | 0.00 | 0.00 |  | 0.99 | 0.01 |  | 1.00 | 0.00 | 0.00 |
| KO-OKC-03-093 | 2003 | Spawner | Kokanee | 1.00 | 0.00 | 0.00 |  | 0.95 | 0.05 |  | 0.84 | 0.00 | 0.16 |
| KO-OKC-03-094 | 2003 | Spawner | Kokanee | 1.00 | 0.00 | 0.00 |  | 0.99 | 0.01 |  | 1.00 | 0.00 | 0.00 |
| KO-OKC-03-095 | 2003 | Spawner | Kokanee | 0.33 | 0.01 | 0.67 |  | 0.58 | 0.42 |  | 0.01 | 0.00 | 0.98 |
| KO-OKC-03-096 | 2003 | Spawner | Kokanee | 1.00 | 0.00 | 0.00 |  | 0.99 | 0.01 |  | 1.00 | 0.00 | 0.00 |
| KO-SKA-03-006 | 2003 | Spawner | Kokanee | 1.00 | 0.00 | 0.00 |  | 0.99 | 0.01 |  | 1.00 | 0.00 | 0.00 |
| KO-SKA-03-007 | 2003 | Spawner | Kokanee | 1.00 | 0.00 | 0.00 |  | 0.99 | 0.01 |  | 1.00 | 0.00 | 0.00 |
| KO-SKA-03-008 | 2003 | Spawner | Kokanee | 1.00 | 0.00 | 0.00 |  | 0.98 | 0.02 |  | 1.00 | 0.00 | 0.00 |
| KO-SKA-03-009 | 2003 | Spawner | Kokanee | 1.00 | 0.00 | 0.00 |  | 0.98 | 0.02 |  | 0.97 | 0.00 | 0.03 |
| KO-SKA-03-010 | 2003 | Spawner | Kokanee | 0.99 | 0.00 | 0.01 |  | 0.95 | 0.05 |  | 0.79 | 0.00 | 0.21 |
| KO-SKA-03-011 | 2003 | Spawner | Kokanee | 1.00 | 0.00 | 0.00 |  | 0.99 | 0.01 |  | 1.00 | 0.00 | 0.00 |
| KO-SKA-03-012 | 2003 | Spawner | Kokanee | 0.97 | 0.00 | 0.03 |  | 0.92 | 0.08 |  | 0.53 | 0.00 | 0.47 |
| KO-SKA-03-013 | 2003 | Spawner | Kokanee | 1.00 | 0.00 | 0.00 |  | 0.98 | 0.02 |  | 0.98 | 0.00 | 0.02 |
| KO-SKA-03-014 | 2003 | Spawner | Kokanee | 1.00 | 0.00 | 0.00 |  | 0.95 | 0.05 |  | 0.80 | 0.00 | 0.20 |
| KO-SKA-03-015 | 2003 | Spawner | Kokanee | 1.00 | 0.00 | 0.00 |  | 0.99 | 0.01 |  | 1.00 | 0.00 | 0.00 |
| KO-SKA-03-016 | 2003 | Spawner | Kokanee | 1.00 | 0.00 | 0.00 |  | 0.99 | 0.01 |  | 1.00 | 0.00 | 0.00 |
| KO-SKA-03-017 | 2003 | Spawner | Kokanee | 1.00 | 0.00 | 0.00 |  | 0.97 | 0.03 |  | 0.95 | 0.00 | 0.05 |
| KO-SKA-03-018 | 2003 | Spawner | Kokanee | 1.00 | 0.00 | 0.00 |  | 0.99 | 0.01 |  | 1.00 | 0.00 | 0.00 |
| KO-SKA-03-019 | 2003 | Spawner | Kokanee | 1.00 | 0.00 | 0.00 |  | 0.99 | 0.01 |  | 1.00 | 0.00 | 0.00 |
| KO-SKA-03-020 | 2003 | Spawner | Kokanee | 1.00 | 0.00 | 0.00 |  | 0.99 | 0.01 |  | 1.00 | 0.00 | 0.00 |
| KO-SKA-03-021 | 2003 | Spawner | Kokanee | 1.00 | 0.00 | 0.00 |  | 0.99 | 0.01 |  | 1.00 | 0.00 | 0.00 |
| KO-SKA-03-022 | 2003 | Spawner | Kokanee | 1.00 | 0.00 | 0.00 |  | 0.98 | 0.02 |  | 1.00 | 0.00 | 0.00 |
| KO-SKA-03-023 | 2003 | Spawner | Kokanee | 1.00 | 0.00 | 0.00 |  | 0.98 | 0.02 |  | 0.99 | 0.00 | 0.01 |
| KO-SKA-03-024 | 2003 | Spawner | Kokanee | 1.00 | 0.00 | 0.00 |  | 0.97 | 0.03 |  | 0.93 | 0.00 | 0.07 |
| KO-SKA-03-025 | 2003 | Spawner | Kokanee | 1.00 | 0.00 | 0.00 |  | 0.99 | 0.01 |  | 1.00 | 0.00 | 0.00 |
| KO-SKA-03-026 | 2003 | Spawner | Kokanee | 1.00 | 0.00 | 0.00 |  | 0.99 | 0.01 |  | 1.00 | 0.00 | 0.00 |
| KO-SKA-03-027 | 2003 | Spawner | Kokanee | 1.00 | 0.00 | 0.00 |  | 0.97 | 0.03 |  | 0.93 | 0.00 | 0.07 |
| KO-SKA-03-028 | 2003 | Spawner | Kokanee | 0.96 | 0.00 | 0.04 |  | 0.93 | 0.07 |  | 0.36 | 0.00 | 0.64 |
| KO-SKA-03-029 | 2003 | Spawner | Kokanee | 1.00 | 0.00 | 0.00 |  | 0.99 | 0.01 |  | 1.00 | 0.00 | 0.00 |
| KO-SKA-03-030 | 2003 | Spawner | Kokanee | 1.00 | 0.00 | 0.00 |  | 0.99 | 0.01 |  | 1.00 | 0.00 | 0.00 |
| KO-SKA-03-031 | 2003 | Spawner | Kokanee | 1.00 | 0.00 | 0.00 |  | 0.97 | 0.03 |  | 0.93 | 0.00 | 0.07 |
| KO-SKA-03-032 | 2003 | Spawner | Kokanee | 1.00 | 0.00 | 0.00 |  | 0.98 | 0.02 |  | 0.99 | 0.00 | 0.01 |
| KO-SKA-03-033 | 2003 | Spawner | Kokanee | 1.00 | 0.00 | 0.00 |  | 0.98 | 0.02 |  | 0.99 | 0.00 | 0.01 |
| KO-SKA-03-034 | 2003 | Spawner | Kokanee | 0.75 | 0.00 | 0.25 |  | 0.77 | 0.23 |  | 0.08 | 0.00 | 0.92 |
| KO-SKA-03-035 | 2003 | Spawner | Kokanee | 1.00 | 0.00 | 0.00 |  | 0.97 | 0.03 |  | 0.93 | 0.00 | 0.07 |
| KO-SKA-03-036 | 2003 | Spawner | Kokanee | 0.99 | 0.00 | 0.01 |  | 0.95 | 0.05 |  | 0.84 | 0.00 | 0.16 |
| KO-SKA-03-037 | 2003 | Spawner | Kokanee | 1.00 | 0.00 | 0.00 |  | 0.98 | 0.03 |  | 0.96 | 0.00 | 0.04 |
| KO-SKA-03-038 | 2003 | Spawner | Kokanee | 1.00 | 0.00 | 0.00 |  | 0.98 | 0.02 |  | 0.95 | 0.00 | 0.05 |
| KO-SKA-03-039 | 2003 | Spawner | Kokanee | 1.00 | 0.00 | 0.00 |  | 0.99 | 0.01 |  | 1.00 | 0.00 | 0.00 |
| KO-SKA-03-040 | 2003 | Spawner | Kokanee | 1.00 | 0.00 | 0.00 |  | 0.98 | 0.02 |  | 0.99 | 0.00 | 0.01 |
| SK-OKR-12-002 | 2012 | Spawner | Sockeye | 0.00 | 1.00 | 0.00 |  | 0.01 | 0.99 |  | 0.00 | 1.00 | 0.00 |
| SK-OKR-12-006 | 2012 | Spawner | Sockeye | 0.00 | 1.00 | 0.00 |  | 0.01 | 0.99 |  | 0.00 | 1.00 | 0.00 |
| SK-OKR-12-010 | 2012 | Spawner | Sockeye | 0.01 | 0.14 | 0.85 |  | 0.24 | 0.76 |  | 0.00 | 0.12 | 0.88 |
| SK-OKR-12-014 | 2012 | Spawner | Sockeye | 0.00 | 1.00 | 0.00 |  | 0.01 | 0.99 |  | 0.00 | 1.00 | 0.00 |
| SK-OKR-12-018 | 2012 | Spawner | Sockeye | 0.00 | 1.00 | 0.00 |  | 0.02 | 0.98 |  | 0.00 | 0.98 | 0.02 |
| SK-OKR-12-022 | 2012 | Spawner | Sockeye | 0.00 | 1.00 | 0.00 |  | 0.01 | 0.99 |  | 0.00 | 0.99 | 0.01 |
| SK-OKR-12-026 | 2012 | Spawner | Sockeye | 0.00 | 1.00 | 0.00 |  | 0.02 | 0.98 |  | 0.00 | 0.99 | 0.01 |
| SK-OKR-12-030 | 2012 | Spawner | Sockeye | 0.00 | 0.91 | 0.09 |  | 0.04 | 0.96 |  | 0.00 | 0.95 | 0.05 |
| SK-OKR-12-030a | 2012 | Spawner | Sockeye | 0.00 | 0.73 | 0.27 |  | 0.11 | 0.89 |  | 0.00 | 0.49 | 0.51 |
| SK-OKR-12-034 | 2012 | Spawner | Sockeye | 0.00 | 1.00 | 0.00 |  | 0.04 | 0.96 |  | 0.00 | 0.91 | 0.09 |
| SK-OKR-12-038 | 2012 | Spawner | Sockeye | 0.00 | 1.00 | 0.00 |  | 0.01 | 0.99 |  | 0.00 | 1.00 | 0.00 |
| SK-OKR-12-042 | 2012 | Spawner | Sockeye | 0.00 | 1.00 | 0.00 |  | 0.01 | 0.99 |  | 0.00 | 1.00 | 0.00 |
| SK-OKR-12-046 | 2012 | Spawner | Sockeye | 0.00 | 1.00 | 0.00 |  | 0.02 | 0.98 |  | 0.00 | 0.96 | 0.04 |
| SK-OKR-12-050 | 2012 | Spawner | Sockeye | 0.00 | 1.00 | 0.00 |  | 0.01 | 0.99 |  | 0.00 | 1.00 | 0.00 |
| SK-OKR-12-054 | 2012 | Spawner | Sockeye | 0.00 | 1.00 | 0.00 |  | 0.01 | 0.99 |  | 0.00 | 1.00 | 0.00 |
| SK-OKR-12-058 | 2012 | Spawner | Sockeye | 0.00 | 0.99 | 0.01 |  | 0.02 | 0.98 |  | 0.00 | 0.95 | 0.05 |
| SK-OKR-12-062 | 2012 | Spawner | Sockeye | 0.00 | 1.00 | 0.00 |  | 0.01 | 0.99 |  | 0.00 | 1.00 | 0.00 |
| SK-OKR-12-066 | 2012 | Spawner | Sockeye | 0.00 | 1.00 | 0.00 |  | 0.01 | 0.99 |  | 0.00 | 1.00 | 0.00 |
| SK-OKR-12-070 | 2012 | Spawner | Sockeye | 0.00 | 1.00 | 0.00 |  | 0.03 | 0.97 |  | 0.00 | 0.97 | 0.03 |
| SK-OKR-12-074 | 2012 | Spawner | Sockeye | 0.00 | 1.00 | 0.00 |  | 0.01 | 1.00 |  | 0.00 | 1.00 | 0.00 |
| SK-OKR-12-078 | 2012 | Spawner | Sockeye | 0.00 | 1.00 | 0.00 |  | 0.02 | 0.99 |  | 0.00 | 1.00 | 0.00 |
| SK-OKR-12-082 | 2012 | Spawner | Sockeye | 0.00 | 1.00 | 0.00 |  | 0.01 | 1.00 |  | 0.00 | 1.00 | 0.00 |
| SK-OKR-12-090 | 2012 | Spawner | Sockeye | 0.00 | 1.00 | 0.00 |  | 0.01 | 0.99 |  | 0.00 | 1.00 | 0.00 |
| SK-OKR-12-094 | 2012 | Spawner | Sockeye | 0.00 | 1.00 | 0.00 |  | 0.01 | 0.99 |  | 0.00 | 1.00 | 0.00 |
| SK-OKR-12-098 | 2012 | Spawner | Sockeye | 0.00 | 1.00 | 0.00 |  | 0.02 | 0.98 |  | 0.00 | 0.98 | 0.02 |
| SK-OKR-12-105 | 2012 | Spawner | Sockeye | 0.00 | 1.00 | 0.00 |  | 0.01 | 0.99 |  | 0.00 | 1.00 | 0.00 |
| SK-OKR-12-109 | 2012 | Spawner | Sockeye | 0.00 | 1.00 | 0.00 |  | 0.01 | 0.99 |  | 0.00 | 1.00 | 0.00 |
| SK-OKR-12-113 | 2012 | Spawner | Sockeye | 0.00 | 1.00 | 0.00 |  | 0.01 | 0.99 |  | 0.00 | 1.00 | 0.00 |
| SK-OKR-12-117 | 2012 | Spawner | Sockeye | 0.00 | 1.00 | 0.00 |  | 0.01 | 0.99 |  | 0.00 | 1.00 | 0.00 |
| SK-OKR-12-122 | 2012 | Spawner | Sockeye | 0.00 | 0.19 | 0.81 |  | 0.16 | 0.85 |  | 0.00 | 0.16 | 0.84 |
| SK-OKR-12-126 | 2012 | Spawner | Sockeye | 0.00 | 0.99 | 0.01 |  | 0.03 | 0.97 |  | 0.00 | 0.95 | 0.05 |
| SK-OKR-12-132 | 2012 | Spawner | Sockeye | 0.00 | 1.00 | 0.00 |  | 0.02 | 0.98 |  | 0.00 | 0.99 | 0.01 |
| SK-OKR-12-136 | 2012 | Spawner | Sockeye | 0.00 | 1.00 | 0.00 |  | 0.01 | 0.99 |  | 0.00 | 1.00 | 0.00 |
| SK-OKR-12-139 | 2012 | Spawner | Sockeye | 0.00 | 0.99 | 0.01 |  | 0.03 | 0.97 |  | 0.00 | 0.96 | 0.04 |
| SK-OKR-12-143 | 2012 | Spawner | Sockeye | 0.00 | 0.98 | 0.02 |  | 0.04 | 0.96 |  | 0.00 | 0.77 | 0.23 |
| SK-OKR-12-147 | 2012 | Spawner | Sockeye | 0.00 | 0.82 | 0.18 |  | 0.12 | 0.88 |  | 0.00 | 0.27 | 0.73 |
| SK-OKR-12-151 | 2012 | Spawner | Sockeye | 0.00 | 1.00 | 0.00 |  | 0.02 | 0.98 |  | 0.00 | 0.99 | 0.01 |
| SK-OKR-12-155 | 2012 | Spawner | Sockeye | 0.00 | 0.97 | 0.03 |  | 0.06 | 0.94 |  | 0.00 | 0.49 | 0.51 |
| SK-OKR-12-160 | 2012 | Spawner | Sockeye | 0.00 | 0.99 | 0.01 |  | 0.02 | 0.98 |  | 0.00 | 0.98 | 0.02 |
| SK-OKR-12-164 | 2012 | Spawner | Sockeye | 0.00 | 1.00 | 0.00 |  | 0.01 | 0.99 |  | 0.00 | 0.99 | 0.01 |
| SK-OKR-12-168 | 2012 | Spawner | Sockeye | 0.00 | 1.00 | 0.00 |  | 0.01 | 0.99 |  | 0.00 | 1.00 | 0.00 |
| SK-OKR-12-172 | 2012 | Spawner | Sockeye | 0.00 | 1.00 | 0.00 |  | 0.01 | 0.99 |  | 0.00 | 1.00 | 0.00 |
| SK-OKR-12-176 | 2012 | Spawner | Sockeye | 0.00 | 1.00 | 0.00 |  | 0.02 | 0.98 |  | 0.00 | 0.97 | 0.03 |
| SK-OKR-12-180 | 2012 | Spawner | Sockeye | 0.00 | 1.00 | 0.00 |  | 0.01 | 0.99 |  | 0.00 | 1.00 | 0.00 |
| SK-OKR-12-182 | 2012 | Spawner | Sockeye | 0.00 | 1.00 | 0.00 |  | 0.02 | 0.99 |  | 0.00 | 0.99 | 0.01 |
| SK-OKR-12-185 | 2012 | Spawner | Sockeye | 0.00 | 1.00 | 0.00 |  | 0.01 | 0.99 |  | 0.00 | 1.00 | 0.00 |
| SK-OKR-12-189 | 2012 | Spawner | Sockeye | 0.00 | 1.00 | 0.00 |  | 0.01 | 0.99 |  | 0.00 | 1.00 | 0.00 |
| SK-OKR-12-194 | 2012 | Spawner | Sockeye | 0.00 | 1.00 | 0.00 |  | 0.01 | 1.00 |  | 0.00 | 1.00 | 0.00 |
| SK-OKR-12-198 | 2012 | Spawner | Sockeye | 0.00 | 1.00 | 0.00 |  | 0.01 | 0.99 |  | 0.00 | 1.00 | 0.00 |
| SK-OKR-12-301a | 2012 | Spawner | Sockeye | 0.00 | 1.00 | 0.00 |  | 0.01 | 0.99 |  | 0.00 | 1.00 | 0.00 |
| SK-OKR-12-305a | 2012 | Spawner | Sockeye | 0.00 | 1.00 | 0.00 |  | 0.01 | 0.99 |  | 0.00 | 1.00 | 0.00 |
| SK-OKR-12-307a | 2012 | Spawner | Sockeye | 0.00 | 0.99 | 0.01 |  | 0.02 | 0.98 |  | 0.00 | 0.98 | 0.02 |
| SK-OKR-12-309a | 2012 | Spawner | Sockeye | 0.00 | 1.00 | 0.00 |  | 0.01 | 0.99 |  | 0.00 | 1.00 | 0.00 |
| SK-OKR-12-311a | 2012 | Spawner | Sockeye | 0.00 | 0.95 | 0.05 |  | 0.03 | 0.97 |  | 0.00 | 0.96 | 0.04 |
| SK-OKR-12-312a | 2012 | Spawner | Sockeye | 0.00 | 1.00 | 0.00 |  | 0.01 | 0.99 |  | 0.00 | 0.99 | 0.01 |
| SK-OKR-12-315a | 2012 | Spawner | Sockeye | 0.00 | 1.00 | 0.00 |  | 0.01 | 0.99 |  | 0.00 | 1.00 | 0.00 |
| SK-OKR-12-318a | 2012 | Spawner | Sockeye | 0.00 | 0.99 | 0.01 |  | 0.02 | 0.98 |  | 0.00 | 1.00 | 0.00 |
| SK-OKR-12-319a | 2012 | Spawner | Sockeye | 0.00 | 0.98 | 0.02 |  | 0.03 | 0.97 |  | 0.00 | 0.96 | 0.04 |
| SK-OKR-12-322a | 2012 | Spawner | Sockeye | 0.00 | 1.00 | 0.00 |  | 0.02 | 0.98 |  | 0.00 | 0.99 | 0.01 |
| SK-OKR-12-323a | 2012 | Spawner | Sockeye | 0.00 | 1.00 | 0.00 |  | 0.03 | 0.98 |  | 0.00 | 0.96 | 0.04 |
| SK-OKR-12-325a | 2012 | Spawner | Sockeye | 0.00 | 0.91 | 0.09 |  | 0.10 | 0.90 |  | 0.00 | 0.41 | 0.59 |
| SK-OKR-12-327a | 2012 | Spawner | Sockeye | 0.00 | 1.00 | 0.00 |  | 0.02 | 0.98 |  | 0.00 | 0.98 | 0.02 |
| SK-OKR-12-329a | 2012 | Spawner | Sockeye | 0.00 | 1.00 | 0.00 |  | 0.02 | 0.98 |  | 0.00 | 0.98 | 0.02 |
| SK-OKR-12-331a | 2012 | Spawner | Sockeye | 0.00 | 0.58 | 0.42 |  | 0.08 | 0.92 |  | 0.00 | 0.48 | 0.52 |
| SK-OKR-12-333a | 2012 | Spawner | Sockeye | 0.00 | 0.74 | 0.26 |  | 0.06 | 0.94 |  | 0.00 | 0.77 | 0.23 |
| SK-OKR-12-336a | 2012 | Spawner | Sockeye | 0.00 | 1.00 | 0.00 |  | 0.02 | 0.98 |  | 0.00 | 0.98 | 0.02 |
| SK-OKR-12-338a | 2012 | Spawner | Sockeye | 0.00 | 0.99 | 0.01 |  | 0.03 | 0.97 |  | 0.00 | 0.92 | 0.08 |
| SK-OKR-12-339a | 2012 | Spawner | Sockeye | 0.00 | 0.99 | 0.01 |  | 0.04 | 0.96 |  | 0.00 | 0.85 | 0.15 |
| SK-OKR-12-341a | 2012 | Spawner | Sockeye | 0.00 | 0.99 | 0.01 |  | 0.03 | 0.97 |  | 0.00 | 0.97 | 0.03 |
| SK-OKR-12-343a | 2012 | Spawner | Sockeye | 0.00 | 1.00 | 0.00 |  | 0.02 | 0.98 |  | 0.00 | 0.98 | 0.02 |
| SK-OKR-12-345a | 2012 | Spawner | Sockeye | 0.00 | 0.99 | 0.01 |  | 0.04 | 0.97 |  | 0.00 | 0.88 | 0.12 |
| SK-OKR-12-347a | 2012 | Spawner | Sockeye | 0.00 | 1.00 | 0.00 |  | 0.02 | 0.98 |  | 0.00 | 0.99 | 0.01 |
| SK-OKR-12-350a | 2012 | Spawner | Sockeye | 0.00 | 1.00 | 0.00 |  | 0.01 | 0.99 |  | 0.00 | 1.00 | 0.00 |
| SK-OKR-12-351a | 2012 | Spawner | Sockeye | 0.00 | 1.00 | 0.00 |  | 0.01 | 0.99 |  | 0.00 | 1.00 | 0.00 |
| SK-OKR-12-352a | 2012 | Spawner | Sockeye | 0.00 | 1.00 | 0.00 |  | 0.01 | 0.99 |  | 0.00 | 0.99 | 0.01 |
| SK-OKR-12-356a | 2012 | Spawner | Sockeye | 0.00 | 1.00 | 0.00 |  | 0.01 | 0.99 |  | 0.00 | 1.00 | 0.00 |
| SK-OKR-12-357a | 2012 | Spawner | Sockeye | 0.00 | 1.00 | 0.00 |  | 0.01 | 0.99 |  | 0.00 | 0.99 | 0.01 |
| SK-OKR-12-360a | 2012 | Spawner | Sockeye | 0.00 | 0.99 | 0.01 |  | 0.04 | 0.96 |  | 0.00 | 0.96 | 0.04 |
| SK-OKR-12-361a | 2012 | Spawner | Sockeye | 0.00 | 1.00 | 0.00 |  | 0.02 | 0.98 |  | 0.00 | 0.99 | 0.01 |
| SK-OKR-12-364a | 2012 | Spawner | Sockeye | 0.00 | 1.00 | 0.00 |  | 0.01 | 0.99 |  | 0.00 | 1.00 | 0.00 |
| SK-OKR-12-365a | 2012 | Spawner | Sockeye | 0.00 | 1.00 | 0.00 |  | 0.01 | 0.99 |  | 0.00 | 1.00 | 0.00 |
| SK-OKR-12-368a | 2012 | Spawner | Sockeye | 0.00 | 1.00 | 0.00 |  | 0.01 | 0.99 |  | 0.00 | 1.00 | 0.00 |
| SK-OKR-12-369a | 2012 | Spawner | Sockeye | 0.00 | 1.00 | 0.00 |  | 0.01 | 0.99 |  | 0.00 | 1.00 | 0.00 |
| SK-OKR-12-371a | 2012 | Spawner | Sockeye | 0.00 | 1.00 | 0.00 |  | 0.01 | 0.99 |  | 0.00 | 1.00 | 0.00 |
| SK-OKR-12-373a | 2012 | Spawner | Sockeye | 0.00 | 1.00 | 0.00 |  | 0.02 | 0.98 |  | 0.00 | 0.99 | 0.01 |
| SK-OKR-12-375a | 2012 | Spawner | Sockeye | 0.00 | 1.00 | 0.00 |  | 0.02 | 0.98 |  | 0.00 | 0.99 | 0.01 |
| SK-OKR-12-376a | 2012 | Spawner | Sockeye | 0.00 | 1.00 | 0.00 |  | 0.01 | 0.99 |  | 0.00 | 1.00 | 0.00 |
| SK-OKR-12-378a | 2012 | Spawner | Sockeye | 0.00 | 0.84 | 0.16 |  | 0.13 | 0.87 |  | 0.00 | 0.32 | 0.68 |
| SK-OKR-12-381a | 2012 | Spawner | Sockeye | 0.00 | 1.00 | 0.00 |  | 0.03 | 0.97 |  | 0.00 | 0.98 | 0.02 |
| SK-OKR-12-382a | 2012 | Spawner | Sockeye | 0.00 | 1.00 | 0.00 |  | 0.02 | 0.98 |  | 0.00 | 0.94 | 0.06 |
| SK-OKR-12-385a | 2012 | Spawner | Sockeye | 0.00 | 1.00 | 0.00 |  | 0.01 | 0.99 |  | 0.00 | 0.99 | 0.01 |
| SK-OKR-12-387a | 2012 | Spawner | Sockeye | 0.00 | 0.95 | 0.05 |  | 0.04 | 0.96 |  | 0.00 | 0.80 | 0.20 |
| SK-OKR-12-389a | 2012 | Spawner | Sockeye | 0.00 | 1.00 | 0.00 |  | 0.01 | 0.99 |  | 0.00 | 0.99 | 0.01 |
| SK-OKR-12-392a | 2012 | Spawner | Sockeye | 0.00 | 1.00 | 0.00 |  | 0.09 | 0.91 |  | 0.00 | 0.98 | 0.02 |
| SK-OKR-12-393a | 2012 | Spawner | Sockeye | 0.01 | 0.78 | 0.22 |  | 0.14 | 0.86 |  | 0.00 | 0.50 | 0.50 |
| SK-OKR-12-395a | 2012 | Spawner | Sockeye | 0.00 | 1.00 | 0.00 |  | 0.02 | 0.98 |  | 0.00 | 1.00 | 0.00 |
| SK-OKR-12-397a | 2012 | Spawner | Sockeye | 0.00 | 1.00 | 0.00 |  | 0.01 | 0.99 |  | 0.00 | 0.99 | 0.01 |
| SK-OKR-12-400 | 2012 | Spawner | Sockeye | 0.00 | 1.00 | 0.00 |  | 0.02 | 0.98 |  | 0.00 | 0.99 | 0.01 |
| SK-OKR-12-404 | 2012 | Spawner | Sockeye | 0.00 | 1.00 | 0.00 |  | 0.01 | 0.99 |  | 0.00 | 1.00 | 0.00 |
| SK-OKR-12-409 | 2012 | Spawner | Sockeye | 0.00 | 1.00 | 0.00 |  | 0.01 | 0.99 |  | 0.00 | 1.00 | 0.00 |
| SK-OKR-12-413 | 2012 | Spawner | Sockeye | 0.00 | 0.95 | 0.05 |  | 0.05 | 0.95 |  | 0.00 | 0.91 | 0.09 |
| SK-OKR-12-416 | 2012 | Spawner | Sockeye | 0.00 | 1.00 | 0.00 |  | 0.01 | 0.99 |  | 0.00 | 1.00 | 0.00 |
| SK-OKR-12-420 | 2012 | Spawner | Sockeye | 0.00 | 1.00 | 0.00 |  | 0.01 | 0.99 |  | 0.00 | 1.00 | 0.00 |
| SK-OKR-12-436 | 2012 | Spawner | Sockeye | 0.00 | 1.00 | 0.00 |  | 0.01 | 0.99 |  | 0.00 | 1.00 | 0.00 |
| SK-OKR-12-437 | 2012 | Spawner | Sockeye | 0.00 | 1.00 | 0.00 |  | 0.01 | 0.99 |  | 0.00 | 1.00 | 0.00 |
| SK-OKR-12-438 | 2012 | Spawner | Sockeye | 0.00 | 0.21 | 0.79 |  | 0.19 | 0.81 |  | 0.00 | 0.12 | 0.88 |
| SK-OKR-12-439 | 2012 | Spawner | Sockeye | 0.00 | 1.00 | 0.00 |  | 0.01 | 0.99 |  | 0.00 | 1.00 | 0.00 |
| SK-OKR-12-440 | 2012 | Spawner | Sockeye | 0.00 | 1.00 | 0.00 |  | 0.01 | 0.99 |  | 0.00 | 1.00 | 0.00 |
| SK-OKR-12-444 | 2012 | Spawner | Sockeye | 0.00 | 1.00 | 0.00 |  | 0.02 | 0.98 |  | 0.00 | 0.98 | 0.02 |
| SK-OKR-12-449 | 2012 | Spawner | Sockeye | 0.00 | 0.89 | 0.11 |  | 0.06 | 0.95 |  | 0.00 | 0.81 | 0.19 |
| SK-OKR-12-453 | 2012 | Spawner | Sockeye | 0.00 | 1.00 | 0.00 |  | 0.01 | 0.99 |  | 0.00 | 1.00 | 0.00 |
| SK-OKR-12-456 | 2012 | Spawner | Sockeye | 0.00 | 1.00 | 0.00 |  | 0.01 | 0.99 |  | 0.00 | 1.00 | 0.00 |
| SK-OKR-12-460 | 2012 | Spawner | Sockeye | 0.00 | 1.00 | 0.00 |  | 0.01 | 0.99 |  | 0.00 | 1.00 | 0.00 |
| SK-OKR-12-463 | 2012 | Spawner | Sockeye | 0.00 | 1.00 | 0.00 |  | 0.01 | 0.99 |  | 0.00 | 1.00 | 0.00 |
| SK-OKR-12-469 | 2012 | Spawner | Sockeye | 0.00 | 0.99 | 0.01 |  | 0.03 | 0.97 |  | 0.00 | 0.96 | 0.04 |
| SK-OKR-12-474 | 2012 | Spawner | Sockeye | 0.00 | 1.00 | 0.00 |  | 0.01 | 0.99 |  | 0.00 | 1.00 | 0.00 |
| SK-OKR-12-476 | 2012 | Spawner | Sockeye | 0.00 | 1.00 | 0.00 |  | 0.02 | 0.98 |  | 0.00 | 0.96 | 0.04 |
| SK-OKR-12-480 | 2012 | Spawner | Sockeye | 0.00 | 1.00 | 0.00 |  | 0.05 | 0.95 |  | 0.00 | 0.98 | 0.02 |
| SK-OKR-12-484 | 2012 | Spawner | Sockeye | 0.00 | 0.95 | 0.05 |  | 0.03 | 0.97 |  | 0.00 | 0.96 | 0.04 |
| SK-OKR-12-489 | 2012 | Spawner | Sockeye | 0.00 | 1.00 | 0.00 |  | 0.01 | 0.99 |  | 0.00 | 1.00 | 0.00 |
| SK-OKR-12-494 | 2012 | Spawner | Sockeye | 0.00 | 1.00 | 0.00 |  | 0.02 | 0.98 |  | 0.00 | 0.95 | 0.05 |
| SK-OKR-12-497 | 2012 | Spawner | Sockeye | 0.00 | 0.88 | 0.12 |  | 0.08 | 0.92 |  | 0.00 | 0.79 | 0.21 |
| SK-OKR-12-500 | 2012 | Spawner | Sockeye | 0.00 | 1.00 | 0.00 |  | 0.01 | 0.99 |  | 0.00 | 0.99 | 0.01 |
| SK-OKR-12-504 | 2012 | Spawner | Sockeye | 0.00 | 1.00 | 0.00 |  | 0.08 | 0.92 |  | 0.00 | 0.99 | 0.01 |
| SK-OKR-12-507 | 2012 | Spawner | Sockeye | 0.00 | 1.00 | 0.00 |  | 0.02 | 0.98 |  | 0.00 | 0.99 | 0.01 |
| SK-OKR-12-514 | 2012 | Spawner | Sockeye | 0.00 | 1.00 | 0.00 |  | 0.02 | 0.98 |  | 0.00 | 0.99 | 0.01 |
| SK-OKR-12-518 | 2012 | Spawner | Sockeye | 0.00 | 1.00 | 0.00 |  | 0.01 | 0.99 |  | 0.00 | 1.00 | 0.00 |
| SK-OKR-12-520 | 2012 | Spawner | Sockeye | 0.00 | 1.00 | 0.00 |  | 0.01 | 0.99 |  | 0.00 | 1.00 | 0.00 |
| SK-OKR-12-522 | 2012 | Spawner | Sockeye | 0.00 | 1.00 | 0.00 |  | 0.01 | 0.99 |  | 0.00 | 1.00 | 0.00 |
| SK-OKR-12-528 | 2012 | Spawner | Sockeye | 0.00 | 1.00 | 0.00 |  | 0.02 | 0.98 |  | 0.00 | 0.98 | 0.02 |
| SK-OKR-12-531 | 2012 | Spawner | Sockeye | 0.00 | 1.00 | 0.00 |  | 0.01 | 0.99 |  | 0.00 | 1.00 | 0.00 |
| SK-OKR-12-534 | 2012 | Spawner | Sockeye | 0.00 | 1.00 | 0.00 |  | 0.01 | 0.99 |  | 0.00 | 1.00 | 0.00 |
| SK-OKR-12-539 | 2012 | Spawner | Sockeye | 0.00 | 1.00 | 0.00 |  | 0.01 | 0.99 |  | 0.00 | 0.99 | 0.01 |
| SK-OKR-12-543 | 2012 | Spawner | Sockeye | 0.02 | 0.82 | 0.16 |  | 0.16 | 0.85 |  | 0.00 | 0.21 | 0.79 |
| SK-OKR-12-546 | 2012 | Spawner | Sockeye | 0.00 | 1.00 | 0.00 |  | 0.01 | 0.99 |  | 0.00 | 1.00 | 0.00 |
| SK-OKR-12-550 | 2012 | Spawner | Sockeye | 0.00 | 0.99 | 0.01 |  | 0.02 | 0.98 |  | 0.00 | 0.98 | 0.02 |
| SK-OKR-12-555 | 2012 | Spawner | Sockeye | 0.00 | 0.71 | 0.29 |  | 0.09 | 0.91 |  | 0.00 | 0.69 | 0.31 |
| SK-OKR-12-560 | 2012 | Spawner | Sockeye | 0.00 | 1.00 | 0.00 |  | 0.03 | 0.97 |  | 0.00 | 0.95 | 0.05 |
| SK-OKR-12-562 | 2012 | Spawner | Sockeye | 0.00 | 1.00 | 0.00 |  | 0.03 | 0.97 |  | 0.00 | 0.93 | 0.07 |
| SK-OKR-12-566 | 2012 | Spawner | Sockeye | 0.00 | 0.15 | 0.85 |  | 0.16 | 0.84 |  | 0.00 | 0.11 | 0.89 |
| SK-OKR-12-572 | 2012 | Spawner | Sockeye | 0.00 | 1.00 | 0.00 |  | 0.01 | 0.99 |  | 0.00 | 0.99 | 0.01 |
| SK-OKR-12-573 | 2012 | Spawner | Sockeye | 0.00 | 1.00 | 0.00 |  | 0.01 | 0.99 |  | 0.00 | 1.00 | 0.00 |
| SK-OKR-12-576 | 2012 | Spawner | Sockeye | 0.00 | 1.00 | 0.00 |  | 0.02 | 0.98 |  | 0.00 | 0.99 | 0.01 |
| SK-OKR-12-582 | 2012 | Spawner | Sockeye | 0.00 | 1.00 | 0.00 |  | 0.01 | 0.99 |  | 0.00 | 1.00 | 0.00 |
| SK-OKR-12-585 | 2012 | Spawner | Sockeye | 0.00 | 0.99 | 0.01 |  | 0.04 | 0.96 |  | 0.00 | 0.92 | 0.08 |
| SK-OKR-12-589 | 2012 | Spawner | Sockeye | 0.00 | 1.00 | 0.00 |  | 0.01 | 0.99 |  | 0.00 | 1.00 | 0.00 |
| SK-OKR-12-597 | 2012 | Spawner | Sockeye | 0.00 | 1.00 | 0.00 |  | 0.01 | 0.99 |  | 0.00 | 1.00 | 0.00 |
| SK-OKR-12-598 | 2012 | Spawner | Sockeye | 0.00 | 1.00 | 0.00 |  | 0.02 | 0.98 |  | 0.00 | 1.00 | 0.00 |
| SK08_07243 | 2008 | Trawl | Age 0 | 1.00 | 0.00 | 0.00 |  | 0.99 | 0.01 |  | 1.00 | 0.00 | 0.00 |
| SK08_07313 | 2008 | Trawl | Age 0 | 1.00 | 0.00 | 0.00 |  | 0.99 | 0.01 |  | 0.85 | 0.00 | 0.15 |
| SK08_07320 | 2008 | Trawl | Age 0 | 1.00 | 0.00 | 0.00 |  | 0.99 | 0.01 |  | 1.00 | 0.00 | 0.00 |
| SK08_07354 | 2008 | Trawl | Age 0 | 1.00 | 0.00 | 0.00 |  | 0.93 | 0.07 |  | 0.84 | 0.00 | 0.16 |
| SK08_07364 | 2008 | Trawl | Age 0 | 0.99 | 0.00 | 0.01 |  | 0.96 | 0.04 |  | 0.85 | 0.00 | 0.15 |
| SK08_07368 | 2008 | Trawl | Age 0 | 0.93 | 0.00 | 0.07 |  | 0.78 | 0.22 |  | 0.08 | 0.00 | 0.92 |
| SK08_07392 | 2008 | Trawl | Age 0 | 1.00 | 0.00 | 0.00 |  | 0.99 | 0.01 |  | 0.99 | 0.00 | 0.01 |
| SK08_07440 | 2008 | Trawl | Age 0 | 1.00 | 0.00 | 0.00 |  | 0.99 | 0.01 |  | 1.00 | 0.00 | 0.00 |
| SK08_07445 | 2008 | Trawl | Age 0 | 1.00 | 0.00 | 0.00 |  | 0.98 | 0.02 |  | 0.98 | 0.00 | 0.02 |
| SK08_07536 | 2008 | Trawl | Age 0 | 1.00 | 0.00 | 0.00 |  | 0.97 | 0.03 |  | 0.91 | 0.00 | 0.09 |
| SK08_07592 | 2008 | Trawl | Age 0 | 0.87 | 0.00 | 0.13 |  | 0.72 | 0.28 |  | 0.02 | 0.00 | 0.98 |
| SK08_07617 | 2008 | Trawl | Age 0 | 1.00 | 0.00 | 0.00 |  | 0.99 | 0.01 |  | 1.00 | 0.00 | 0.00 |
| SK08_07638 | 2008 | Trawl | Age 0 | 1.00 | 0.00 | 0.00 |  | 0.99 | 0.01 |  | 1.00 | 0.00 | 0.00 |
| SK08_07696 | 2008 | Trawl | Age 0 | 1.00 | 0.00 | 0.00 |  | 0.95 | 0.05 |  | 0.72 | 0.00 | 0.28 |
| SK08_07703 | 2008 | Trawl | Age 0 | 1.00 | 0.00 | 0.00 |  | 0.98 | 0.02 |  | 0.95 | 0.00 | 0.05 |
| SK08_07711 | 2008 | Trawl | Age 0 | 1.00 | 0.00 | 0.00 |  | 0.99 | 0.01 |  | 0.99 | 0.00 | 0.01 |
| SK08_7201 | 2008 | Trawl | Age 0 | 1.00 | 0.00 | 0.00 |  | 0.99 | 0.01 |  | 1.00 | 0.00 | 0.00 |
| SK08_7204 | 2008 | Trawl | Age 0 | 1.00 | 0.00 | 0.00 |  | 0.99 | 0.01 |  | 1.00 | 0.00 | 0.00 |
| SK08_7205 | 2008 | Trawl | Age 0 | 1.00 | 0.00 | 0.00 |  | 0.99 | 0.01 |  | 0.99 | 0.00 | 0.01 |
| SK08_7208 | 2008 | Trawl | Age 0 | 1.00 | 0.00 | 0.00 |  | 0.99 | 0.01 |  | 1.00 | 0.00 | 0.00 |
| SK08_7222 | 2008 | Trawl | Age 0 | 0.99 | 0.00 | 0.01 |  | 0.94 | 0.06 |  | 0.64 | 0.00 | 0.36 |
| SK08_7231 | 2008 | Trawl | Age 0 | 0.99 | 0.00 | 0.01 |  | 0.94 | 0.07 |  | 0.74 | 0.00 | 0.26 |
| SK08_7252 | 2008 | Trawl | Age 0 | 0.99 | 0.00 | 0.01 |  | 0.95 | 0.05 |  | 0.87 | 0.00 | 0.13 |
| SK08_7256 | 2008 | Trawl | Age 0 | 0.99 | 0.00 | 0.01 |  | 0.83 | 0.17 |  | 0.38 | 0.00 | 0.62 |
| SK08_7304 | 2008 | Trawl | Age 0 | 1.00 | 0.00 | 0.00 |  | 0.95 | 0.05 |  | 0.71 | 0.00 | 0.29 |
| SK08_7323 | 2008 | Trawl | Age 0 | 1.00 | 0.00 | 0.00 |  | 0.97 | 0.03 |  | 0.87 | 0.00 | 0.13 |
| SK08_7347 | 2008 | Trawl | Age 0 | 0.99 | 0.00 | 0.01 |  | 0.95 | 0.05 |  | 0.80 | 0.00 | 0.20 |
| SK08_7350 | 2008 | Trawl | Age 0 | 1.00 | 0.00 | 0.00 |  | 0.97 | 0.03 |  | 0.94 | 0.00 | 0.06 |
| SK08_7357 | 2008 | Trawl | Age 0 | 1.00 | 0.00 | 0.00 |  | 0.99 | 0.01 |  | 1.00 | 0.00 | 0.00 |
| SK08_7393 | 2008 | Trawl | Age 0 | 1.00 | 0.00 | 0.00 |  | 0.99 | 0.02 |  | 1.00 | 0.00 | 0.00 |
| SK08_7405 | 2008 | Trawl | Age 0 | 1.00 | 0.00 | 0.00 |  | 0.95 | 0.05 |  | 0.96 | 0.00 | 0.04 |
| SK08_7412 | 2008 | Trawl | Age 0 | 1.00 | 0.00 | 0.00 |  | 0.99 | 0.01 |  | 1.00 | 0.00 | 0.00 |
| SK08_7413 | 2008 | Trawl | Age 0 | 1.00 | 0.00 | 0.00 |  | 0.99 | 0.01 |  | 1.00 | 0.00 | 0.00 |
| SK08_7430 | 2008 | Trawl | Age 0 | 1.00 | 0.00 | 0.00 |  | 0.99 | 0.01 |  | 1.00 | 0.00 | 0.00 |
| SK08_7437 | 2008 | Trawl | Age 0 | 1.00 | 0.00 | 0.00 |  | 0.98 | 0.03 |  | 0.85 | 0.00 | 0.15 |
| SK08_7439 | 2008 | Trawl | Age 0 | 1.00 | 0.00 | 0.00 |  | 0.99 | 0.01 |  | 1.00 | 0.00 | 0.00 |
| SK08_7445 | 2008 | Trawl | Age 0 | 1.00 | 0.00 | 0.00 |  | 0.98 | 0.02 |  | 0.99 | 0.00 | 0.01 |
| SK08_7454 | 2008 | Trawl | Age 0 | 1.00 | 0.00 | 0.00 |  | 0.98 | 0.02 |  | 0.97 | 0.00 | 0.03 |
| SK08_7459 | 2008 | Trawl | Age 0 | 1.00 | 0.00 | 0.00 |  | 0.99 | 0.01 |  | 1.00 | 0.00 | 0.00 |
| SK08_7470 | 2008 | Trawl | Age 0 | 1.00 | 0.00 | 0.00 |  | 0.99 | 0.01 |  | 1.00 | 0.00 | 0.00 |
| SK08_7482 | 2008 | Trawl | Age 0 | 1.00 | 0.00 | 0.00 |  | 0.99 | 0.01 |  | 1.00 | 0.00 | 0.00 |
| SK08_7492 | 2008 | Trawl | Age 0 | 1.00 | 0.00 | 0.00 |  | 0.96 | 0.05 |  | 0.79 | 0.00 | 0.21 |
| SK08_7495 | 2008 | Trawl | Age 0 | 1.00 | 0.00 | 0.00 |  | 0.97 | 0.04 |  | 0.94 | 0.00 | 0.06 |
| SK08_7500 | 2008 | Trawl | Age 0 | 0.92 | 0.00 | 0.08 |  | 0.87 | 0.13 |  | 0.23 | 0.00 | 0.77 |
| SK08_7502 | 2008 | Trawl | Age 0 | 1.00 | 0.00 | 0.00 |  | 0.99 | 0.01 |  | 1.00 | 0.00 | 0.00 |
| SK08_7505 | 2008 | Trawl | Age 0 | 1.00 | 0.00 | 0.00 |  | 0.97 | 0.03 |  | 0.85 | 0.00 | 0.15 |
| SK08_7506 | 2008 | Trawl | Age 0 | 0.00 | 1.00 | 0.00 |  | 0.02 | 0.98 |  | 0.00 | 1.00 | 0.00 |
| SK08_7512 | 2008 | Trawl | Age 0 | 1.00 | 0.00 | 0.00 |  | 0.99 | 0.01 |  | 1.00 | 0.00 | 0.00 |
| SK08_7517 | 2008 | Trawl | Age 0 | 1.00 | 0.00 | 0.00 |  | 0.97 | 0.03 |  | 0.88 | 0.00 | 0.12 |
| SK08_7523 | 2008 | Trawl | Age 0 | 1.00 | 0.00 | 0.00 |  | 0.98 | 0.02 |  | 0.97 | 0.00 | 0.03 |
| SK08_7524 | 2008 | Trawl | Age 0 | 0.10 | 0.51 | 0.39 |  | 0.40 | 0.60 |  | 0.00 | 0.02 | 0.97 |
| SK08_7525 | 2008 | Trawl | Age 0 | 1.00 | 0.00 | 0.00 |  | 0.99 | 0.01 |  | 0.99 | 0.00 | 0.01 |
| SK08_7526 | 2008 | Trawl | Age 0 | 1.00 | 0.00 | 0.00 |  | 0.99 | 0.01 |  | 1.00 | 0.00 | 0.00 |
| SK08_7532 | 2008 | Trawl | Age 0 | 1.00 | 0.00 | 0.00 |  | 0.99 | 0.01 |  | 1.00 | 0.00 | 0.00 |
| SK08_7534 | 2008 | Trawl | Age 0 | 1.00 | 0.00 | 0.00 |  | 0.97 | 0.03 |  | 0.78 | 0.00 | 0.22 |
| SK08_7544 | 2008 | Trawl | Age 0 | 1.00 | 0.00 | 0.00 |  | 0.99 | 0.01 |  | 0.96 | 0.00 | 0.04 |
| SK08_7545 | 2008 | Trawl | Age 0 | 1.00 | 0.00 | 0.00 |  | 0.99 | 0.01 |  | 1.00 | 0.00 | 0.00 |
| SK08_7546 | 2008 | Trawl | Age 0 | 1.00 | 0.00 | 0.00 |  | 0.99 | 0.01 |  | 1.00 | 0.00 | 0.00 |
| SK08_7548 | 2008 | Trawl | Age 0 | 1.00 | 0.00 | 0.00 |  | 0.98 | 0.02 |  | 0.95 | 0.00 | 0.05 |
| SK08_7549 | 2008 | Trawl | Age 0 | 1.00 | 0.00 | 0.00 |  | 0.99 | 0.02 |  | 0.98 | 0.00 | 0.02 |
| SK08_7550 | 2008 | Trawl | Age 0 | 1.00 | 0.00 | 0.00 |  | 0.99 | 0.01 |  | 1.00 | 0.00 | 0.00 |
| SK08_7554 | 2008 | Trawl | Age 0 | 0.99 | 0.00 | 0.01 |  | 0.91 | 0.09 |  | 0.43 | 0.00 | 0.57 |
| SK08_7557 | 2008 | Trawl | Age 0 | 1.00 | 0.00 | 0.00 |  | 0.99 | 0.01 |  | 0.97 | 0.00 | 0.03 |
| SK08_7559 | 2008 | Trawl | Age 0 | 1.00 | 0.00 | 0.00 |  | 0.99 | 0.01 |  | 1.00 | 0.00 | 0.00 |
| SK08_7564 | 2008 | Trawl | Age 0 | 1.00 | 0.00 | 0.00 |  | 0.99 | 0.01 |  | 0.99 | 0.00 | 0.01 |
| SK08_7566 | 2008 | Trawl | Age 0 | 0.96 | 0.00 | 0.04 |  | 0.89 | 0.11 |  | 0.38 | 0.00 | 0.62 |
| SK08_7602 | 2008 | Trawl | Age 0 | 1.00 | 0.00 | 0.00 |  | 0.99 | 0.01 |  | 1.00 | 0.00 | 0.00 |
| SK08_7606 | 2008 | Trawl | Age 0 | 1.00 | 0.00 | 0.00 |  | 0.97 | 0.03 |  | 0.90 | 0.00 | 0.10 |
| SK08_7613 | 2008 | Trawl | Age 0 | 1.00 | 0.00 | 0.00 |  | 0.99 | 0.02 |  | 0.98 | 0.00 | 0.02 |
| SK08_7616 | 2008 | Trawl | Age 0 | 1.00 | 0.00 | 0.00 |  | 0.98 | 0.02 |  | 0.98 | 0.00 | 0.02 |
| SK08_7620 | 2008 | Trawl | Age 0 | 1.00 | 0.00 | 0.00 |  | 0.99 | 0.01 |  | 1.00 | 0.00 | 0.00 |
| SK08_7627 | 2008 | Trawl | Age 0 | 1.00 | 0.00 | 0.00 |  | 0.98 | 0.02 |  | 0.93 | 0.00 | 0.07 |
| SK08_7628 | 2008 | Trawl | Age 0 | 1.00 | 0.00 | 0.00 |  | 0.98 | 0.02 |  | 0.99 | 0.00 | 0.01 |
| SK08_7629 | 2008 | Trawl | Age 0 | 0.86 | 0.00 | 0.13 |  | 0.85 | 0.15 |  | 0.13 | 0.00 | 0.87 |
| SK08_7640 | 2008 | Trawl | Age 0 | 1.00 | 0.00 | 0.00 |  | 0.99 | 0.01 |  | 1.00 | 0.00 | 0.00 |
| SK08_7644 | 2008 | Trawl | Age 0 | 1.00 | 0.00 | 0.00 |  | 0.94 | 0.06 |  | 0.93 | 0.00 | 0.07 |
| SK08_7669 | 2008 | Trawl | Age 0 | 1.00 | 0.00 | 0.00 |  | 0.98 | 0.02 |  | 0.99 | 0.00 | 0.01 |
| SK08_7674 | 2008 | Trawl | Age 0 | 1.00 | 0.00 | 0.00 |  | 0.96 | 0.04 |  | 0.89 | 0.00 | 0.11 |
| SK08_7681 | 2008 | Trawl | Age 0 | 1.00 | 0.00 | 0.00 |  | 0.99 | 0.01 |  | 1.00 | 0.00 | 0.00 |
| SK08_7682 | 2008 | Trawl | Age 0 | 1.00 | 0.00 | 0.00 |  | 0.99 | 0.01 |  | 0.99 | 0.00 | 0.01 |
| SK08_7691 | 2008 | Trawl | Age 0 | 1.00 | 0.00 | 0.00 |  | 0.96 | 0.04 |  | 0.86 | 0.00 | 0.14 |
| SK08_7692 | 2008 | Trawl | Age 0 | 1.00 | 0.00 | 0.00 |  | 0.99 | 0.01 |  | 1.00 | 0.00 | 0.00 |
| SK08_7693 | 2008 | Trawl | Age 0 | 1.00 | 0.00 | 0.00 |  | 0.99 | 0.01 |  | 1.00 | 0.00 | 0.00 |
| SK08_7700 | 2008 | Trawl | Age 0 | 1.00 | 0.00 | 0.00 |  | 0.98 | 0.02 |  | 0.99 | 0.00 | 0.01 |
| SK08_7712 | 2008 | Trawl | Age 0 | 1.00 | 0.00 | 0.00 |  | 0.98 | 0.02 |  | 0.92 | 0.00 | 0.08 |
| SK08_7713 | 2008 | Trawl | Age 0 | 1.00 | 0.00 | 0.00 |  | 0.99 | 0.01 |  | 1.00 | 0.00 | 0.00 |
| SK08_7715 | 2008 | Trawl | Age 0 | 0.99 | 0.00 | 0.01 |  | 0.96 | 0.04 |  | 0.84 | 0.00 | 0.16 |
| SK08_7718 | 2008 | Trawl | Age 0 | 1.00 | 0.00 | 0.00 |  | 0.98 | 0.02 |  | 0.98 | 0.00 | 0.02 |
| SK08_7722 | 2008 | Trawl | Age 0 | 1.00 | 0.00 | 0.00 |  | 0.98 | 0.02 |  | 0.99 | 0.00 | 0.01 |
| SK08_7725 | 2008 | Trawl | Age 0 | 1.00 | 0.00 | 0.00 |  | 0.99 | 0.01 |  | 1.00 | 0.00 | 0.00 |
| SK08_7726 | 2008 | Trawl | Age 0 | 1.00 | 0.00 | 0.00 |  | 1.00 | 0.01 |  | 1.00 | 0.00 | 0.00 |
| SK08_7729 | 2008 | Trawl | Age 0 | 1.00 | 0.00 | 0.00 |  | 0.99 | 0.01 |  | 1.00 | 0.00 | 0.00 |
| SK08_7745 | 2008 | Trawl | Age 0 | 1.00 | 0.00 | 0.00 |  | 0.98 | 0.02 |  | 0.99 | 0.00 | 0.01 |
| SK08_7746 | 2008 | Trawl | Age 0 | 1.00 | 0.00 | 0.00 |  | 0.99 | 0.01 |  | 0.99 | 0.00 | 0.01 |
| SK08_7748 | 2008 | Trawl | Age 0 | 1.00 | 0.00 | 0.00 |  | 0.99 | 0.01 |  | 1.00 | 0.00 | 0.00 |
| SK08_7749 | 2008 | Trawl | Age 0 | 0.99 | 0.00 | 0.01 |  | 0.94 | 0.06 |  | 0.77 | 0.00 | 0.23 |
| SK10-18042 | 2010 | Trawl | Age 0 | 1.00 | 0.00 | 0.00 |  | 0.99 | 0.01 |  | 1.00 | 0.00 | 0.00 |
| SK10-18043 | 2010 | Trawl | Age 0 | 1.00 | 0.00 | 0.00 |  | 0.97 | 0.03 |  | 0.90 | 0.00 | 0.10 |
| SK10-18046 | 2010 | Trawl | Age 0 | 0.98 | 0.00 | 0.02 |  | 0.93 | 0.07 |  | 0.49 | 0.00 | 0.51 |
| SK10-18047 | 2010 | Trawl | Age 0 | 0.99 | 0.00 | 0.01 |  | 0.95 | 0.05 |  | 0.86 | 0.00 | 0.14 |
| SK10-18049 | 2010 | Trawl | Age 0 | 1.00 | 0.00 | 0.00 |  | 0.98 | 0.02 |  | 0.99 | 0.00 | 0.01 |
| SK10-18050 | 2010 | Trawl | Age 0 | 1.00 | 0.00 | 0.00 |  | 0.99 | 0.02 |  | 0.99 | 0.00 | 0.01 |
| SK10-18051 | 2010 | Trawl | Age 0 | 1.00 | 0.00 | 0.00 |  | 0.97 | 0.03 |  | 0.85 | 0.00 | 0.15 |
| SK10-18052 | 2010 | Trawl | Age 0 | 1.00 | 0.00 | 0.00 |  | 0.99 | 0.01 |  | 1.00 | 0.00 | 0.00 |
| SK10-18054 | 2010 | Trawl | Age 0 | 1.00 | 0.00 | 0.00 |  | 0.96 | 0.04 |  | 0.99 | 0.00 | 0.01 |
| SK10-18058 | 2010 | Trawl | Age 0 | 1.00 | 0.00 | 0.00 |  | 0.99 | 0.01 |  | 1.00 | 0.00 | 0.00 |
| SK10-18059 | 2010 | Trawl | Age 0 | 0.98 | 0.00 | 0.02 |  | 0.93 | 0.08 |  | 0.54 | 0.00 | 0.46 |
| SK10-18060 | 2010 | Trawl | Age 0 | 1.00 | 0.00 | 0.00 |  | 0.99 | 0.01 |  | 1.00 | 0.00 | 0.00 |
| SK10-18063 | 2010 | Trawl | Age 0 | 1.00 | 0.00 | 0.00 |  | 0.99 | 0.02 |  | 0.99 | 0.00 | 0.01 |
| SK10-18064 | 2010 | Trawl | Age 0 | 1.00 | 0.00 | 0.00 |  | 0.99 | 0.01 |  | 1.00 | 0.00 | 0.00 |
| SK10-18065 | 2010 | Trawl | Age 0 | 1.00 | 0.00 | 0.00 |  | 0.96 | 0.04 |  | 0.95 | 0.00 | 0.05 |
| SK10-18073 | 2010 | Trawl | Age 0 | 1.00 | 0.00 | 0.00 |  | 0.98 | 0.03 |  | 0.97 | 0.00 | 0.03 |
| SK10-18076 | 2010 | Trawl | Age 0 | 1.00 | 0.00 | 0.00 |  | 0.98 | 0.02 |  | 0.97 | 0.00 | 0.03 |
| SK10-18079 | 2010 | Trawl | Age 0 | 1.00 | 0.00 | 0.00 |  | 0.99 | 0.01 |  | 1.00 | 0.00 | 0.00 |
| SK10-18081 | 2010 | Trawl | Age 0 | 0.99 | 0.00 | 0.01 |  | 0.95 | 0.05 |  | 0.82 | 0.00 | 0.18 |
| SK10-18082 | 2010 | Trawl | Age 0 | 1.00 | 0.00 | 0.00 |  | 0.99 | 0.01 |  | 1.00 | 0.00 | 0.00 |
| SK10-18083 | 2010 | Trawl | Age 0 | 1.00 | 0.00 | 0.00 |  | 0.96 | 0.04 |  | 0.91 | 0.00 | 0.09 |
| SK10-18086 | 2010 | Trawl | Age 0 | 1.00 | 0.00 | 0.00 |  | 0.97 | 0.04 |  | 0.88 | 0.00 | 0.12 |
| SK10-18088 | 2010 | Trawl | Age 0 | 1.00 | 0.00 | 0.00 |  | 0.99 | 0.01 |  | 1.00 | 0.00 | 0.00 |
| SK10-18089 | 2010 | Trawl | Age 0 | 1.00 | 0.00 | 0.00 |  | 0.97 | 0.03 |  | 0.94 | 0.00 | 0.06 |
| SK10-18092 | 2010 | Trawl | Age 0 | 1.00 | 0.00 | 0.00 |  | 0.98 | 0.02 |  | 0.99 | 0.00 | 0.01 |
| SK10-18093 | 2010 | Trawl | Age 0 | 1.00 | 0.00 | 0.00 |  | 0.99 | 0.01 |  | 1.00 | 0.00 | 0.00 |
| SK10-18096 | 2010 | Trawl | Age 0 | 1.00 | 0.00 | 0.00 |  | 0.95 | 0.05 |  | 0.52 | 0.00 | 0.48 |
| SK10-18098 | 2010 | Trawl | Age 0 | 0.40 | 0.00 | 0.60 |  | 0.86 | 0.14 |  | 0.17 | 0.00 | 0.83 |
| SK10-18099 | 2010 | Trawl | Age 0 | 1.00 | 0.00 | 0.00 |  | 0.98 | 0.02 |  | 0.96 | 0.00 | 0.04 |
| SK10-18127 | 2010 | Trawl | Age 0 | 1.00 | 0.00 | 0.00 |  | 0.99 | 0.01 |  | 1.00 | 0.00 | 0.00 |
| SK10-18128 | 2010 | Trawl | Age 0 | 1.00 | 0.00 | 0.00 |  | 0.98 | 0.02 |  | 0.98 | 0.00 | 0.02 |
| SK10-18131 | 2010 | Trawl | Age 0 | 1.00 | 0.00 | 0.00 |  | 0.95 | 0.06 |  | 0.79 | 0.00 | 0.21 |
| SK10-18135 | 2010 | Trawl | Age 0 | 1.00 | 0.00 | 0.00 |  | 0.98 | 0.02 |  | 0.99 | 0.00 | 0.01 |
| SK10-18136 | 2010 | Trawl | Age 0 | 1.00 | 0.00 | 0.00 |  | 0.98 | 0.02 |  | 0.99 | 0.00 | 0.01 |
| SK10-18150 | 2010 | Trawl | Age 0 | 1.00 | 0.00 | 0.00 |  | 0.98 | 0.02 |  | 0.99 | 0.00 | 0.01 |
| SK10-18152 | 2010 | Trawl | Age 0 | 1.00 | 0.00 | 0.00 |  | 0.99 | 0.01 |  | 1.00 | 0.00 | 0.00 |
| SK10-18153 | 2010 | Trawl | Age 0 | 1.00 | 0.00 | 0.00 |  | 0.98 | 0.02 |  | 0.99 | 0.00 | 0.01 |
| SK10-18155 | 2010 | Trawl | Age 0 | 1.00 | 0.00 | 0.00 |  | 0.99 | 0.01 |  | 0.98 | 0.00 | 0.02 |
| SK10-18158 | 2010 | Trawl | Age 0 | 1.00 | 0.00 | 0.00 |  | 0.98 | 0.02 |  | 0.98 | 0.00 | 0.02 |
| SK10-18160 | 2010 | Trawl | Age 0 | 1.00 | 0.00 | 0.00 |  | 0.98 | 0.02 |  | 0.97 | 0.00 | 0.03 |
| SK10-18162 | 2010 | Trawl | Age 0 | 0.97 | 0.00 | 0.03 |  | 0.88 | 0.13 |  | 0.24 | 0.00 | 0.76 |
| SK10-18163 | 2010 | Trawl | Age 0 | 1.00 | 0.00 | 0.00 |  | 0.97 | 0.03 |  | 0.99 | 0.00 | 0.01 |
| SK10-18164 | 2010 | Trawl | Age 0 | 1.00 | 0.00 | 0.00 |  | 0.99 | 0.01 |  | 0.99 | 0.00 | 0.01 |
| SK10-18165 | 2010 | Trawl | Age 0 | 0.77 | 0.00 | 0.23 |  | 0.80 | 0.20 |  | 0.16 | 0.00 | 0.84 |
| SK10-18166 | 2010 | Trawl | Age 0 | 0.99 | 0.00 | 0.01 |  | 0.93 | 0.07 |  | 0.71 | 0.00 | 0.29 |
| SK10-18168 | 2010 | Trawl | Age 0 | 0.99 | 0.00 | 0.01 |  | 0.97 | 0.03 |  | 0.96 | 0.00 | 0.04 |
| SK10-18169 | 2010 | Trawl | Age 0 | 1.00 | 0.00 | 0.00 |  | 0.96 | 0.04 |  | 0.95 | 0.00 | 0.05 |
| SK10-18173 | 2010 | Trawl | Age 0 | 1.00 | 0.00 | 0.00 |  | 0.98 | 0.03 |  | 0.87 | 0.00 | 0.13 |
| SK10-18177 | 2010 | Trawl | Age 0 | 1.00 | 0.00 | 0.00 |  | 0.99 | 0.01 |  | 1.00 | 0.00 | 0.00 |
| SK10-18180 | 2010 | Trawl | Age 0 | 1.00 | 0.00 | 0.00 |  | 0.99 | 0.01 |  | 0.99 | 0.00 | 0.01 |
| SK10-18182 | 2010 | Trawl | Age 0 | 1.00 | 0.00 | 0.00 |  | 0.95 | 0.05 |  | 0.74 | 0.00 | 0.26 |
| SK10-18183 | 2010 | Trawl | Age 0 | 1.00 | 0.00 | 0.00 |  | 0.98 | 0.02 |  | 0.96 | 0.00 | 0.04 |
| SK10-18184 | 2010 | Trawl | Age 0 | 1.00 | 0.00 | 0.00 |  | 0.98 | 0.02 |  | 0.99 | 0.00 | 0.01 |
| SK10-18186 | 2010 | Trawl | Age 0 | 1.00 | 0.00 | 0.00 |  | 0.99 | 0.01 |  | 1.00 | 0.00 | 0.00 |
| SK10-18187 | 2010 | Trawl | Age 0 | 1.00 | 0.00 | 0.00 |  | 0.99 | 0.02 |  | 0.98 | 0.00 | 0.02 |
| SK10-18189 | 2010 | Trawl | Age 0 | 0.99 | 0.00 | 0.01 |  | 0.96 | 0.04 |  | 0.89 | 0.00 | 0.11 |
| SK10-18193 | 2010 | Trawl | Age 0 | 1.00 | 0.00 | 0.00 |  | 0.98 | 0.02 |  | 0.99 | 0.00 | 0.01 |
| SK10-18194 | 2010 | Trawl | Age 0 | 0.08 | 0.02 | 0.90 |  | 0.45 | 0.55 |  | 0.00 | 0.01 | 0.98 |
| SK10-18195 | 2010 | Trawl | Age 0 | 1.00 | 0.00 | 0.00 |  | 0.99 | 0.01 |  | 1.00 | 0.00 | 0.00 |
| SK10-18198 | 2010 | Trawl | Age 0 | 1.00 | 0.00 | 0.00 |  | 0.99 | 0.01 |  | 1.00 | 0.00 | 0.00 |
| SK10-18200 | 2010 | Trawl | Age 0 | 0.58 | 0.00 | 0.42 |  | 0.83 | 0.17 |  | 0.08 | 0.00 | 0.92 |
| SK10-18201 | 2010 | Trawl | Age 0 | 1.00 | 0.00 | 0.00 |  | 0.97 | 0.03 |  | 0.96 | 0.00 | 0.04 |
| SK10-18209 | 2010 | Trawl | Age 0 | 0.97 | 0.00 | 0.03 |  | 0.85 | 0.15 |  | 0.22 | 0.00 | 0.78 |
| SK10-18210 | 2010 | Trawl | Age 0 | 1.00 | 0.00 | 0.00 |  | 0.99 | 0.01 |  | 0.99 | 0.00 | 0.01 |
| SK10-18214 | 2010 | Trawl | Age 0 | 1.00 | 0.00 | 0.00 |  | 0.96 | 0.04 |  | 0.72 | 0.00 | 0.28 |
| SK10-18215 | 2010 | Trawl | Age 0 | 1.00 | 0.00 | 0.00 |  | 0.99 | 0.01 |  | 1.00 | 0.00 | 0.00 |
| SK10-18216 | 2010 | Trawl | Age 0 | 1.00 | 0.00 | 0.00 |  | 0.97 | 0.03 |  | 0.95 | 0.00 | 0.05 |
| SK10-18218 | 2010 | Trawl | Age 0 | 1.00 | 0.00 | 0.00 |  | 0.93 | 0.07 |  | 0.77 | 0.00 | 0.23 |
| SK10-18220 | 2010 | Trawl | Age 0 | 1.00 | 0.00 | 0.00 |  | 0.99 | 0.01 |  | 0.99 | 0.00 | 0.01 |
| SK10-18221 | 2010 | Trawl | Age 0 | 1.00 | 0.00 | 0.00 |  | 0.97 | 0.03 |  | 0.94 | 0.00 | 0.06 |
| SK10-18223 | 2010 | Trawl | Age 0 | 0.99 | 0.00 | 0.01 |  | 0.88 | 0.12 |  | 0.52 | 0.00 | 0.48 |
| SK10-18224 | 2010 | Trawl | Age 0 | 1.00 | 0.00 | 0.00 |  | 0.99 | 0.01 |  | 1.00 | 0.00 | 0.00 |
| SK10-18225 | 2010 | Trawl | Age 0 | 1.00 | 0.00 | 0.00 |  | 0.97 | 0.03 |  | 0.96 | 0.00 | 0.04 |
| SK10-18230 | 2010 | Trawl | Age 0 | 1.00 | 0.00 | 0.00 |  | 0.99 | 0.01 |  | 1.00 | 0.00 | 0.00 |
| SK10-18233 | 2010 | Trawl | Age 0 | 0.87 | 0.00 | 0.13 |  | 0.81 | 0.19 |  | 0.13 | 0.00 | 0.87 |
| SK10-18236 | 2010 | Trawl | Age 0 | 1.00 | 0.00 | 0.00 |  | 0.97 | 0.03 |  | 0.96 | 0.00 | 0.04 |
| SK10-18239 | 2010 | Trawl | Age 0 | 1.00 | 0.00 | 0.00 |  | 0.99 | 0.01 |  | 1.00 | 0.00 | 0.00 |
| SK10-18241 | 2010 | Trawl | Age 0 | 1.00 | 0.00 | 0.00 |  | 0.94 | 0.06 |  | 0.82 | 0.00 | 0.18 |
| SK10-18242 | 2010 | Trawl | Age 0 | 1.00 | 0.00 | 0.00 |  | 0.98 | 0.02 |  | 0.99 | 0.00 | 0.01 |
| SK10-18251 | 2010 | Trawl | Age 0 | 1.00 | 0.00 | 0.00 |  | 0.95 | 0.05 |  | 0.75 | 0.00 | 0.25 |
| SK10-18254 | 2010 | Trawl | Age 0 | 0.98 | 0.00 | 0.02 |  | 0.92 | 0.08 |  | 0.68 | 0.00 | 0.32 |
| SK10-18256 | 2010 | Trawl | Age 0 | 0.94 | 0.00 | 0.06 |  | 0.90 | 0.10 |  | 0.64 | 0.00 | 0.36 |
| SK10-18260 | 2010 | Trawl | Age 0 | 1.00 | 0.00 | 0.00 |  | 0.99 | 0.01 |  | 1.00 | 0.00 | 0.00 |
| SK10-18261 | 2010 | Trawl | Age 0 | 1.00 | 0.00 | 0.00 |  | 0.99 | 0.02 |  | 0.99 | 0.00 | 0.01 |
| SK10-18269 | 2010 | Trawl | Age 0 | 1.00 | 0.00 | 0.00 |  | 0.99 | 0.01 |  | 1.00 | 0.00 | 0.00 |
| SK10-18271 | 2010 | Trawl | Age 0 | 0.83 | 0.00 | 0.17 |  | 0.83 | 0.18 |  | 0.24 | 0.00 | 0.76 |
| SK10-18273 | 2010 | Trawl | Age 0 | 0.98 | 0.00 | 0.02 |  | 0.96 | 0.04 |  | 0.42 | 0.00 | 0.58 |
| SK10-375 | 2010 | Trawl | Age 0 | 1.00 | 0.00 | 0.00 |  | 0.99 | 0.01 |  | 0.99 | 0.00 | 0.01 |
| SK10-381 | 2010 | Trawl | Age 0 | 1.00 | 0.00 | 0.00 |  | 0.99 | 0.01 |  | 1.00 | 0.00 | 0.00 |
| SK10-393 | 2010 | Trawl | Age 0 | 1.00 | 0.00 | 0.00 |  | 0.99 | 0.01 |  | 0.98 | 0.00 | 0.02 |
| SK10-396 | 2010 | Trawl | Age 0 | 1.00 | 0.00 | 0.00 |  | 0.96 | 0.04 |  | 0.85 | 0.00 | 0.15 |
| SK10-399 | 2010 | Trawl | Age 0 | 1.00 | 0.00 | 0.00 |  | 0.99 | 0.01 |  | 1.00 | 0.00 | 0.00 |
| SK10-403 | 2010 | Trawl | Age 0 | 1.00 | 0.00 | 0.00 |  | 0.99 | 0.01 |  | 1.00 | 0.00 | 0.00 |
| SK10-404 | 2010 | Trawl | Age 0 | 1.00 | 0.00 | 0.00 |  | 0.98 | 0.03 |  | 0.96 | 0.00 | 0.04 |
| SK10-414 | 2010 | Trawl | Age 0 | 1.00 | 0.00 | 0.00 |  | 0.99 | 0.01 |  | 1.00 | 0.00 | 0.00 |
| SK10-415 | 2010 | Trawl | Age 0 | 1.00 | 0.00 | 0.00 |  | 0.99 | 0.01 |  | 0.99 | 0.00 | 0.01 |
| SK-12-24143 | 2012 | Trawl | Age 0 | 1.00 | 0.00 | 0.00 |  | 0.93 | 0.07 |  | 0.13 | 0.00 | 0.87 |
| SK-12-24147 | 2012 | Trawl | Age 0 | 1.00 | 0.00 | 0.00 |  | 0.97 | 0.03 |  | 0.50 | 0.00 | 0.50 |
| SK-12-24149 | 2012 | Trawl | Age 0 | 0.00 | 1.00 | 0.00 |  | 0.03 | 0.98 |  | 0.00 | 0.88 | 0.12 |
| SK-12-24150 | 2012 | Trawl | Age 0 | 1.00 | 0.00 | 0.00 |  | 0.97 | 0.03 |  | 0.84 | 0.00 | 0.16 |
| SK-12-24152 | 2012 | Trawl | Age 0 | 1.00 | 0.00 | 0.00 |  | 0.96 | 0.04 |  | 0.58 | 0.00 | 0.42 |
| SK-12-24153 | 2012 | Trawl | Age 0 | 1.00 | 0.00 | 0.00 |  | 0.97 | 0.03 |  | 0.52 | 0.00 | 0.48 |
| SK-12-24154 | 2012 | Trawl | Age 0 | 0.90 | 0.00 | 0.10 |  | 0.70 | 0.30 |  | 0.01 | 0.00 | 0.99 |
| SK-12-24157 | 2012 | Trawl | Age 0 | 1.00 | 0.00 | 0.00 |  | 0.96 | 0.04 |  | 0.45 | 0.00 | 0.55 |
| SK-12-24160 | 2012 | Trawl | Age 0 | 0.71 | 0.00 | 0.29 |  | 0.85 | 0.15 |  | 0.15 | 0.00 | 0.85 |
| SK-12-24161 | 2012 | Trawl | Age 0 | 1.00 | 0.00 | 0.00 |  | 0.98 | 0.02 |  | 0.99 | 0.00 | 0.01 |
| SK-12-24163 | 2012 | Trawl | Age 0 | 1.00 | 0.00 | 0.00 |  | 0.98 | 0.02 |  | 0.99 | 0.00 | 0.01 |
| SK-12-24164 | 2012 | Trawl | Age 0 | 0.90 | 0.00 | 0.10 |  | 0.85 | 0.15 |  | 0.02 | 0.00 | 0.98 |
| SK-12-24165 | 2012 | Trawl | Age 0 | 1.00 | 0.00 | 0.00 |  | 0.98 | 0.02 |  | 0.96 | 0.00 | 0.04 |
| SK-12-24168 | 2012 | Trawl | Age 0 | 1.00 | 0.00 | 0.00 |  | 0.95 | 0.05 |  | 0.55 | 0.00 | 0.45 |
| SK-12-24172 | 2012 | Trawl | Age 0 | 1.00 | 0.00 | 0.00 |  | 0.99 | 0.01 |  | 1.00 | 0.00 | 0.00 |
| SK-12-24176 | 2012 | Trawl | Age 0 | 0.00 | 0.01 | 0.99 |  | 0.34 | 0.66 |  | 0.00 | 0.03 | 0.97 |
| SK-12-24180 | 2012 | Trawl | Age 0 | 1.00 | 0.00 | 0.00 |  | 0.95 | 0.05 |  | 0.94 | 0.00 | 0.06 |
| SK-12-24181 | 2012 | Trawl | Age 0 | 1.00 | 0.00 | 0.00 |  | 0.99 | 0.01 |  | 0.94 | 0.00 | 0.06 |
| SK-12-24184 | 2012 | Trawl | Age 0 | 0.00 | 1.00 | 0.00 |  | 0.01 | 0.99 |  | 0.00 | 0.97 | 0.03 |
| SK-12-24185 | 2012 | Trawl | Age 0 | 1.00 | 0.00 | 0.00 |  | 0.97 | 0.03 |  | 0.85 | 0.00 | 0.15 |
| SK-12-24188 | 2012 | Trawl | Age 0 | 1.00 | 0.00 | 0.00 |  | 0.97 | 0.03 |  | 0.60 | 0.00 | 0.40 |
| SK-12-24189 | 2012 | Trawl | Age 0 | 0.93 | 0.00 | 0.07 |  | 0.84 | 0.17 |  | 0.03 | 0.00 | 0.97 |
| SK-12-24191 | 2012 | Trawl | Age 0 | 0.99 | 0.00 | 0.01 |  | 0.94 | 0.06 |  | 0.35 | 0.00 | 0.65 |
| SK-12-24193 | 2012 | Trawl | Age 0 | 1.00 | 0.00 | 0.00 |  | 0.98 | 0.02 |  | 0.39 | 0.00 | 0.61 |
| SK-12-24194 | 2012 | Trawl | Age 0 | 1.00 | 0.00 | 0.00 |  | 0.99 | 0.01 |  | 1.00 | 0.00 | 0.00 |
| SK-12-24201 | 2012 | Trawl | Age 0 | 1.00 | 0.00 | 0.00 |  | 0.97 | 0.03 |  | 0.91 | 0.00 | 0.09 |
| SK-12-24202 | 2012 | Trawl | Age 0 | 0.97 | 0.00 | 0.03 |  | 0.86 | 0.14 |  | 0.26 | 0.00 | 0.74 |
| SK-12-24203 | 2012 | Trawl | Age 0 | 0.96 | 0.00 | 0.04 |  | 0.92 | 0.08 |  | 0.07 | 0.00 | 0.93 |
| SK-12-24204 | 2012 | Trawl | Age 0 | 0.99 | 0.00 | 0.01 |  | 0.96 | 0.04 |  | 0.23 | 0.00 | 0.77 |
| SK-12-24212 | 2012 | Trawl | Age 0 | 0.00 | 1.00 | 0.00 |  | 0.02 | 0.99 |  | 0.00 | 0.98 | 0.02 |
| SK-12-24216 | 2012 | Trawl | Age 0 | 1.00 | 0.00 | 0.00 |  | 0.97 | 0.03 |  | 0.94 | 0.00 | 0.06 |
| SK-12-24218 | 2012 | Trawl | Age 0 | 1.00 | 0.00 | 0.00 |  | 0.98 | 0.02 |  | 0.94 | 0.00 | 0.06 |
| SK-12-24219 | 2012 | Trawl | Age 0 | 1.00 | 0.00 | 0.00 |  | 0.98 | 0.02 |  | 0.82 | 0.00 | 0.18 |
| SK-12-24221 | 2012 | Trawl | Age 0 | 1.00 | 0.00 | 0.00 |  | 0.99 | 0.01 |  | 0.99 | 0.00 | 0.01 |
| SK-12-24223 | 2012 | Trawl | Age 0 | 1.00 | 0.00 | 0.00 |  | 0.99 | 0.01 |  | 1.00 | 0.00 | 0.00 |
| SK-12-24224 | 2012 | Trawl | Age 0 | 1.00 | 0.00 | 0.00 |  | 0.99 | 0.01 |  | 0.99 | 0.00 | 0.01 |
| SK-12-24226 | 2012 | Trawl | Age 0 | 0.23 | 0.00 | 0.77 |  | 0.67 | 0.33 |  | 0.03 | 0.00 | 0.97 |
| SK-12-24228 | 2012 | Trawl | Age 0 | 0.94 | 0.00 | 0.06 |  | 0.90 | 0.10 |  | 0.06 | 0.00 | 0.94 |
| SK-12-24235 | 2012 | Trawl | Age 0 | 1.00 | 0.00 | 0.00 |  | 0.98 | 0.02 |  | 0.94 | 0.00 | 0.06 |
| SK-12-24237 | 2012 | Trawl | Age 0 | 1.00 | 0.00 | 0.00 |  | 0.98 | 0.02 |  | 0.96 | 0.00 | 0.04 |
| SK-12-24238 | 2012 | Trawl | Age 0 | 1.00 | 0.00 | 0.00 |  | 0.97 | 0.03 |  | 0.93 | 0.00 | 0.07 |
| SK-12-24239 | 2012 | Trawl | Age 0 | 1.00 | 0.00 | 0.00 |  | 0.98 | 0.02 |  | 0.97 | 0.00 | 0.03 |
| SK-12-24244 | 2012 | Trawl | Age 0 | 0.66 | 0.00 | 0.34 |  | 0.82 | 0.18 |  | 0.01 | 0.00 | 0.99 |
| SK-12-24245 | 2012 | Trawl | Age 0 | 1.00 | 0.00 | 0.00 |  | 0.99 | 0.01 |  | 1.00 | 0.00 | 0.00 |
| SK-12-24246 | 2012 | Trawl | Age 0 | 0.99 | 0.00 | 0.01 |  | 0.90 | 0.10 |  | 0.12 | 0.00 | 0.88 |
| SK-12-24248 | 2012 | Trawl | Age 0 | 0.76 | 0.00 | 0.24 |  | 0.77 | 0.23 |  | 0.01 | 0.00 | 0.99 |
| SK-12-24249 | 2012 | Trawl | Age 0 | 0.00 | 1.00 | 0.00 |  | 0.02 | 0.98 |  | 0.00 | 0.95 | 0.05 |
| SK-12-24250 | 2012 | Trawl | Age 0 | 1.00 | 0.00 | 0.00 |  | 0.96 | 0.04 |  | 0.42 | 0.00 | 0.58 |
| SK-12-24251 | 2012 | Trawl | Age 0 | 1.00 | 0.00 | 0.00 |  | 0.99 | 0.01 |  | 1.00 | 0.00 | 0.00 |
| SK-12-24258 | 2012 | Trawl | Age 0 | 1.00 | 0.00 | 0.00 |  | 0.99 | 0.01 |  | 1.00 | 0.00 | 0.00 |
| SK-12-24261 | 2012 | Trawl | Age 0 | 1.00 | 0.00 | 0.00 |  | 0.98 | 0.03 |  | 0.98 | 0.00 | 0.02 |
| SK-12-24264 | 2012 | Trawl | Age 0 | 0.00 | 0.70 | 0.30 |  | 0.10 | 0.90 |  | 0.00 | 0.39 | 0.61 |
| SK-12-24265 | 2012 | Trawl | Age 0 | 0.00 | 0.73 | 0.27 |  | 0.10 | 0.90 |  | 0.00 | 0.22 | 0.78 |
| SK-12-24267 | 2012 | Trawl | Age 0 | 1.00 | 0.00 | 0.00 |  | 0.98 | 0.02 |  | 0.96 | 0.00 | 0.04 |
| SK-12-24268 | 2012 | Trawl | Age 0 | 0.00 | 1.00 | 0.00 |  | 0.02 | 0.99 |  | 0.00 | 0.99 | 0.01 |
| SK-12-24272 | 2012 | Trawl | Age 0 | 0.00 | 1.00 | 0.00 |  | 0.02 | 0.98 |  | 0.00 | 0.98 | 0.02 |
| SK-12-24273 | 2012 | Trawl | Age 0 | 0.00 | 1.00 | 0.00 |  | 0.01 | 0.99 |  | 0.00 | 0.99 | 0.01 |
| SK-12-24274 | 2012 | Trawl | Age 0 | 0.36 | 0.15 | 0.49 |  | 0.41 | 0.59 |  | 0.00 | 0.01 | 0.98 |
| SK-12-24275 | 2012 | Trawl | Age 0 | 0.00 | 1.00 | 0.00 |  | 0.01 | 0.99 |  | 0.00 | 1.00 | 0.00 |
| SK-12-24276 | 2012 | Trawl | Age 0 | 0.06 | 0.00 | 0.94 |  | 0.57 | 0.43 |  | 0.01 | 0.00 | 0.99 |
| SK-12-24278 | 2012 | Trawl | Age 0 | 0.00 | 0.99 | 0.01 |  | 0.04 | 0.96 |  | 0.00 | 0.72 | 0.28 |
| SK-12-24279 | 2012 | Trawl | Age 0 | 0.11 | 0.01 | 0.88 |  | 0.52 | 0.48 |  | 0.01 | 0.00 | 0.99 |
| SK-12-24280 | 2012 | Trawl | Age 0 | 0.00 | 0.93 | 0.07 |  | 0.07 | 0.93 |  | 0.00 | 0.56 | 0.44 |
| SK-12-24282 | 2012 | Trawl | Age 0 | 0.00 | 0.99 | 0.01 |  | 0.05 | 0.95 |  | 0.00 | 0.83 | 0.17 |
| SK-12-24286 | 2012 | Trawl | Age 0 | 0.00 | 0.97 | 0.03 |  | 0.05 | 0.95 |  | 0.00 | 0.68 | 0.32 |
| SK-12-24288 | 2012 | Trawl | Age 0 | 0.00 | 1.00 | 0.00 |  | 0.05 | 0.95 |  | 0.00 | 0.75 | 0.25 |
| SK-12-24290 | 2012 | Trawl | Age 0 | 0.00 | 0.91 | 0.09 |  | 0.06 | 0.94 |  | 0.00 | 0.63 | 0.37 |
| SK-12-24291 | 2012 | Trawl | Age 0 | 0.00 | 0.63 | 0.36 |  | 0.22 | 0.78 |  | 0.00 | 0.14 | 0.86 |
| SK-12-24292 | 2012 | Trawl | Age 0 | 0.00 | 0.98 | 0.02 |  | 0.04 | 0.96 |  | 0.00 | 0.80 | 0.20 |
| SK-12-24294 | 2012 | Trawl | Age 0 | 0.00 | 0.98 | 0.02 |  | 0.04 | 0.96 |  | 0.00 | 0.81 | 0.19 |
| SK-12-24295 | 2012 | Trawl | Age 0 | 0.00 | 1.00 | 0.00 |  | 0.01 | 0.99 |  | 0.00 | 0.98 | 0.02 |
| SK-12-24300 | 2012 | Trawl | Age 0 | 0.00 | 0.98 | 0.02 |  | 0.08 | 0.92 |  | 0.00 | 0.45 | 0.55 |
| SK-12-24301 | 2012 | Trawl | Age 0 | 0.93 | 0.00 | 0.07 |  | 0.91 | 0.09 |  | 0.11 | 0.00 | 0.89 |
| SK-12-24302 | 2012 | Trawl | Age 0 | 0.96 | 0.00 | 0.04 |  | 0.89 | 0.11 |  | 0.15 | 0.00 | 0.85 |
| SK-12-24305 | 2012 | Trawl | Age 0 | 1.00 | 0.00 | 0.00 |  | 0.99 | 0.02 |  | 0.98 | 0.00 | 0.02 |
| SK-12-24306 | 2012 | Trawl | Age 0 | 0.00 | 1.00 | 0.00 |  | 0.02 | 0.98 |  | 0.00 | 0.94 | 0.06 |
| SK-12-24308 | 2012 | Trawl | Age 0 | 0.01 | 0.22 | 0.78 |  | 0.21 | 0.79 |  | 0.00 | 0.04 | 0.96 |
| SK-12-24316 | 2012 | Trawl | Age 0 | 1.00 | 0.00 | 0.00 |  | 0.97 | 0.03 |  | 0.72 | 0.00 | 0.28 |
| SK-12-24322 | 2012 | Trawl | Age 0 | 1.00 | 0.00 | 0.00 |  | 0.98 | 0.02 |  | 0.99 | 0.00 | 0.01 |
| SK-12-24323 | 2012 | Trawl | Age 0 | 0.00 | 1.00 | 0.00 |  | 0.02 | 0.99 |  | 0.00 | 0.98 | 0.02 |
| SK-12-24324 | 2012 | Trawl | Age 0 | 0.00 | 1.00 | 0.00 |  | 0.01 | 0.99 |  | 0.00 | 1.00 | 0.00 |
| SK-12-24326 | 2012 | Trawl | Age 0 | 1.00 | 0.00 | 0.00 |  | 0.95 | 0.05 |  | 0.37 | 0.00 | 0.63 |
| SK-12-24333 | 2012 | Trawl | Age 0 | 1.00 | 0.00 | 0.00 |  | 0.99 | 0.01 |  | 0.99 | 0.00 | 0.01 |
| SK-12-24335 | 2012 | Trawl | Age 0 | 0.08 | 0.00 | 0.92 |  | 0.52 | 0.48 |  | 0.00 | 0.01 | 0.99 |
| SK-12-24336 | 2012 | Trawl | Age 0 | 0.99 | 0.00 | 0.01 |  | 0.92 | 0.08 |  | 0.35 | 0.00 | 0.65 |
| SK-12-24340 | 2012 | Trawl | Age 0 | 0.00 | 0.56 | 0.44 |  | 0.13 | 0.87 |  | 0.00 | 0.14 | 0.86 |
| SK-12-24341 | 2012 | Trawl | Age 0 | 0.00 | 0.88 | 0.12 |  | 0.04 | 0.96 |  | 0.00 | 0.70 | 0.30 |
| SK-12-24342 | 2012 | Trawl | Age 0 | 0.00 | 1.00 | 0.00 |  | 0.01 | 0.99 |  | 0.00 | 0.99 | 0.01 |
| SK-12-24351 | 2012 | Trawl | Age 0 | 1.00 | 0.00 | 0.00 |  | 0.99 | 0.01 |  | 0.98 | 0.00 | 0.02 |
| SK-12-24353 | 2012 | Trawl | Age 0 | 1.00 | 0.00 | 0.00 |  | 0.99 | 0.02 |  | 0.99 | 0.00 | 0.01 |
| SK-12-24355 | 2012 | Trawl | Age 0 | 0.98 | 0.00 | 0.02 |  | 0.84 | 0.16 |  | 0.38 | 0.00 | 0.62 |
| SK-12-24358 | 2012 | Trawl | Age 0 | 1.00 | 0.00 | 0.00 |  | 0.99 | 0.01 |  | 0.99 | 0.00 | 0.01 |
| SK-12-24360 | 2012 | Trawl | Age 0 | 1.00 | 0.00 | 0.00 |  | 0.98 | 0.02 |  | 0.78 | 0.00 | 0.22 |
| SK-12-24361 | 2012 | Trawl | Age 0 | 1.00 | 0.00 | 0.00 |  | 0.99 | 0.01 |  | 0.99 | 0.00 | 0.01 |
| SK-14-34504 | 2014 | Trawl | Age 0 | 0.00 | 1.00 | 0.00 |  | 0.01 | 0.99 |  | 0.00 | 1.00 | 0.00 |
| SK-14-34509 | 2014 | Trawl | Age 0 | 0.00 | 1.00 | 0.00 |  | 0.02 | 0.98 |  | 0.00 | 0.98 | 0.02 |
| SK-14-34512 | 2014 | Trawl | Age 0 | 0.06 | 0.00 | 0.94 |  | 0.55 | 0.45 |  | 0.00 | 0.00 | 1.00 |
| SK-14-34519 | 2014 | Trawl | Age 0 | 1.00 | 0.00 | 0.00 |  | 0.99 | 0.01 |  | 1.00 | 0.00 | 0.00 |
| SK-14-34521 | 2014 | Trawl | Age 0 | 0.00 | 1.00 | 0.00 |  | 0.02 | 0.98 |  | 0.00 | 0.98 | 0.02 |
| SK-14-34524 | 2014 | Trawl | Age 0 | 1.00 | 0.00 | 0.00 |  | 0.97 | 0.03 |  | 0.89 | 0.00 | 0.11 |
| SK-14-34810 | 2014 | Trawl | Age 0 | 0.00 | 1.00 | 0.00 |  | 0.01 | 0.99 |  | 0.00 | 1.00 | 0.00 |
| SK-14-34811 | 2014 | Trawl | Age 0 | 0.00 | 1.00 | 0.00 |  | 0.03 | 0.98 |  | 0.00 | 0.95 | 0.05 |
| SK-14-34813 | 2014 | Trawl | Age 0 | 1.00 | 0.00 | 0.00 |  | 0.99 | 0.01 |  | 1.00 | 0.00 | 0.00 |
| SK-14-34817 | 2014 | Trawl | Age 0 | 0.00 | 0.27 | 0.73 |  | 0.18 | 0.82 |  | 0.00 | 0.12 | 0.88 |
| SK-14-34819 | 2014 | Trawl | Age 0 | 0.00 | 1.00 | 0.00 |  | 0.01 | 0.99 |  | 0.00 | 1.00 | 0.00 |
| SK-14-34825 | 2014 | Trawl | Age 0 | 0.00 | 0.92 | 0.08 |  | 0.04 | 0.96 |  | 0.00 | 0.93 | 0.07 |
| SK-14-34826 | 2014 | Trawl | Age 0 | 0.47 | 0.00 | 0.53 |  | 0.73 | 0.27 |  | 0.06 | 0.00 | 0.94 |
| SK-14-34828 | 2014 | Trawl | Age 0 | 0.61 | 0.00 | 0.39 |  | 0.77 | 0.23 |  | 0.11 | 0.00 | 0.89 |
| SK-14-34832 | 2014 | Trawl | Age 0 | 1.00 | 0.00 | 0.00 |  | 0.98 | 0.02 |  | 0.99 | 0.00 | 0.01 |
| SK-14-34834 | 2014 | Trawl | Age 0 | 1.00 | 0.00 | 0.00 |  | 0.99 | 0.01 |  | 1.00 | 0.00 | 0.00 |
| SK-14-34839 | 2014 | Trawl | Age 0 | 1.00 | 0.00 | 0.00 |  | 0.97 | 0.03 |  | 0.96 | 0.00 | 0.04 |
| SK-14-34842 | 2014 | Trawl | Age 0 | 1.00 | 0.00 | 0.00 |  | 0.93 | 0.07 |  | 0.83 | 0.00 | 0.17 |
| SK-14-34843 | 2014 | Trawl | Age 0 | 1.00 | 0.00 | 0.00 |  | 0.99 | 0.01 |  | 1.00 | 0.00 | 0.00 |
| SK-14-34858 | 2014 | Trawl | Age 0 | 0.99 | 0.00 | 0.01 |  | 0.95 | 0.05 |  | 0.85 | 0.00 | 0.15 |
| SK-14-34861 | 2014 | Trawl | Age 0 | 1.00 | 0.00 | 0.00 |  | 0.99 | 0.02 |  | 0.99 | 0.00 | 0.01 |
| SK-14-34862 | 2014 | Trawl | Age 0 | 1.00 | 0.00 | 0.00 |  | 0.99 | 0.01 |  | 1.00 | 0.00 | 0.00 |
| SK-14-34876 | 2014 | Trawl | Age 0 | 1.00 | 0.00 | 0.00 |  | 1.00 | 0.00 |  | 1.00 | 0.00 | 0.00 |
| SK-14-34881 | 2014 | Trawl | Age 0 | 1.00 | 0.00 | 0.00 |  | 0.99 | 0.01 |  | 1.00 | 0.00 | 0.00 |
| SK-14-34883 | 2014 | Trawl | Age 0 | 1.00 | 0.00 | 0.00 |  | 0.99 | 0.01 |  | 1.00 | 0.00 | 0.00 |
| SK-14-34884 | 2014 | Trawl | Age 0 | 0.01 | 0.08 | 0.91 |  | 0.27 | 0.73 |  | 0.00 | 0.09 | 0.91 |
| SK-14-34887 | 2014 | Trawl | Age 0 | 1.00 | 0.00 | 0.00 |  | 0.98 | 0.02 |  | 0.99 | 0.00 | 0.01 |
| SK-14-34898 | 2014 | Trawl | Age 0 | 0.00 | 1.00 | 0.00 |  | 0.01 | 0.99 |  | 0.00 | 1.00 | 0.00 |
| SK-14-34904 | 2014 | Trawl | Age 0 | 0.00 | 1.00 | 0.00 |  | 0.01 | 0.99 |  | 0.00 | 1.00 | 0.00 |
| SK-14-34910 | 2014 | Trawl | Age 0 | 0.00 | 1.00 | 0.00 |  | 0.01 | 0.99 |  | 0.00 | 1.00 | 0.00 |
| SK-14-34911 | 2014 | Trawl | Age 0 | 0.00 | 0.99 | 0.01 |  | 0.04 | 0.97 |  | 0.00 | 0.93 | 0.07 |
| SK-14-34915 | 2014 | Trawl | Age 0 | 0.00 | 1.00 | 0.00 |  | 0.02 | 0.99 |  | 0.00 | 1.00 | 0.00 |
| SK-14-34916 | 2014 | Trawl | Age 0 | 0.00 | 0.99 | 0.01 |  | 0.05 | 0.95 |  | 0.00 | 0.81 | 0.19 |
| SK-14-34917 | 2014 | Trawl | Age 0 | 0.00 | 1.00 | 0.00 |  | 0.01 | 0.99 |  | 0.00 | 1.00 | 0.00 |
| SK-14-34924 | 2014 | Trawl | Age 0 | 0.00 | 1.00 | 0.00 |  | 0.01 | 0.99 |  | 0.00 | 1.00 | 0.00 |
| SK-14-34928 | 2014 | Trawl | Age 0 | 0.00 | 1.00 | 0.00 |  | 0.02 | 0.98 |  | 0.00 | 0.94 | 0.06 |
| SK-14-34933 | 2014 | Trawl | Age 0 | 0.00 | 1.00 | 0.00 |  | 0.01 | 0.99 |  | 0.00 | 1.00 | 0.00 |
| SK-14-34936 | 2014 | Trawl | Age 0 | 1.00 | 0.00 | 0.00 |  | 0.98 | 0.02 |  | 0.89 | 0.00 | 0.11 |
| SK-14-34937 | 2014 | Trawl | Age 0 | 0.86 | 0.00 | 0.14 |  | 0.72 | 0.28 |  | 0.04 | 0.00 | 0.96 |
| SK-14-34938 | 2014 | Trawl | Age 0 | 1.00 | 0.00 | 0.00 |  | 0.99 | 0.01 |  | 1.00 | 0.00 | 0.00 |
| SK-14-34939 | 2014 | Trawl | Age 0 | 0.00 | 1.00 | 0.00 |  | 0.01 | 0.99 |  | 0.00 | 1.00 | 0.00 |
| SK-14-34943 | 2014 | Trawl | Age 0 | 0.00 | 1.00 | 0.00 |  | 0.03 | 0.97 |  | 0.00 | 0.91 | 0.09 |
| SK-14-34955 | 2014 | Trawl | Age 0 | 0.00 | 1.00 | 0.00 |  | 0.01 | 0.99 |  | 0.00 | 1.00 | 0.00 |
| SK-14-34956 | 2014 | Trawl | Age 0 | 0.10 | 0.00 | 0.90 |  | 0.57 | 0.43 |  | 0.01 | 0.00 | 0.99 |
| SK-14-34961 | 2014 | Trawl | Age 0 | 0.00 | 0.98 | 0.02 |  | 0.02 | 0.98 |  | 0.00 | 0.95 | 0.05 |
| SK-14-34971 | 2014 | Trawl | Age 0 | 0.00 | 1.00 | 0.00 |  | 0.01 | 0.99 |  | 0.00 | 1.00 | 0.00 |
| SK-14-34973 | 2014 | Trawl | Age 0 | 0.91 | 0.00 | 0.09 |  | 0.83 | 0.17 |  | 0.22 | 0.00 | 0.78 |
| SK-14-34975 | 2014 | Trawl | Age 0 | 0.06 | 0.00 | 0.94 |  | 0.60 | 0.41 |  | 0.00 | 0.00 | 1.00 |
| SK-14-34980 | 2014 | Trawl | Age 0 | 0.00 | 1.00 | 0.00 |  | 0.03 | 0.97 |  | 0.00 | 0.96 | 0.04 |
| SK-14-34983 | 2014 | Trawl | Age 0 | 0.91 | 0.00 | 0.09 |  | 0.76 | 0.24 |  | 0.02 | 0.00 | 0.98 |
| SK-14-34984 | 2014 | Trawl | Age 0 | 0.00 | 1.00 | 0.00 |  | 0.02 | 0.98 |  | 0.00 | 0.97 | 0.03 |
| SK-14-34986 | 2014 | Trawl | Age 0 | 0.00 | 1.00 | 0.00 |  | 0.02 | 0.98 |  | 0.00 | 0.99 | 0.01 |
| SK-14-34987 | 2014 | Trawl | Age 0 | 0.00 | 1.00 | 0.00 |  | 0.36 | 0.65 |  | 0.00 | 0.99 | 0.01 |
| SK-14-34988 | 2014 | Trawl | Age 0 | 0.00 | 1.00 | 0.00 |  | 0.02 | 0.98 |  | 0.00 | 0.97 | 0.03 |
| SK-14-34999 | 2014 | Trawl | Age 0 | 0.00 | 1.00 | 0.00 |  | 0.01 | 0.99 |  | 0.00 | 1.00 | 0.00 |
| SK-14-35028 | 2014 | Trawl | Age 0 | 0.00 | 1.00 | 0.00 |  | 0.01 | 0.99 |  | 0.00 | 1.00 | 0.00 |
| SK-14-35030 | 2014 | Trawl | Age 0 | 0.00 | 1.00 | 0.00 |  | 0.01 | 0.99 |  | 0.00 | 1.00 | 0.00 |
| SK-14-35035 | 2014 | Trawl | Age 0 | 1.00 | 0.00 | 0.00 |  | 0.97 | 0.03 |  | 0.94 | 0.00 | 0.06 |
| SK-14-35042 | 2014 | Trawl | Age 0 | 1.00 | 0.00 | 0.00 |  | 0.99 | 0.01 |  | 1.00 | 0.00 | 0.00 |
| SK-14-35045 | 2014 | Trawl | Age 0 | 1.00 | 0.00 | 0.00 |  | 0.98 | 0.02 |  | 0.98 | 0.00 | 0.02 |
| SK-14-35052 | 2014 | Trawl | Age 0 | 0.97 | 0.00 | 0.03 |  | 0.91 | 0.09 |  | 0.40 | 0.00 | 0.60 |
| SK-14-35053 | 2014 | Trawl | Age 0 | 0.98 | 0.00 | 0.02 |  | 0.92 | 0.09 |  | 0.52 | 0.00 | 0.48 |
| SK-14-35056 | 2014 | Trawl | Age 0 | 1.00 | 0.00 | 0.00 |  | 0.92 | 0.08 |  | 0.72 | 0.00 | 0.28 |
| SK-14-35060 | 2014 | Trawl | Age 0 | 0.00 | 1.00 | 0.00 |  | 0.07 | 0.93 |  | 0.00 | 1.00 | 0.00 |
| SK-14-35070 | 2014 | Trawl | Age 0 | 0.00 | 1.00 | 0.00 |  | 0.02 | 0.99 |  | 0.00 | 1.00 | 0.00 |
| SK-14-35079 | 2014 | Trawl | Age 0 | 1.00 | 0.00 | 0.00 |  | 0.98 | 0.02 |  | 0.97 | 0.00 | 0.03 |
| SK-14-35083 | 2014 | Trawl | Age 0 | 0.72 | 0.00 | 0.28 |  | 0.76 | 0.25 |  | 0.14 | 0.00 | 0.86 |
| SK-14-35087 | 2014 | Trawl | Age 0 | 1.00 | 0.00 | 0.00 |  | 0.97 | 0.03 |  | 0.94 | 0.00 | 0.06 |
| SK-14-35091 | 2014 | Trawl | Age 0 | 0.00 | 0.99 | 0.01 |  | 0.02 | 0.98 |  | 0.00 | 0.96 | 0.04 |
| SK-14-35096 | 2014 | Trawl | Age 0 | 0.00 | 0.85 | 0.15 |  | 0.08 | 0.92 |  | 0.00 | 0.72 | 0.28 |
| SK-14-35097 | 2014 | Trawl | Age 0 | 0.00 | 1.00 | 0.00 |  | 0.07 | 0.93 |  | 0.00 | 1.00 | 0.00 |
| SK-14-35107 | 2014 | Trawl | Age 0 | 1.00 | 0.00 | 0.00 |  | 0.99 | 0.01 |  | 1.00 | 0.00 | 0.00 |
| SK-14-35116 | 2014 | Trawl | Age 0 | 0.50 | 0.00 | 0.50 |  | 0.80 | 0.20 |  | 0.08 | 0.00 | 0.92 |
| SK-14-35118 | 2014 | Trawl | Age 0 | 0.09 | 0.32 | 0.59 |  | 0.29 | 0.71 |  | 0.00 | 0.10 | 0.90 |
| SK-14-35121 | 2014 | Trawl | Age 0 | 0.00 | 0.99 | 0.01 |  | 0.03 | 0.98 |  | 0.00 | 0.98 | 0.02 |
| SK-14-35131 | 2014 | Trawl | Age 0 | 1.00 | 0.00 | 0.00 |  | 0.99 | 0.01 |  | 1.00 | 0.00 | 0.00 |
| SK-14-35137 | 2014 | Trawl | Age 0 | 0.02 | 0.00 | 0.98 |  | 0.49 | 0.51 |  | 0.00 | 0.01 | 0.99 |
| SK-14-35138 | 2014 | Trawl | Age 0 | 1.00 | 0.00 | 0.00 |  | 0.99 | 0.01 |  | 1.00 | 0.00 | 0.00 |
| SK-14-35143 | 2014 | Trawl | Age 0 | 1.00 | 0.00 | 0.00 |  | 0.98 | 0.03 |  | 0.99 | 0.00 | 0.01 |
| SK-14-35144 | 2014 | Trawl | Age 0 | 0.84 | 0.01 | 0.15 |  | 0.73 | 0.27 |  | 0.04 | 0.00 | 0.95 |
| SK-14-35150 | 2014 | Trawl | Age 0 | 1.00 | 0.00 | 0.00 |  | 0.98 | 0.02 |  | 0.99 | 0.00 | 0.01 |
| SK-14-35155 | 2014 | Trawl | Age 0 | 0.96 | 0.00 | 0.04 |  | 0.85 | 0.15 |  | 0.35 | 0.00 | 0.65 |
| SK-14-35158 | 2014 | Trawl | Age 0 | 1.00 | 0.00 | 0.00 |  | 0.99 | 0.01 |  | 1.00 | 0.00 | 0.00 |
| SK-14-35160 | 2014 | Trawl | Age 0 | 0.00 | 1.00 | 0.00 |  | 0.01 | 0.99 |  | 0.00 | 1.00 | 0.00 |
| SK-14-35162 | 2014 | Trawl | Age 0 | 1.00 | 0.00 | 0.00 |  | 0.97 | 0.03 |  | 0.98 | 0.00 | 0.02 |
| SK-14-35172 | 2014 | Trawl | Age 0 | 1.00 | 0.00 | 0.00 |  | 0.99 | 0.01 |  | 1.00 | 0.00 | 0.00 |
| SK-14-35178 | 2014 | Trawl | Age 0 | 1.00 | 0.00 | 0.00 |  | 0.97 | 0.03 |  | 0.98 | 0.00 | 0.02 |
| SK-14-35182 | 2014 | Trawl | Age 0 | 1.00 | 0.00 | 0.00 |  | 0.99 | 0.02 |  | 0.99 | 0.00 | 0.01 |
| SK-14-35183 | 2014 | Trawl | Age 0 | 1.00 | 0.00 | 0.00 |  | 0.98 | 0.02 |  | 0.98 | 0.00 | 0.02 |
| SK-14-35184 | 2014 | Trawl | Age 0 | 1.00 | 0.00 | 0.00 |  | 0.98 | 0.02 |  | 0.98 | 0.00 | 0.02 |
| SK-14-35191 | 2014 | Trawl | Age 0 | 1.00 | 0.00 | 0.00 |  | 0.96 | 0.04 |  | 0.91 | 0.00 | 0.09 |
| SK-13-age1-26447 | 2013 | Trawl | Age 1 & 2 | 1.00 | 0.00 | 0.00 |  | 0.99 | 0.01 |  | 1.00 | 0.00 | 0.00 |
| SK-13-age1-26450 | 2013 | Trawl | Age 1 & 2 | 1.00 | 0.00 | 0.00 |  | 0.98 | 0.02 |  | 0.98 | 0.00 | 0.02 |
| SK-13-age1-26982 | 2013 | Trawl | Age 1 & 2 | 1.00 | 0.00 | 0.00 |  | 0.99 | 0.01 |  | 0.99 | 0.00 | 0.01 |
| SK-13-age1-26983 | 2013 | Trawl | Age 1 & 2 | 0.98 | 0.00 | 0.02 |  | 0.75 | 0.25 |  | 0.04 | 0.00 | 0.96 |
| SK-13-age1-26999 | 2013 | Trawl | Age 1 & 2 | 1.00 | 0.00 | 0.00 |  | 0.98 | 0.02 |  | 0.99 | 0.00 | 0.01 |
| SK-13-age1-27000 | 2013 | Trawl | Age 1 & 2 | 1.00 | 0.00 | 0.00 |  | 0.97 | 0.03 |  | 0.96 | 0.00 | 0.04 |
| SK-13-age1-27378 | 2013 | Trawl | Age 1 & 2 | 1.00 | 0.00 | 0.00 |  | 0.99 | 0.01 |  | 0.99 | 0.00 | 0.01 |
| SK-13-age1-27381 | 2013 | Trawl | Age 1 & 2 | 1.00 | 0.00 | 0.00 |  | 0.98 | 0.02 |  | 0.99 | 0.00 | 0.01 |
| SK-13-age1-27382 | 2013 | Trawl | Age 1 & 2 | 0.97 | 0.00 | 0.03 |  | 0.91 | 0.09 |  | 0.28 | 0.00 | 0.72 |
| SK-13-age1-27383 | 2013 | Trawl | Age 1 & 2 | 0.28 | 0.01 | 0.71 |  | 0.56 | 0.44 |  | 0.01 | 0.00 | 0.99 |
| SK-13-age1-27387 | 2013 | Trawl | Age 1 & 2 | 1.00 | 0.00 | 0.00 |  | 0.98 | 0.02 |  | 0.99 | 0.00 | 0.01 |
| SK-13-age1-27393 | 2013 | Trawl | Age 1 & 2 | 1.00 | 0.00 | 0.00 |  | 0.99 | 0.01 |  | 1.00 | 0.00 | 0.00 |
| SK-13-age1-27395 | 2013 | Trawl | Age 1 & 2 | 1.00 | 0.00 | 0.00 |  | 0.99 | 0.01 |  | 1.00 | 0.00 | 0.00 |
| SK-13-age1-27413 | 2013 | Trawl | Age 1 & 2 | 1.00 | 0.00 | 0.00 |  | 0.95 | 0.05 |  | 0.70 | 0.00 | 0.30 |
| SK-13-age1-27414 | 2013 | Trawl | Age 1 & 2 | 0.96 | 0.00 | 0.04 |  | 0.87 | 0.13 |  | 0.47 | 0.00 | 0.53 |
| SK-13-age1-27419 | 2013 | Trawl | Age 1 & 2 | 0.91 | 0.00 | 0.09 |  | 0.85 | 0.15 |  | 0.20 | 0.00 | 0.80 |
| SK-13-age1-27420 | 2013 | Trawl | Age 1 & 2 | 1.00 | 0.00 | 0.00 |  | 0.96 | 0.04 |  | 0.87 | 0.00 | 0.13 |
| SK-13-age1-27433 | 2013 | Trawl | Age 1 & 2 | 0.93 | 0.00 | 0.06 |  | 0.73 | 0.27 |  | 0.11 | 0.00 | 0.89 |
| SK-13-age1-27434 | 2013 | Trawl | Age 1 & 2 | 1.00 | 0.00 | 0.00 |  | 0.99 | 0.01 |  | 1.00 | 0.00 | 0.00 |
| SK-13-age1-27446 | 2013 | Trawl | Age 1 & 2 | 0.50 | 0.00 | 0.50 |  | 0.70 | 0.30 |  | 0.01 | 0.00 | 0.99 |
| SK-13-age1-27447 | 2013 | Trawl | Age 1 & 2 | 1.00 | 0.00 | 0.00 |  | 0.99 | 0.01 |  | 1.00 | 0.00 | 0.00 |
| SK-13-age1-27454 | 2013 | Trawl | Age 1 & 2 | 1.00 | 0.00 | 0.00 |  | 0.98 | 0.03 |  | 0.97 | 0.00 | 0.03 |
| SK-13-age1-27468 | 2013 | Trawl | Age 1 & 2 | 0.99 | 0.00 | 0.01 |  | 0.94 | 0.06 |  | 0.78 | 0.00 | 0.22 |
| SK-13-age1-27471 | 2013 | Trawl | Age 1 & 2 | 1.00 | 0.00 | 0.00 |  | 0.99 | 0.01 |  | 1.00 | 0.00 | 0.00 |
| SK-13-age1-27476 | 2013 | Trawl | Age 1 & 2 | 0.99 | 0.00 | 0.01 |  | 0.93 | 0.07 |  | 0.70 | 0.00 | 0.30 |
| SK-13-age1-27486 | 2013 | Trawl | Age 1 & 2 | 1.00 | 0.00 | 0.00 |  | 0.99 | 0.01 |  | 1.00 | 0.00 | 0.00 |
| SK-13-age1-27487 | 2013 | Trawl | Age 1 & 2 | 1.00 | 0.00 | 0.00 |  | 0.99 | 0.01 |  | 1.00 | 0.00 | 0.00 |
| SK-13-age1-27491 | 2013 | Trawl | Age 1 & 2 | 1.00 | 0.00 | 0.00 |  | 0.98 | 0.02 |  | 0.97 | 0.00 | 0.03 |
| SK-13-age1-27497 | 2013 | Trawl | Age 1 & 2 | 1.00 | 0.00 | 0.00 |  | 0.99 | 0.01 |  | 1.00 | 0.00 | 0.00 |
| SK-13-age1-27500 | 2013 | Trawl | Age 1 & 2 | 1.00 | 0.00 | 0.00 |  | 0.99 | 0.01 |  | 0.99 | 0.00 | 0.01 |
| SK-13-age1-27510 | 2013 | Trawl | Age 1 & 2 | 1.00 | 0.00 | 0.00 |  | 0.97 | 0.03 |  | 0.95 | 0.00 | 0.05 |
| SK-13-age1-27515 | 2013 | Trawl | Age 1 & 2 | 0.00 | 1.00 | 0.00 |  | 0.01 | 0.99 |  | 0.00 | 1.00 | 0.00 |
| SK-13-age1-27518 | 2013 | Trawl | Age 1 & 2 | 1.00 | 0.00 | 0.00 |  | 0.98 | 0.02 |  | 0.98 | 0.00 | 0.02 |
| SK-13-age1-27521 | 2013 | Trawl | Age 1 & 2 | 1.00 | 0.00 | 0.00 |  | 0.99 | 0.02 |  | 1.00 | 0.00 | 0.00 |
| SK-13-age1-27522 | 2013 | Trawl | Age 1 & 2 | 1.00 | 0.00 | 0.00 |  | 1.00 | 0.00 |  | 1.00 | 0.00 | 0.00 |
| SK-13-age1-27526 | 2013 | Trawl | Age 1 & 2 | 1.00 | 0.00 | 0.00 |  | 1.00 | 0.01 |  | 1.00 | 0.00 | 0.00 |
| SK-13-age1-27527 | 2013 | Trawl | Age 1 & 2 | 1.00 | 0.00 | 0.00 |  | 1.00 | 0.01 |  | 1.00 | 0.00 | 0.00 |
| SK-13-age1-27532 | 2013 | Trawl | Age 1 & 2 | 0.98 | 0.00 | 0.02 |  | 0.96 | 0.05 |  | 0.93 | 0.00 | 0.07 |
| SK-13-age1-27540 | 2013 | Trawl | Age 1 & 2 | 0.00 | 0.03 | 0.97 |  | 0.25 | 0.75 |  | 0.00 | 0.02 | 0.98 |
| SK-13-age1-27547 | 2013 | Trawl | Age 1 & 2 | 1.00 | 0.00 | 0.00 |  | 0.94 | 0.06 |  | 0.58 | 0.00 | 0.42 |
| SK-13-age1-27571 | 2013 | Trawl | Age 1 & 2 | 1.00 | 0.00 | 0.00 |  | 0.99 | 0.01 |  | 1.00 | 0.00 | 0.00 |
| SK-13-age1-27590 | 2013 | Trawl | Age 1 & 2 | 1.00 | 0.00 | 0.00 |  | 0.99 | 0.02 |  | 0.99 | 0.00 | 0.01 |
| SK-13-age1-27604 | 2013 | Trawl | Age 1 & 2 | 0.90 | 0.00 | 0.10 |  | 0.92 | 0.08 |  | 0.30 | 0.00 | 0.70 |
| SK-13-age1-27605 | 2013 | Trawl | Age 1 & 2 | 1.00 | 0.00 | 0.00 |  | 0.98 | 0.02 |  | 0.87 | 0.00 | 0.13 |
| SK-13-age1-27607 | 2013 | Trawl | Age 1 & 2 | 1.00 | 0.00 | 0.00 |  | 0.98 | 0.02 |  | 0.96 | 0.00 | 0.04 |
| SK-13-age1-27610 | 2013 | Trawl | Age 1 & 2 | 1.00 | 0.00 | 0.00 |  | 0.99 | 0.01 |  | 1.00 | 0.00 | 0.00 |
| SK-13-age1-27612 | 2013 | Trawl | Age 1 & 2 | 0.99 | 0.00 | 0.01 |  | 0.94 | 0.06 |  | 0.59 | 0.00 | 0.41 |
| SK-13-age1-27619 | 2013 | Trawl | Age 1 & 2 | 1.00 | 0.00 | 0.00 |  | 0.99 | 0.01 |  | 1.00 | 0.00 | 0.00 |
| SK-13-age1-27624 | 2013 | Trawl | Age 1 & 2 | 1.00 | 0.00 | 0.00 |  | 0.99 | 0.01 |  | 1.00 | 0.00 | 0.00 |
| SK-13-age1-27630 | 2013 | Trawl | Age 1 & 2 | 1.00 | 0.00 | 0.00 |  | 0.98 | 0.02 |  | 0.99 | 0.00 | 0.01 |
| SK-13-age1-27649 | 2013 | Trawl | Age 1 & 2 | 1.00 | 0.00 | 0.00 |  | 0.99 | 0.01 |  | 1.00 | 0.00 | 0.00 |
| SK-13-age1-27673 | 2013 | Trawl | Age 1 & 2 | 1.00 | 0.00 | 0.00 |  | 0.99 | 0.01 |  | 1.00 | 0.00 | 0.00 |
| SK-13-age1-27679 | 2013 | Trawl | Age 1 & 2 | 1.00 | 0.00 | 0.00 |  | 0.99 | 0.01 |  | 1.00 | 0.00 | 0.00 |
| SK-13-age1-27682 | 2013 | Trawl | Age 1 & 2 | 1.00 | 0.00 | 0.00 |  | 0.98 | 0.02 |  | 0.92 | 0.00 | 0.08 |
| SK-13-age1-27690 | 2013 | Trawl | Age 1 & 2 | 1.00 | 0.00 | 0.00 |  | 0.98 | 0.02 |  | 0.99 | 0.00 | 0.01 |
| SK-13-age1-27706 | 2013 | Trawl | Age 1 & 2 | 1.00 | 0.00 | 0.00 |  | 0.97 | 0.03 |  | 0.82 | 0.00 | 0.18 |
| SK-13-age1-27707 | 2013 | Trawl | Age 1 & 2 | 1.00 | 0.00 | 0.00 |  | 0.98 | 0.02 |  | 0.99 | 0.00 | 0.01 |
| SK-13-age1-27708 | 2013 | Trawl | Age 1 & 2 | 1.00 | 0.00 | 0.00 |  | 0.99 | 0.01 |  | 0.99 | 0.00 | 0.01 |
| SK-13-age1-27709 | 2013 | Trawl | Age 1 & 2 | 1.00 | 0.00 | 0.00 |  | 0.99 | 0.01 |  | 0.99 | 0.00 | 0.01 |
| SK-13-age1-27717 | 2013 | Trawl | Age 1 & 2 | 1.00 | 0.00 | 0.00 |  | 0.99 | 0.01 |  | 0.99 | 0.00 | 0.01 |
| SK-13-age1-27728 | 2013 | Trawl | Age 1 & 2 | 1.00 | 0.00 | 0.00 |  | 0.99 | 0.01 |  | 1.00 | 0.00 | 0.00 |
| SK-13-age1-27740 | 2013 | Trawl | Age 1 & 2 | 1.00 | 0.00 | 0.00 |  | 0.99 | 0.01 |  | 1.00 | 0.00 | 0.00 |
| SK-13-age1-27759 | 2013 | Trawl | Age 1 & 2 | 1.00 | 0.00 | 0.00 |  | 0.98 | 0.02 |  | 0.98 | 0.00 | 0.02 |
| SK-13-age1-27762 | 2013 | Trawl | Age 1 & 2 | 1.00 | 0.00 | 0.00 |  | 0.96 | 0.04 |  | 0.71 | 0.00 | 0.29 |
| SK-13-age1-27763 | 2013 | Trawl | Age 1 & 2 | 1.00 | 0.00 | 0.00 |  | 0.98 | 0.02 |  | 0.99 | 0.00 | 0.01 |
| SK-13-age1-27789 | 2013 | Trawl | Age 1 & 2 | 0.29 | 0.00 | 0.71 |  | 0.75 | 0.25 |  | 0.03 | 0.00 | 0.97 |
| SK-13-age1-27811 | 2013 | Trawl | Age 1 & 2 | 1.00 | 0.00 | 0.00 |  | 0.87 | 0.13 |  | 0.41 | 0.00 | 0.59 |
| SK-13-age1-27826 | 2013 | Trawl | Age 1 & 2 | 1.00 | 0.00 | 0.00 |  | 0.98 | 0.02 |  | 0.99 | 0.00 | 0.01 |
| SK-13-age1-27861 | 2013 | Trawl | Age 1 & 2 | 1.00 | 0.00 | 0.00 |  | 0.97 | 0.03 |  | 0.95 | 0.00 | 0.05 |
| SK-13-age1-27922 | 2013 | Trawl | Age 1 & 2 | 0.06 | 0.00 | 0.94 |  | 0.58 | 0.42 |  | 0.02 | 0.00 | 0.98 |
| SK-13-age1-27932 | 2013 | Trawl | Age 1 & 2 | 1.00 | 0.00 | 0.00 |  | 1.00 | 0.01 |  | 1.00 | 0.00 | 0.00 |
| SK-13-age1-27941 | 2013 | Trawl | Age 1 & 2 | 0.99 | 0.00 | 0.01 |  | 0.91 | 0.09 |  | 0.53 | 0.00 | 0.47 |
| SK-13-age1-27945 | 2013 | Trawl | Age 1 & 2 | 1.00 | 0.00 | 0.00 |  | 0.99 | 0.01 |  | 1.00 | 0.00 | 0.00 |
| SK-13-age1-27951 | 2013 | Trawl | Age 1 & 2 | 1.00 | 0.00 | 0.00 |  | 0.99 | 0.01 |  | 1.00 | 0.00 | 0.00 |
| SK-13-age1-27994 | 2013 | Trawl | Age 1 & 2 | 1.00 | 0.00 | 0.00 |  | 0.99 | 0.01 |  | 1.00 | 0.00 | 0.00 |
| SK-13-age1-28530 | 2013 | Trawl | Age 1 & 2 | 0.29 | 0.00 | 0.71 |  | 0.80 | 0.20 |  | 0.08 | 0.00 | 0.92 |
| SK-13-age1-28535 | 2013 | Trawl | Age 1 & 2 | 0.00 | 0.42 | 0.58 |  | 0.24 | 0.77 |  | 0.00 | 0.25 | 0.75 |
| SK-13-age1-28543 | 2013 | Trawl | Age 1 & 2 | 1.00 | 0.00 | 0.00 |  | 0.99 | 0.01 |  | 1.00 | 0.00 | 0.00 |
| SK-13-age1-28718 | 2013 | Trawl | Age 1 & 2 | 0.01 | 0.13 | 0.86 |  | 0.28 | 0.72 |  | 0.00 | 0.02 | 0.98 |
| SK-13-age1-28727 | 2013 | Trawl | Age 1 & 2 | 1.00 | 0.00 | 0.00 |  | 0.97 | 0.03 |  | 0.97 | 0.00 | 0.03 |
| SK-13-age1-28898 | 2013 | Trawl | Age 1 & 2 | 1.00 | 0.00 | 0.00 |  | 0.97 | 0.03 |  | 0.98 | 0.00 | 0.02 |
| SK-13-age1-28904 | 2013 | Trawl | Age 1 & 2 | 1.00 | 0.00 | 0.00 |  | 0.99 | 0.01 |  | 1.00 | 0.00 | 0.00 |
| SK-13-age1-29141 | 2013 | Trawl | Age 1 & 2 | 1.00 | 0.00 | 0.00 |  | 0.96 | 0.04 |  | 0.93 | 0.00 | 0.07 |
| SK-13-age1-29433 | 2013 | Trawl | Age 1 & 2 | 0.46 | 0.00 | 0.54 |  | 0.74 | 0.26 |  | 0.01 | 0.00 | 0.99 |
| SK-13-age1-29491 | 2013 | Trawl | Age 1 & 2 | 1.00 | 0.00 | 0.00 |  | 0.97 | 0.03 |  | 0.97 | 0.00 | 0.03 |
| SK-13-age1-29495 | 2013 | Trawl | Age 1 & 2 | 1.00 | 0.00 | 0.00 |  | 0.97 | 0.03 |  | 0.82 | 0.00 | 0.18 |
| SK-13-age1-29809 | 2013 | Trawl | Age 1 & 2 | 1.00 | 0.00 | 0.00 |  | 0.96 | 0.04 |  | 0.72 | 0.00 | 0.28 |
| SK-13-age1-29810 | 2013 | Trawl | Age 1 & 2 | 0.98 | 0.00 | 0.02 |  | 0.94 | 0.06 |  | 0.62 | 0.00 | 0.38 |
| SK-13-age1-29816 | 2013 | Trawl | Age 1 & 2 | 1.00 | 0.00 | 0.00 |  | 0.99 | 0.01 |  | 1.00 | 0.00 | 0.00 |
| SK-13-age1-29822 | 2013 | Trawl | Age 1 & 2 | 1.00 | 0.00 | 0.00 |  | 0.99 | 0.01 |  | 1.00 | 0.00 | 0.00 |
| SK-13-age1-29834 | 2013 | Trawl | Age 1 & 2 | 1.00 | 0.00 | 0.00 |  | 0.98 | 0.02 |  | 0.98 | 0.00 | 0.02 |
| SK-13-age1-29843 | 2013 | Trawl | Age 1 & 2 | 1.00 | 0.00 | 0.00 |  | 0.99 | 0.01 |  | 1.00 | 0.00 | 0.00 |
| SK-13-age1-29844 | 2013 | Trawl | Age 1 & 2 | 1.00 | 0.00 | 0.00 |  | 0.96 | 0.04 |  | 0.87 | 0.00 | 0.13 |
| SK-13-age1-29847 | 2013 | Trawl | Age 1 & 2 | 1.00 | 0.00 | 0.00 |  | 0.98 | 0.02 |  | 0.99 | 0.00 | 0.01 |
| SK-13-age1-27710 | 2013 | Trawl | Age 1 & 2 | 1.00 | 0.00 | 0.00 |  | 0.99 | 0.01 |  | 1.00 | 0.00 | 0.00 |
| SK-13-age1-27711 | 2013 | Trawl | Age 1 & 2 | 1.00 | 0.00 | 0.00 |  | 1.00 | 0.01 |  | 1.00 | 0.00 | 0.00 |
| SK-13-age1-27712 | 2013 | Trawl | Age 1 & 2 | 1.00 | 0.00 | 0.00 |  | 0.97 | 0.03 |  | 0.92 | 0.00 | 0.08 |
| SK-13-age1-27713 | 2013 | Trawl | Age 1 & 2 | 1.00 | 0.00 | 0.00 |  | 1.00 | 0.01 |  | 1.00 | 0.00 | 0.00 |
| SK-13-age1-27714 | 2013 | Trawl | Age 1 & 2 | 1.00 | 0.00 | 0.00 |  | 0.99 | 0.01 |  | 1.00 | 0.00 | 0.00 |
| SK-13-age1-27715 | 2013 | Trawl | Age 1 & 2 | 1.00 | 0.00 | 0.00 |  | 0.98 | 0.02 |  | 1.00 | 0.00 | 0.00 |
| SK-13-age1-27716 | 2013 | Trawl | Age 1 & 2 | 1.00 | 0.00 | 0.00 |  | 0.95 | 0.05 |  | 0.86 | 0.00 | 0.14 |
| SK-13-age1-27720 | 2013 | Trawl | Age 1 & 2 | 1.00 | 0.00 | 0.00 |  | 0.99 | 0.01 |  | 0.98 | 0.00 | 0.02 |
| SK-13-age1-27722 | 2013 | Trawl | Age 1 & 2 | 1.00 | 0.00 | 0.00 |  | 0.99 | 0.01 |  | 1.00 | 0.00 | 0.00 |
| SK-13-age1-27723 | 2013 | Trawl | Age 1 & 2 | 1.00 | 0.00 | 0.00 |  | 0.97 | 0.03 |  | 0.99 | 0.00 | 0.01 |
| SK-13-age1-27724 | 2013 | Trawl | Age 1 & 2 | 1.00 | 0.00 | 0.00 |  | 0.99 | 0.01 |  | 1.00 | 0.00 | 0.00 |
| SK-13-age1-27780 | 2013 | Trawl | Age 1 & 2 | 1.00 | 0.00 | 0.00 |  | 0.98 | 0.02 |  | 0.96 | 0.00 | 0.04 |
| SK-13-age1-27781 | 2013 | Trawl | Age 1 & 2 | 1.00 | 0.00 | 0.00 |  | 0.95 | 0.05 |  | 0.77 | 0.00 | 0.23 |
| SK-13-age1-27784 | 2013 | Trawl | Age 1 & 2 | 0.99 | 0.00 | 0.01 |  | 0.98 | 0.02 |  | 0.72 | 0.00 | 0.28 |
| SK-13-age1-27787 | 2013 | Trawl | Age 1 & 2 | 1.00 | 0.00 | 0.00 |  | 0.99 | 0.01 |  | 1.00 | 0.00 | 0.00 |
| SK-13-age1-27788 | 2013 | Trawl | Age 1 & 2 | 1.00 | 0.00 | 0.00 |  | 0.99 | 0.01 |  | 1.00 | 0.00 | 0.00 |
| SK-13-age1-27790 | 2013 | Trawl | Age 1 & 2 | 1.00 | 0.00 | 0.00 |  | 0.98 | 0.02 |  | 0.97 | 0.00 | 0.03 |
| SK-13-age1-27845 | 2013 | Trawl | Age 1 & 2 | 1.00 | 0.00 | 0.00 |  | 1.00 | 0.01 |  | 1.00 | 0.00 | 0.00 |
| SK-13-age1-27848 | 2013 | Trawl | Age 1 & 2 | 1.00 | 0.00 | 0.00 |  | 0.97 | 0.03 |  | 0.95 | 0.00 | 0.05 |
| SK-13-age1-27872 | 2013 | Trawl | Age 1 & 2 | 1.00 | 0.00 | 0.00 |  | 0.98 | 0.02 |  | 0.98 | 0.00 | 0.02 |
| SK-13-age1-27873 | 2013 | Trawl | Age 1 & 2 | 1.00 | 0.00 | 0.00 |  | 0.98 | 0.02 |  | 1.00 | 0.00 | 0.00 |
| SK-13-age1-27874 | 2013 | Trawl | Age 1 & 2 | 1.00 | 0.00 | 0.00 |  | 0.99 | 0.01 |  | 0.99 | 0.00 | 0.01 |
| SK-13-age1-27875 | 2013 | Trawl | Age 1 & 2 | 1.00 | 0.00 | 0.00 |  | 0.94 | 0.07 |  | 0.74 | 0.00 | 0.26 |
| SK-13-age1-27878 | 2013 | Trawl | Age 1 & 2 | 1.00 | 0.00 | 0.00 |  | 1.00 | 0.01 |  | 1.00 | 0.00 | 0.00 |
| SK-13-age1-27879 | 2013 | Trawl | Age 1 & 2 | 1.00 | 0.00 | 0.00 |  | 0.98 | 0.02 |  | 0.96 | 0.00 | 0.04 |
| SK-13-age1-27880 | 2013 | Trawl | Age 1 & 2 | 1.00 | 0.00 | 0.00 |  | 0.99 | 0.01 |  | 1.00 | 0.00 | 0.00 |
| SK-13-age1-27881 | 2013 | Trawl | Age 1 & 2 | 1.00 | 0.00 | 0.00 |  | 0.99 | 0.01 |  | 1.00 | 0.00 | 0.00 |
| SK-13-age1-27882 | 2013 | Trawl | Age 1 & 2 | 1.00 | 0.00 | 0.00 |  | 0.97 | 0.03 |  | 0.95 | 0.00 | 0.05 |
| SK-13-age1-27883 | 2013 | Trawl | Age 1 & 2 | 1.00 | 0.00 | 0.00 |  | 0.98 | 0.02 |  | 1.00 | 0.00 | 0.00 |
| SK-13-age1-27884 | 2013 | Trawl | Age 1 & 2 | 1.00 | 0.00 | 0.00 |  | 0.99 | 0.01 |  | 1.00 | 0.00 | 0.00 |
| SK-13-age1-27885 | 2013 | Trawl | Age 1 & 2 | 1.00 | 0.00 | 0.00 |  | 0.99 | 0.01 |  | 1.00 | 0.00 | 0.00 |
| SK-13-age1-27892 | 2013 | Trawl | Age 1 & 2 | 1.00 | 0.00 | 0.00 |  | 0.99 | 0.01 |  | 1.00 | 0.00 | 0.00 |
| SK-13-age1-27966 | 2013 | Trawl | Age 1 & 2 | 1.00 | 0.00 | 0.00 |  | 0.98 | 0.02 |  | 0.99 | 0.00 | 0.01 |
| SK-13-age1-27967 | 2013 | Trawl | Age 1 & 2 | 1.00 | 0.00 | 0.00 |  | 0.99 | 0.01 |  | 1.00 | 0.00 | 0.00 |
| SK-13-age1-27968 | 2013 | Trawl | Age 1 & 2 | 0.99 | 0.00 | 0.01 |  | 0.83 | 0.17 |  | 0.22 | 0.00 | 0.78 |
| SK-13-age1-27969 | 2013 | Trawl | Age 1 & 2 | 1.00 | 0.00 | 0.00 |  | 0.99 | 0.01 |  | 1.00 | 0.00 | 0.00 |
| SK-13-age1-27970 | 2013 | Trawl | Age 1 & 2 | 1.00 | 0.00 | 0.00 |  | 0.98 | 0.02 |  | 0.98 | 0.00 | 0.02 |
| SK-13-age1-27971 | 2013 | Trawl | Age 1 & 2 | 1.00 | 0.00 | 0.00 |  | 0.98 | 0.02 |  | 0.97 | 0.00 | 0.03 |
| SK-13-age1-27972 | 2013 | Trawl | Age 1 & 2 | 1.00 | 0.00 | 0.00 |  | 0.98 | 0.02 |  | 0.99 | 0.00 | 0.01 |
| SK-13-age1-27973 | 2013 | Trawl | Age 1 & 2 | 0.99 | 0.00 | 0.01 |  | 0.91 | 0.09 |  | 0.61 | 0.00 | 0.39 |
| SK-13-age1-27974 | 2013 | Trawl | Age 1 & 2 | 1.00 | 0.00 | 0.00 |  | 0.98 | 0.02 |  | 0.98 | 0.00 | 0.02 |
| SK-13-age1-27998 | 2013 | Trawl | Age 1 & 2 | 1.00 | 0.00 | 0.00 |  | 0.98 | 0.02 |  | 0.99 | 0.00 | 0.01 |
| Skaha_Creel_2015_01 | 2015 | Angler | Age 2+ | 1.00 | 0.00 | 0.00 |  | 0.99 | 0.01 |  | 0.98 | 0.00 | 0.02 |
| Skaha_Creel_2015_02 | 2015 | Angler | Age 4+ | 1.00 | 0.00 | 0.00 |  | 0.95 | 0.05 |  | 0.97 | 0.00 | 0.03 |
| Skaha_Creel_2015_04 | 2015 | Angler | Age 3+ | 0.84 | 0.00 | 0.16 |  | 0.93 | 0.07 |  | 0.21 | 0.00 | 0.79 |
| Skaha_Creel_2015_05 | 2015 | Angler | Unknown | 1.00 | 0.00 | 0.00 |  | 0.98 | 0.03 |  | 0.99 | 0.00 | 0.01 |
| Skaha_Creel_2015_06 | 2015 | Angler | Unknown | 1.00 | 0.00 | 0.00 |  | 0.99 | 0.01 |  | 1.00 | 0.00 | 0.00 |
| Skaha_Creel_2015_08 | 2015 | Angler | Age 3+ | 0.88 | 0.00 | 0.12 |  | 0.84 | 0.16 |  | 0.11 | 0.00 | 0.89 |
| Skaha_Creel_2015_09 | 2015 | Angler | Age 3+ | 0.96 | 0.00 | 0.04 |  | 0.95 | 0.05 |  | 0.61 | 0.00 | 0.39 |
| Skaha_Creel_2015_10 | 2015 | Angler | Age 3+ | 1.00 | 0.00 | 0.00 |  | 0.99 | 0.01 |  | 1.00 | 0.00 | 0.00 |
| Skaha_Creel_2015_11 | 2015 | Angler | Unknown | 1.00 | 0.00 | 0.00 |  | 0.97 | 0.03 |  | 0.96 | 0.00 | 0.04 |
| Skaha_Creel_2015_12 | 2015 | Angler | Unknown | 0.99 | 0.00 | 0.01 |  | 0.82 | 0.18 |  | 0.11 | 0.00 | 0.89 |
| Skaha_Creel_2015_13 | 2015 | Angler | Age 2+ | 0.56 | 0.00 | 0.44 |  | 0.70 | 0.30 |  | 0.04 | 0.00 | 0.96 |
| Skaha_Creel_2015_17 | 2015 | Angler | Age 3+ | 1.00 | 0.00 | 0.00 |  | 0.97 | 0.04 |  | 0.97 | 0.00 | 0.03 |
| Skaha_Creel_2015_18 | 2015 | Angler | Age 4+ | 1.00 | 0.00 | 0.00 |  | 0.99 | 0.02 |  | 0.98 | 0.00 | 0.02 |
| Skaha_Creel_2015_19 | 2015 | Angler | Unknown | 0.85 | 0.00 | 0.15 |  | 0.89 | 0.11 |  | 0.22 | 0.00 | 0.78 |
| Skaha_Creel_2015_20 | 2015 | Angler | Age 4+ | 0.97 | 0.00 | 0.03 |  | 0.92 | 0.08 |  | 0.58 | 0.00 | 0.42 |
| Skaha_Creel_2015_21 | 2015 | Angler | Age 2+ | 0.36 | 0.00 | 0.64 |  | 0.74 | 0.26 |  | 0.01 | 0.00 | 0.99 |
| Skaha_Creel_2015_22 | 2015 | Angler | Age 3+ | 0.75 | 0.00 | 0.25 |  | 0.90 | 0.10 |  | 0.27 | 0.00 | 0.73 |
| Skaha_Creel_2015_23 | 2015 | Angler | Age 4+ | 1.00 | 0.00 | 0.00 |  | 0.98 | 0.02 |  | 0.97 | 0.00 | 0.03 |
| Skaha_Creel_2015_24 | 2015 | Angler | Age 2+ | 0.53 | 0.00 | 0.47 |  | 0.71 | 0.29 |  | 0.12 | 0.00 | 0.88 |
| Skaha_Creel_2015_28 | 2015 | Angler | Unknown | 0.99 | 0.00 | 0.01 |  | 0.96 | 0.04 |  | 0.70 | 0.00 | 0.30 |
| Skaha_Creel_2015_29 | 2015 | Angler | Unknown | 1.00 | 0.00 | 0.00 |  | 0.99 | 0.01 |  | 1.00 | 0.00 | 0.00 |
| Skaha_Creel_2015_30 | 2015 | Angler | Age 5+ | 0.99 | 0.00 | 0.01 |  | 0.94 | 0.06 |  | 0.67 | 0.00 | 0.33 |
| Skaha_Creel_2015_32 | 2015 | Angler | Unknown | 1.00 | 0.00 | 0.00 |  | 0.98 | 0.03 |  | 0.97 | 0.00 | 0.03 |
| Skaha_Creel_2015_36 | 2015 | Angler | Unknown | 0.28 | 0.00 | 0.72 |  | 0.82 | 0.18 |  | 0.03 | 0.00 | 0.97 |
| Skaha_Creel_2015_39 | 2015 | Angler | Age 3+ | 0.00 | 0.01 | 0.98 |  | 0.32 | 0.68 |  | 0.00 | 0.01 | 0.99 |
| Skaha_Creel_2015_45 | 2015 | Angler | Age 3+ | 0.55 | 0.00 | 0.45 |  | 0.76 | 0.24 |  | 0.05 | 0.00 | 0.95 |
| Skaha_Creel_2015_47 | 2015 | Angler | Age 3+ | 1.00 | 0.00 | 0.00 |  | 0.94 | 0.06 |  | 0.77 | 0.00 | 0.23 |
| Skaha_Creel_2015_49 | 2015 | Angler | Age 3+ | 0.99 | 0.00 | 0.01 |  | 0.89 | 0.11 |  | 0.45 | 0.00 | 0.55 |
| Skaha_Creel_2015_50 | 2015 | Angler | Age 3+ | 0.09 | 0.12 | 0.79 |  | 0.41 | 0.59 |  | 0.00 | 0.00 | 1.00 |
| Skaha_Creel_2015_51 | 2015 | Angler | Unknown | 1.00 | 0.00 | 0.00 |  | 0.99 | 0.02 |  | 0.98 | 0.00 | 0.02 |
| Skaha_Creel_2015_57 | 2015 | Angler | Age 3+ | 0.98 | 0.00 | 0.02 |  | 0.93 | 0.07 |  | 0.48 | 0.00 | 0.52 |
| Skaha_Creel_2015_59 | 2015 | Angler | Unknown | 1.00 | 0.00 | 0.00 |  | 0.94 | 0.07 |  | 0.69 | 0.00 | 0.31 |
| Skaha_Creel_2015_61 | 2015 | Angler | Age 3+ | 0.22 | 0.00 | 0.78 |  | 0.65 | 0.35 |  | 0.01 | 0.00 | 0.99 |
| Skaha_Creel_2015_62 | 2015 | Angler | Age 3+ | 0.02 | 0.02 | 0.96 |  | 0.37 | 0.63 |  | 0.00 | 0.02 | 0.98 |
| Skaha_Creel_2015_63 | 2015 | Angler | Age 2+ | 1.00 | 0.00 | 0.00 |  | 0.98 | 0.02 |  | 0.96 | 0.00 | 0.04 |
| Skaha_Creel_2015_65 | 2015 | Angler | Age 3+ | 0.01 | 0.14 | 0.85 |  | 0.28 | 0.72 |  | 0.00 | 0.04 | 0.96 |
| Skaha_Creel_2015_66 | 2015 | Angler | Age 3+ | 1.00 | 0.00 | 0.00 |  | 0.98 | 0.02 |  | 0.95 | 0.00 | 0.05 |
| Skaha_Creel_2015_67 | 2015 | Angler | Unknown | 1.00 | 0.00 | 0.00 |  | 0.98 | 0.02 |  | 0.99 | 0.00 | 0.01 |
| Skaha_Creel_2015_71 | 2015 | Angler | Age 3+ | 1.00 | 0.00 | 0.00 |  | 0.95 | 0.05 |  | 0.88 | 0.00 | 0.12 |
| Skaha_Creel_2015_73 | 2015 | Angler | Unknown | 1.00 | 0.00 | 0.00 |  | 0.98 | 0.02 |  | 0.97 | 0.00 | 0.03 |
| Skaha_Creel_2015_76 | 2015 | Angler | Age 2+ | 1.00 | 0.00 | 0.00 |  | 0.98 | 0.03 |  | 0.96 | 0.00 | 0.04 |
| Skaha_Creel_2015_78 | 2015 | Angler | Age 3+ | 1.00 | 0.00 | 0.00 |  | 0.88 | 0.12 |  | 0.35 | 0.00 | 0.65 |
| Skaha_Creel_2015_79 | 2015 | Angler | Unknown | 1.00 | 0.00 | 0.00 |  | 0.94 | 0.06 |  | 0.76 | 0.00 | 0.24 |
| Skaha_Creel_2015_83 | 2015 | Angler | Age 4+ | 0.99 | 0.00 | 0.01 |  | 0.94 | 0.06 |  | 0.81 | 0.00 | 0.19 |
| Skaha_Creel_2015_84 | 2015 | Angler | Unknown | 0.34 | 0.01 | 0.65 |  | 0.61 | 0.39 |  | 0.00 | 0.00 | 1.00 |
